# A systematic review identifying effective teaching methods and their combinations for increasing empathy in physicians: pairwise and network meta-analysis

**DOI:** 10.1186/s12909-025-07917-x

**Published:** 2025-10-23

**Authors:** Hazel Ngo, Nina Sokolovic, Julia Hu, Jennifer Jenkins

**Affiliations:** University of Toronto, 252 Bloor St W, Toronto, ON M5S 1V6 Canada

**Keywords:** Empathy, Meta-analysis, Physician, Intervention, Medical education

## Abstract

**Background:**

Demonstrating empathy is fundamental for providing patient-centered care, however, what components are most effective is not known. Thus, the aim of this study was to identify key teaching methods and intervention characteristics for increasing empathy skills across medical training.

**Method:**

This study was part of a larger systematic review, which was pre-registered: CRD42018100100. We performed a systematic review, pairwise meta-analysis (PMA) and network meta-analysis (NMA). A systematic search was used across PsycINFO, Medline, CINAHL, Social Work Abstracts, ERIC, ABI/INFORM, and the Cochrane Central Register of Controlled Trials from the inception of the respective databases to October 9, 2022. Studies included randomized controlled trials (RCTs) examining behavioural interventions which targeted empathy skills for physicians and medical students. Studies were excluded if reported summary data could not be converted to an effect size; if the author were unable to be contacted; and if the study did not compare substantively different intervention combinations. Risk of bias was assessed using the Cochrane risk-of-bias tool. Data were pooled using random-effects PMA and NMA.

**Results:**

308 full-text studies were found, of which 111 met the inclusion criteria, totalling 11,111 participants. Overall, a medium effect of interventions was found [*d* = 0.50 (95% CI = 0.40, 0.60)], meaning the empathy skills of participants improved moderately compared to those in control groups. Publication bias was evident and heterogeneity was high (*I*^2^ = 79.19, *p* < .001). Subgroup analyses of the PMA revealed the following moderators were statistically significant: teaching method, intervention formats, control group; measurement type, and number of teaching methods used. The consistency assumption was met [χ^2^ [ (33)= 39.11, *p* = .21] for the NMA. The NMA revealed that didactic and rehearsal were most frequently included among the most effective teaching method combinations.

**Conclusions:**

By using PMA and NMA, we provide novel insights on effective intervention components for improving empathy in medicine. To improve aggregation of evidence, transparent and standardized reporting from studies may help reduce heterogeneity. Overall, our results support the notion that interventions need not be expensive nor prolonged to be effective.

**Supplementary Information:**

The online version contains supplementary material available at 10.1186/s12909-025-07917-x.

## Background

Demonstrating empathy is an essential component of delivering high quality, patient-centered care [1, 2]. Competence in medicine involves more than just treating the illness itself, but addressing the needs of the patient [3, 4]. Empathy in medicine can be defined as a skilled, intentional response which involves the perspective-taking ability (i.e., understanding the inner experiences, thoughts, and feelings of the patient) and an appropriate response to the patient, such as checking for a patient’s understanding or helping a patient feel more comfortable [5, 6]. Empathy is delineated from sympathy or compassion, which involve vicarious, reactive responses and are associated with burnout [7, 8].

For patients, receiving empathetic care from physicians is associated with higher satisfaction [9], greater treatment compliance, improved clinical outcomes, and decreased psychological distress [2, 10]. For physicians, empathy is protective against burnout [11, 12]. There is, however, an “empathy crisis” within healthcare [13], such that physician empathy has been shown to decline in medical training and stay low [14, 15]. Part of addressing this clinical gap requires understanding how to optimize the design of empathy interventions. How can we teach empathy to physicians in a way that is both effective and efficient?

What is currently known is that empathy is a trainable skill among healthcare professionals, including physicians, as shown across past meta-analyses [16–18]. In general, behavioural interventions, including ones targeting empathy, use the following five teaching methods, singly or in combination: didactic (lecture and reading and/or written information), rehearsal (practicing the empathy skill and/or role-playing as the patient), reflection (reflecting by oneself or in discussion with others), observation (watching enactments pertaining to the skill and/or exposure to a patient’s perspective), and feedback (individualized from peer or facilitator) [19, 20]. See Table 1 for more detail.

**Table 1:** Teaching methods and its relation to type of learning and theory

Teaching Method	Description and examples	Type of Learning^a^	Theoretical Basis of Teaching Method^b^	Teaching Empathy in Medicine^b^
Didactic	Oral or written information about empathetic interactions (e.g., presentation, pamphlet or workbook with relevant information)	Surface Learning	Cognitive learning theories emphasize the importance of developing new cognitive schemas and expand upon previous schemas (Tennyson & Rasch, 1998).	Learning empathy skills requires having theoretical knowledge before being able to apply skills (Assing Hvidt et al., 2022)
Rehearsal	Participants had the opportunity to practice empathy-skills (e.g., role play exercises, practice directly with clients)	Surface & Deep Learning	Experiential learning theories emphasize the necessity of “grasping and transforming experiences” to consolidate learning (Kolb, Boyatzis, & Mainemelis, 2014).	Empathy is a form of emotional labor hat requires effortful role-taking processes, notably what is required in “acting” (Larson & Xin, 2005)
Reflection	Participants had the opportunity to reflect on and discuss ideas of empathy and their own experiences interacting with clients, either with other participants or facilitators.	Deep Learning	Transformative learning theories emphasize critical reflective practices as necessary to challenge previous knowledge and make meaning from experiences (Mezirow, 1997)	Developing reflective capacity can deepen physician’s empathy towards patients (Wald & Reis, 2010)
Observation	Participants watch or listen to others interacting and/or performing the skill they are trying to learn (e.g., video vignettes, live demonstration)	Surface Learning	Social learning theory focus on how watching and imitating models is important for learning a new skill or behaviour (Bandura & Walters, 1977)	Watching a positive role model is an effective teaching tool to consciously and unconsciously learn key clinical skills like empathy (Passi & Johnson, 2016)
Feedback	Participants received feedback (from other participants and/or facilitators) about their performance exhibiting empathy in interactions	Surface & Deep Learning	Providing constructive, personalized feedback is supported as an effective approach for learning (Thurlings et al., 2013)	Providing constructive feedback helps healthcare trainees with empathy skills, including affect recognition (Zec & Forrest, 2019)

^a^Surface and deep learning are terms referenced from Hattie & Denoghue, 2016 [21]

^b^See full references in Supplemental Appendix 10

What is not yet known is what constitutes an *effective* empathy intervention, as there is currently no standard for teaching empathy within medical education [22]. Previous meta-analyses show mixed support for different teaching methods. A recent meta-analysis of 18 randomized controlled trials (RCT) of empathy interventions for medical students found that none of the individual teaching methods were more effective than the control condition, although a trend was evident for rehearsal [18]. Similarly, no teaching methods appeared to be significantly effective in a sample of mixed healthcare professionals [16]. However, meta-analyses of empathy interventions for mental health practitioners found observational [23] and didactic [24] approaches to be the most effective. Furthermore, it is unclear whether combining more or fewer teaching methods into a single intervention impacts effectiveness. In the context of teaching empathy to adults, two meta-analyses support the using *more* teaching methods [18, 23] while two other meta-analyses suggest the number of teaching methods may not matter [24, 25].

Beyond ascertaining *which* and *how many* teaching methods are superior, it is also important to know the precise *combination* of teaching methods that are effective for teaching empathy. Teaching methods may combine such that their sum is greater than their parts. For example, didactic teaching methods may be more effective combined with rehearsal than reflection. Traditional pairwise meta-analyses (PMA) cannot answer this question as it is limited its analytical power to compare all possible interventions to each other. Network meta-analysis (NMA), however, can provide additional depth. First, NMA provides a relative ranking of multiple interventions in a single analysis (treatment A versus treatment B versus treatment C etc.), whereas traditional PMA is constrained to two (i.e., treatment versus control). This allows for the comparison of different intervention types, including combinations of teaching methods, revealing superior performing interventions [26, 27].

Second, whereas PMA relies only on direct evidence (i.e., head-to-head comparisons), NMA leverages both direct and indirect evidence (i.e., comparisons across interventions that have not been directly compared in studies). Third, NMA can include studies with more than two intervention arms. With these strengths, NMA has been increasingly used for clinical decision-making [28–30]. For example, researchers have recently used NMA to compare the clinical efficacy of different COVID-19 vaccines [31] and determine the best intervention for the treatment of depression [29]. NMA is a powerful analytic tool which can provide nuanced information regarding intervention effectiveness. As there are substantial logistical and economic implications when designing curricula and training programs, further clarity on the relative effectiveness of interventions is needed.

Thus, the current study seeks to address these critical empirical and practical gaps in two ways. Using the PMA technique, we investigate which “ingredients” constitute effective empathy interventions in physicians. That is, which *teaching methods* and *intervention characteristics* optimize effect sizes compared to control conditions? Various study characteristics (i.e., measurement type, type of control group, education level) were examined, as previous evidence has either been inconclusive on these moderators or has yet not explored these potential moderators [16, 18, 25]. Using the NMA technique, we compared empathy intervention “recipes” to rank *combinations* of teaching methods. While this has been done for empathy-related interventions in parents [32] and mental health care practitioners [24], this study will be the first to use NMA in physicians and medical students.

## Methods

### Search strategy

A protocol for the overarching study was developed and registered with the International Prospective Register of Systematic Reviews (Protocol number: CRD42018100100). The current study was part of a larger search to broadly assess interventions for teaching adults, empathy-related skills. The current study represents a focused subset of that protocol, restricted to studies involving physicians and physicians-in training (i.e., medical students). Any substantial deviations from the registered protocol are noted in the “Amendments to Protocol” section. Other papers that derived from this search include network meta-analyses for mental health care practitioners [24] and parents [32] and a meta-analysis that focused on the differentiation between cognitive and affective empathy in physicians [33].

The search strategy was developed in consultation with librarians using the Peer Review of Electronic Search Strategies checklist [34]. The search was conducted in PsycINFO, Medline, CINAHL, Social Work Abstracts, ERIC, ABI/INFORM, and the Cochrane Central Register of Controlled Trials from the inception of the respective databases to October 9, 2022 (for search strategy, see Supplemental Appendix 1). Reference lists of both included studies and relevant review articles retrieved in the search were also scanned for additional studies. Grey and non-peer reviewed literature were included (e.g., dissertations, reports). We followed the frequently used practice of non-exclusion of grey literature to achieve broad representativeness [35]. For full search strategy and full study references, see Supplemental Appendix 1 and 3, respectively.

### Inclusion criteria

Studies that were included met the following criteria: randomized trial of a behavioural training targeting empathy skills (see below for detail on empathy definition); the sample is comprised of only physicians (i.e., professionals) and/or physicians-in-training (i.e., undergraduate or postgraduate medical school or residency); reports on at least one quantitative measure of empathy (e.g., self-report, other-report, observational scales, ability tests, or combinations of these); and the study is available in English (due to coding capacities).

Empathy was defined as the *accurate understanding and appropriate response to the cognitions*,* emotions*,* and behaviours of another.* Therefore, empathy skills in the medical context included related concepts of communication skills (e.g., active listening, avoiding jargon); interpersonal skills and emotional intelligence (e.g., making patient feel comfortable, accurate detection of patient distress); and patient-centered care (e.g., considering patient’s feelings in treatment decision, patient feeling understood). To control for non-independent sampling between studies, we screened studies to see if any shared the same sample; if they did and reported different outcomes, we followed the common practice of creating the mean of the two effects [36] (*k* = 2).

### Exclusion criteria

**Fig. 1:**
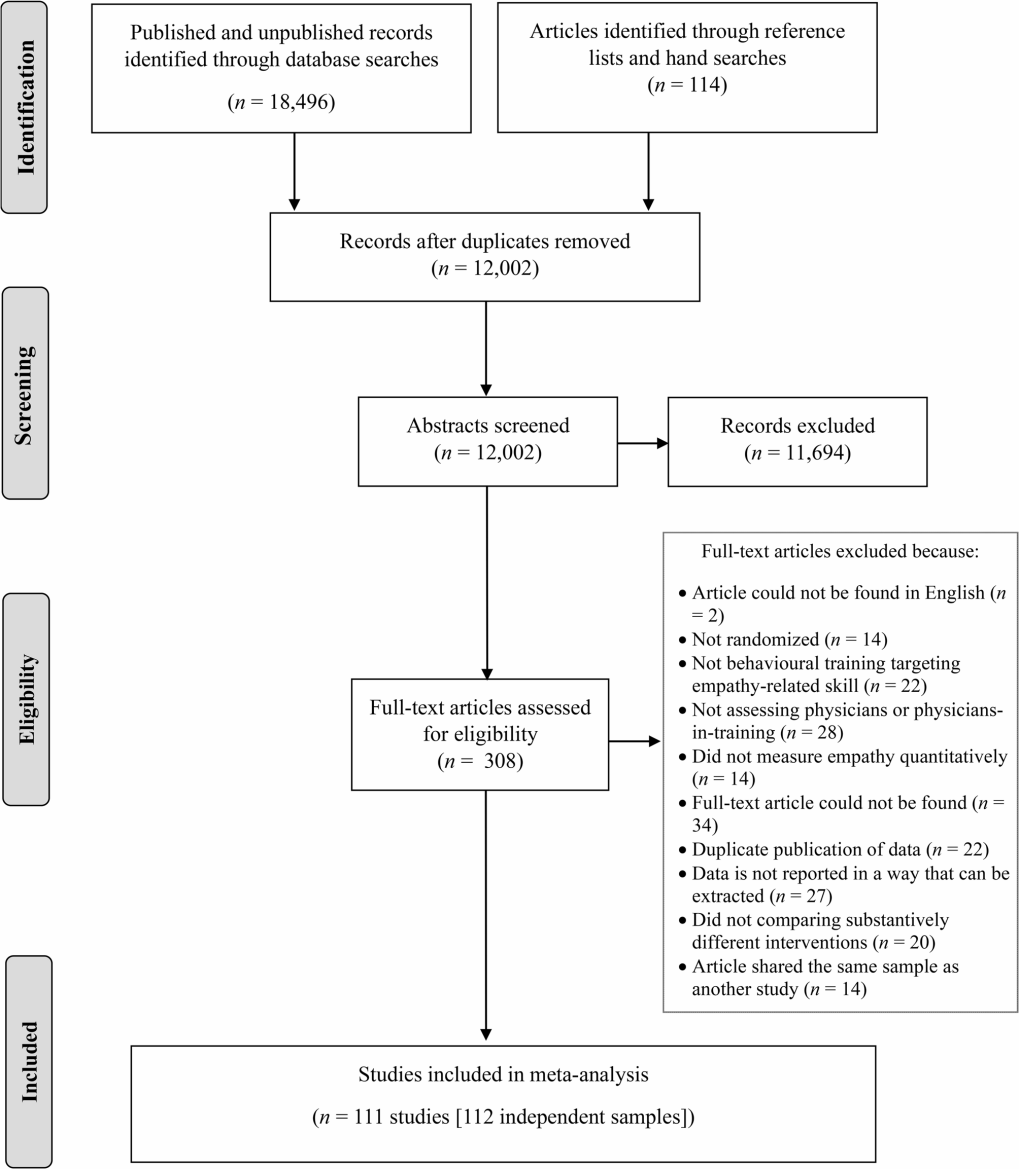
PRISMA flow diagram.

See fig. 1 for more details and *Additional File 1* for the list of excluded studies. We excluded studies which did not measure empathy as defined above (e.g., compassion, sympathy). For studies in which summary data was not reported in a way could be converted into an effect size, we contacted the author when an e-mail correspondence was available (28 contacts and 15 responses). If the author did not respond or were unable to be contacted, the study was excluded. Studies that did not compare substantively different interventions (i.e., the comparison intervention used same teaching methods) were excluded (*n* = 20). This is possible in cases where a study may be comparing two types of the teaching methods (e.g., both intervention arms compared didactic teaching methods, such as teaching with lectures versus reading materials).

### Data extraction and quality assessment

All coders were advanced undergraduate and graduate student research assistants, first trained to an interrater reliability over 80% (percent agreement). Independent pairs of coders screened abstracts and full-texts (Covidence software) and extracted data using a predefined data extraction sheet (Microsoft Excel), with full definitions of the study variables (Supplemental Appendix 4). Data were double-coded and all discrepancies were resolved by the first author (HN). Risk of bias at the study-level was assessed using the Cochrane risk-of-bias tool [37]. Coding criteria for risk of bias can be found in Supplemental Appendix 13. To assess for the certainty for evidence, we conducted a structured assessment at the outcome-level (empathy) across both the pairwise meta-analysis (PMA) and network meta-analysis (NMA). The assessment focused on five commonly used domains in evidence synthesis: risk of bias, inconsistency, indirectness, imprecision, and potential publication bias. See Supplemental Appendix 11 for full description and results.

### Outcome data items

Eligible quantitative outcomes of empathy could include any of the following categories: cognitive empathy, defined as “the accurate understanding and appropriate response to others’ thoughts”; affective empathy (not compassion, emotional resonance, or sympathy), defined as “the accurate understanding and appropriate response to others’ feelings”; and general empathy or perspective-taking (i.e., when the type of empathy was not specified, or a combination of cognitive and affective empathy).

Results could be reported as an overall score (scale), subscale score (e.g., affective empathy), or item score (e.g., “I try to understand what is going on in my patients’ minds”). In terms of time frame, there was no minimum time lapse between pre-test and post-test taken; if there were multiple post-test scores (i.e., there were follow-up sessions), the result within the closest time frame was extracted, as a vast majority of studies did not conduct a follow-up. For a measure to be extracted, at least 50% of the measure had to have items pertaining to empathy. If results were reported at multiple levels (i.e., scale, subscale, individual item), then results were extracted at the level which has the maximum number of total items, in which no less than 50% of those items are relevant. If no single subscale or scale has > 50% of items pertaining to empathy, results for each item was reported.

If multiple measurement types were reported, we selected objective measures (independent coder scores the physician’s performance, e.g., ability tests, standardized behavioral coding) over other-reported (the person interacting with the physician rates their ability, e.g., patient-rated checklists) and first-person measures (physician answers questions about their own ability, e.g., physician’s self-report of performance) as objective measures of empathy may be less biased [38] than self-reports. This ordering (i.e., prioritizing objective measures over patient-report, and patient-report over self-report) was chosen to minimize shared method variance (such as bias introduced by social desirability or self-perception) and thereby more accurately detect training-related changes in empathic skill [39]. A full definition of these measurement types is found in Supplemental Appendix 4.

If multiple measures were of the same methodological quality (e.g., multiple *objective* measures), a pooled effect size was calculated across these measures to obtain one effect size per comparison without artificially deflating the standard error [40]. See Supplemental Appendix 6 for list of extracted measures.

### Other data items

We collected data on the study (author, year of publication, country region, source of publication, sample size) and the participants (mean age, education level, gender, ethnicity). For the intervention design, we collected information on control group type, teaching method (e.g., didactic, rehearsal), number of teaching methods used, number of sessions, format type, and facilitator type. For definitions of each category of variables, see Supplemental Appendix 4.

### Statistical analysis

Comprehensive Meta-Analysis (CMA; Biostat, New Jersey, United States of America) software was used to calculate a standardized mean difference (SMD; Cohen’s *d*) effect size and standard error (SE) for each comparison. If a study did not report the SMD and SE, we converted or estimated the available data within CMA. For studies in which two of the arms were functionally equivalent (i.e., the arms used the same teaching methods and format of delivery), the mean effect size was calculated for those two intervention arms relative to the other arm(s) in the study (*k* = 5).

Pairwise meta-analysis was conducted in CMA using all studies that had a control group, including pure control (no intervention provided, e.g., waitlist control), active control (sham intervention, where format is similar to intervention but topic did not cover empathy-related skills, e.g., stress management), or “education as usual” (usual curriculum of medical communication skills, e.g., lecture-based training). A random-effects model was chosen as study populations were assumed to differ [36]. For studies with more than two functionally non-equivalent interventions (i.e., different combinations of teaching methods), we only used the comparison between the study arm with the greatest number of teaching methods (i.e., most intensive) and the control group [32]. Statistical heterogeneity was assessed with *I*^*2*^ the statistic and *p* value and explored using mixed-effects model meta-regressions using method of moments estimation [36]. Publication bias was assessed by inspecting funnel plots and running Egger’s test [41].

Random effects network meta-analysis was conducted, as study heterogeneity was assumed, using all study comparisons. Eligible empathy interventions were those consisting of any combination of teaching methods (described in Supplemental Appendix 4). The analysis was run in STATA SE (version 18; Stata Corp., College Station, Texas), using the network and network graphs packages [42]. Network geometry was explored visually using the network graph. The consistency assumption was evaluated using the design-by-treatment interaction model [43]. We conducted a random-effects network meta-analysis assuming a common within-network between-study variance (τ^2^), assessed through visual inspection of the network forest plot [44]. To reduce heterogeneity, any combination of teaching methods that was represented by a single study was dropped from the analysis. Results were reported as the SMD and the 95% confidence intervals (CI). To rank the relative efficacy of the different interventions, the surface under the cumulative ranking curve (SUCRA) values was used [45]. Reporting bias was assessed using a comparison-adjusted funnel-plot [42].

A sensitivity analysis was performed where combinations represented by a single study were included and instead the bottom SUCRA-ranked teaching method combinations were removed (*n* = 22). Two other sensitivity analyses were performed: grey literature was excluded (*n* = 3) and two outliers were excluded (*n* = 2). Details of the sensitivity analyses can be found in Supplemental Appendix 9. Data code and individual study information is available in Supplemental Appendix 2 and 6, respectively. Statistical significance was determined at *p* <.05 and marginal significance at *p* <.10.

## Results

As outlined in the trial flow diagram (Fig. 1), 12,002 citations were screened and 308 of those articles were further assessed for eligibility. The current study included data from 111 studies. Due to some studies overlapping in samples and some studies reporting independent samples, this resulted in 112 independent samples, totalling to 11,111 participants (See Supplemental Appendix 3 for details). Studies’ publication dates ranged from January 1971 to October 2022, though most (74/109, 68%) were published between 2010 and 2022 inclusive. Of the 77% of studies that reported gender (84/109), samples were approximately 50% female. Most studies did not report on the ethnicity of participants (84/109, 77%); however, those that did report had primarily European American samples (Supplemental Appendix 5).

### Amendments to the protocol

There were no major deviations from the registered protocol (CRD42018100100) with regards to the search strategy, eligibility criteria, outcomes of interest, data collection procedures, or planned analytic methods, including the use of network meta-analysis (NMA). However, while the protocol described the inclusion of “medical professionals,” the current study focuses specifically on physicians and medical students. This narrower focus was not explicitly specified in the protocol. The decision to restrict the sample emerged post hoc for two reasons: firstly, the large volume of eligible studies retrieved through the broader search and secondly, our discovery of the heterogeneity in outcomes as a function of the target of empathy (i.e., assessing it towards patients is different from how it is assessed during parenting). Thus, dividing the samples was done to reduce heterogeneity. The present paper represents a sub-analysis on physicians and medical students, conducted within the parameters of the original protocol.

### Pairwise random-effects meta-analysis

Of the 112 independent samples extracted, 109 were included in the pairwise meta-analysis (three studies were excluded from this analysis as they lacked a control group). This analysis revealed an overall moderate effect size of *d* = 0.50 (95% CI = 0.40, 0.60) [46], where higher scores denoted higher empathy levels. Publication bias was evident (Egger’s test, *p* <.001). A visual inspection of the funnel plot indicated some evidence of asymmetry (Supplemental Appendix 8). Duval and Tweedie’s fill-and-trim procedure reported 15 missing studies to the right of the mean.

Since there was evidence of heterogeneity (*I*^2^ = 79.19, *p* <.001), a series of meta-regressions were run to test whether this could be explained by teaching methods, intervention characteristics, or study characteristics. The results of univariate meta-regressions are presented in Table 2. Interventions using didactic teaching methods were more effective (*d* = 0.61, 95% CI: 0.45, 0.78), on average, than those that did not use this method (*d* = 0.24, 95% CI:,12, 0.36). The number of teaching methods in an intervention was associated with effectiveness, such that interventions with two teaching methods (*d* = 0.96, 95% CI: 0.47, 1.45) performed better than those with three teaching methods (*d* = 0.36, 95% CI: 0.17, 0.40).

**Table 2:** Pairwise meta-analysis of studies comparing intervention to teach empathy vs no intervention: Random-effects meta-regression

	*k*	*SMD*	95% CI	*Q*	*p*	*R* ^2^
Instructional Components						
Didactic						
Yes	*88*	.59	.46, .72			
No	*21*	.24	.12, .36	6.33	.01	.02
Rehearsal						
Yes	*89*	.49	.38, .59			
No	*20*	.55	.24, .86	.05	.83	.00
Reflection						
Yes	*64*	.48	.36, .61			
No	*45*	.53	.36, .71	0.08	.78	.00
Observation						
Yes	*59*	.50	.38, .62			
No	*50*	.50	.34, .67	.08	.78	.00
Feedback						
Yes	*68*	.50	.39, .61			
No	*41*	.52	.33, .70	.04	.85	.02
Number of Teaching Methods						
1	*8*	.38	.12, .64			
2	*15*	.96	.47, 1.45			
3	*36*	.29	.17, .40			
4	*27*	.58	.41, .74			
5	*23*	.64	.43, .85	10.02	.04	.00
Intervention Characteristics						
Format						
Group	*62*	.66	.50, .82			
Group + Individual	*21*	.33	.18, .49			
Individual	*11*	.38	.09, .67			
Online/Independent	*15*	.21	.08, .34	8.72	.03	.00
Facilitator						
Professional	*71*	*.52*	.41, .65			
Researcher	*15*	*.54*	.28, .82			
Para-professional	*10*	*.63*	.01, 1.26			
Other	*13*	*.18*	.06, .30	4.80	.19	.00
Number of Sessions						
<5 Sessions	*74*	.48	.37, .59			
5-10 Sessions	*20*	.68	.31, 1.03			
10+	*7*	.38	.11, .66			
Not reported	*8*	.31	-.09, .70	2.61	.46	.00
Measurement Type						
Objective	*72*	.67	.52, .83			
Other-report	*23*	.24	.14, .34			
Self-report	*10*	.12	-.10, .33			
Mixed	*4*	.37	.13, .61	14.64	.00	.06
Study Characteristics						
Education Level						
Professional	*30*	.56	.36, .75			
Resident	*22*	.36	.17, .56			
Postgraduate	*33*	.72	.50, .95			
Undergraduate	*15*	.27	.09, .45			
Mixed	*9*	.42	.14, .71	7.39	.12	.00
Type of Control Group						
Pure Control	*47*	.71	.49, .93			
Active Control	*21*	.35	.19, .50			
Education As Usual	*41*	.37	.25, .49	6.65	.04	.01
Risk of Bias						
High	*60*	.52	.37, .67			
Low	*49*	.48	.34, .63	0.04	.84	.00

*k* = number of studies, *SMD* = standardized mean difference (Cohen’s *d*), *Q *= Cochrane’s Q

Interventions with in-person group formats (*d* = 0.66, 95% CI: 0.50, 0.82) were more effective than interventions with online/independent formats (*d* = 0.21, 95% CI: 0.08, 0.34). A statistically significant effect was found for measurement type with larger average effects on objective measures (*d* = 0.72, 95% CI: 0.51, 0.93) compared to self-report measures (*d* = 0.12, 95% CI: − 0.10, 0.34). Lastly, a statistically significant effect was found for control group type: larger average effects were found for studies which used pure control groups (i.e., no intervention; *d* = 0.71, 95% CI: 0.49, 0.93) versus studies which used education-as-usual groups (*d* = 0.37, 95% CI: 0.25, 0.49). No differences were found for other intervention characteristics (i.e., facilitator type, duration) or study characteristics (i.e., risk of bias, education level).

### Risk of bias assessment

We used the Cochrane risk-of-bias tool [37] to assess risk of bias for each of the included studies. Based on these criteria, a study was given a rating of *high*,* low*, or *unclear* risk for each of the seven Cochrane categories. Overall risk of bias judgement was determined as follows: “low risk” studies had four or more of the categories were determined to be *low risk.* “High risk” studies had four or more of the categories were determined to be *high* or *unclear risk*. Whether a study’s overall assessment is judged as “high risk” or “low risk” reflects the likelihood the results may be biased. In terms of overall risk of bias, there were “high risk of bias” concerns about risk of bias for over half the studies (55%, 60/109). However, no statistical difference was found in effect size between low- and high-risk studies (Table 2). See Supplemental Appendix 6 for summary of findings; *Additional File 2* for risk of bias justifications).

### Network meta-analysis

**Fig. 2:**
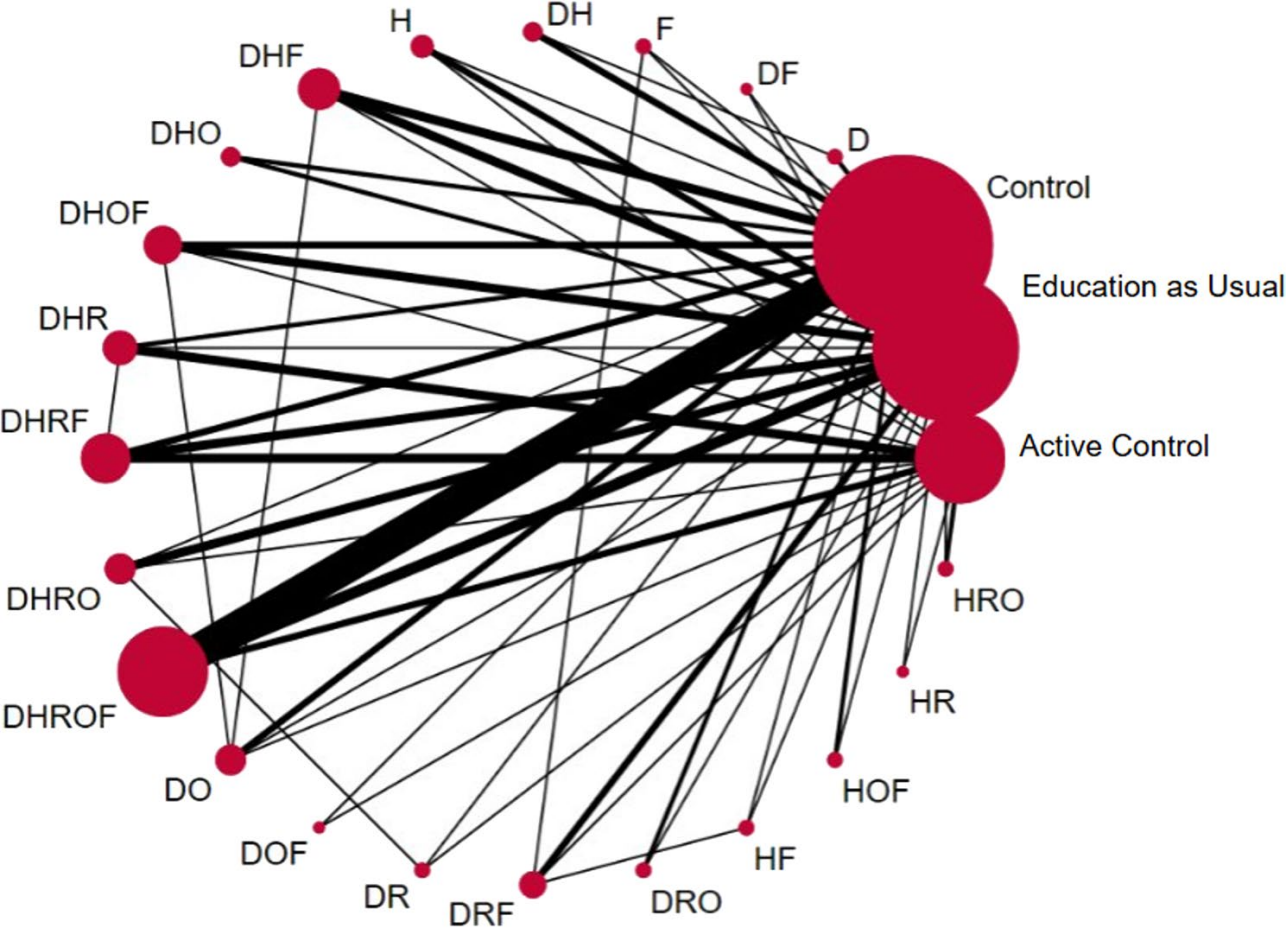
Network Meta-analysis of interventions to teach empathy: Network Geometry Note: The network geometry where each node represents an intervention (i.e., a combination of teaching methods). The size of the node corresponds to the number of studies with each program category. Interventions with direct comparisons are linked with a line (55 in total); the line width reflects the relative number of studies testing that specific comparison. Abbreviations: D =Didactic, H =Rehearsal, O = Observation, R =Reflection, F=Feedback

Figure 2 depicts the network geometry which shows the teaching method combinations and their relative frequency. The most common combination included all teaching methods (“DHROF”). The network meta-analysis included a wide range of multicomponent empathy training interventions (24 unique combinations of teaching methods, including three types of control groups). This resulted in 55 unique direct comparisons. Thicker lines indicate comparisons supported by more studies, while thinner lines reflect sparser evidence. The network was anchored around the control comparators, with many intervention nodes connected directly to Pure Control, forming a “hub-and-spoke” structure [47]. Several comparisons (e.g., DHROF vs. DHF, or DRF vs. DHRF) were informed primarily by indirect evidence, derived through shared comparators (e.g., DHROF vs. C vs. DHF). Others (e.g., DHROF vs. C, or DHRF vs. AC), had robust direct evidence, as shown by the number of contributing studies and thicker lines in the network diagram.

A global test of inconsistency [χ^2^ (33)= 39.11, *p* =.21] indicated no significant disagreement between direct and indirect estimates, supporting the assumption of coherence and the validity of indirect comparisons [43]. Visual symmetry and balance in the network support transitivity and connectedness [47]. Large control nodes (C, EAU, AC) have multiple direct comparisons, giving strong base anchors for indirect evidence, which are prerequisites for valid indirect evidence pooling [42]. Heterogeneity of variance was moderately high (τ^2^ = 0.68), however, visual inspection of the network forest plot indicated low levels of between-study heterogeneity (Supplemental Appendix 8).

**Fig. 3:**
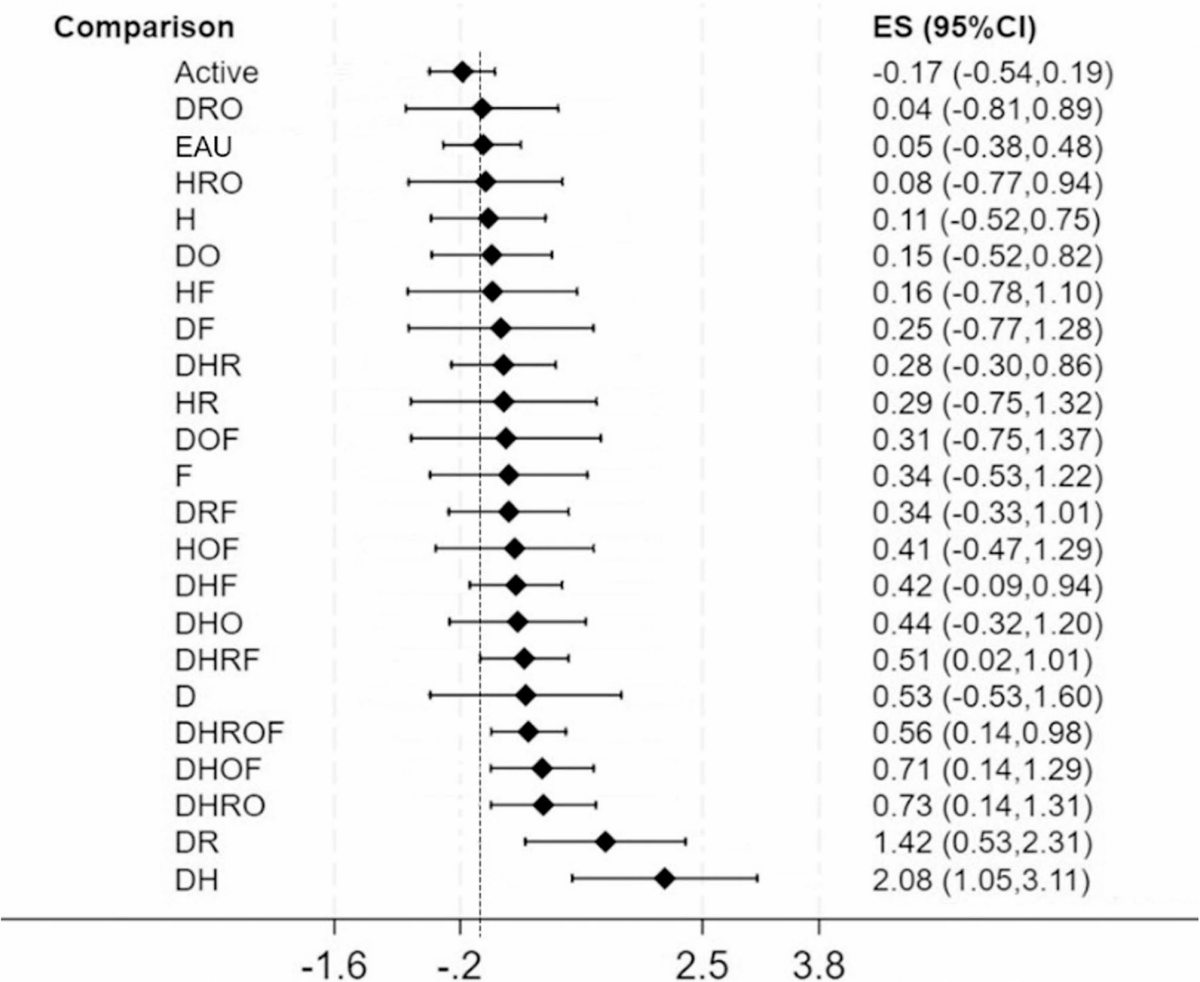
Network meta-analysis of interventions to teach empathy:Pooled effect sizes of each teaching method combination Note: A forest plot of all network comparisons in which each combination of teaching methods is compared to the pure control group (i.e., no intervention/waitlist). Abbreviations: AC = Active Control, EAU = Education as Usual, D =Didactic, H =Rehearsal, O = Observation, R =Reflection, F=Feedback, ES = effect size (standardized mean difference), CI = confidence intervals

Several combinations of teaching methods were statistically significantly more effective than the pure control group (see Fig. 3). These included the following combinations: didactic and rehearsal [DH, *d* = 2.08 (95% CI = 1.05, 3.11)]; didactic and reflection [DR, *d* = 1.42 (95% CI = 0.53, 2.31)]; didactic, rehearsal, reflection, and observation [DHRO, *d* = 0.73 (95% CI = 0.14, 1.31)]; didactic, rehearsal, observation, and feedback [DHOF, *d* = 0.71 (05% CI = 0.14, 1.29)]; all teaching methods [DHROF, *d* = 0.53 (95% CI = 0.14, 0.98)]; and didactic, rehearsal, reflection, and feedback [DHRF, *d* = 0.51 (95% CI = 0.02, 1.01)]. Of note, there are the wide confidence intervals for the top two teaching method combinations (DH and DR).

Relative efficacy ranking was conducted based on SUCRA curves, where higher percentages indicate a higher likelihood of being in a top rank. Results in Table 3 show that the top three combinations consisted of didactic and rehearsal (DH, 96.1%); didactic and reflection (DR, 86.2%); and didactic, rehearsal, reflection and observation (DHRO, 65.3%). For the SUCRA curves, see Supplemental Appendix 8. Results of the three sensitivity analyses were generally consistent (Supplemental Appendix 9), providing further support for the robustness of the findings.

**Table 3:** Surface under the cumulative ranking curve (SUCRA) results from the network meta-analysis

Treatment	SUCRA
DH	96.1
DR	86.2
DHRO	65.3
DHOF	64.9
DHROF	58.6
DHRF	56.8
D	55.8
DHO	53.1
HOF	51.8
DHF	51.2
DOF	47.9
F	47.1
DRF	46.9
DHR	46.8
HR	46
DF	44.8
HF	39.3
DO	38.7
H	38.5
HRO	35.8
DRO	35.2
Education as Usual	34.4
Control	32.8
Active Control	25.7

Higher percentages indicate the higher likelihood the teaching method combination is in the top rank

*Abbreviations*: *D* = Didactic, *H* = Rehearsal, *O* = Observation, *R* = Reflection, *F* = Feedback

### Assessment of risk of bias across studies

To assess small-study effects and potential publication bias across the network, we generated a comparison-adjusted funnel plot centered on the comparison-specific pooled effect sizes (Supplemental Appendix, Fig. 8.3). The distribution of studies appeared approximately symmetrical around the vertical reference line, with most effect sizes clustering near the center and within the expected funnel bounds. This pattern suggests no substantial evidence of small-study effects or reporting bias across the included comparisons. A small number of studies fell outside the funnel, particularly on the left side, indicating the presence of a few potential outliers; however, these were isolated and unlikely to meaningfully distort the overall network estimates (see Supplemental Appendix 9 for results of the sensitivity analysis where we removed outliers and removed grey literature).

### Assessment of certainty of evidence

A full description and summary of this assessment can be found in Supplemental Appendix 11. We assessed each of the five domains using qualitative judgments of low, moderate, or high concern. These concern levels reflect our appraisal of how much each domain might affect confidence in the findings. For *risk of bias* and *indirectness* domains, we did not consider this a serious limitation for PMA or NMA (low concern). For *inconsistency*, there high concern for the PMA, whereas for *precision*, there was low concern. For these two respective categories, the NMA showed moderate concern. For *publication bias*, both PMA and NMA showed moderate concern. Taken together, we judged the overall certainty in the evidence for both PMA and NMA to be moderate.

## Discussion

Previous empathy interventions for healthcare professionals have been shown to work with a moderately large effect size [16–18] and this was confirmed in this study. However, empathy interventions for physicians are often designed without knowing the most effective and efficient “ingredients” – what works, and what works well together?

### Which teaching methods are effective for teaching empathy to physicians?

The PMA found that including the didactic teaching method was associated with greater intervention effectiveness compared to control. This finding diverges from a previous meta-analysis on medical students, which found no one teaching method was better than another [18]. One reason may be that our study encompassed a greater number of studies (111 versus 18 studies), based on our inclusion of practicing physicians in addition to medical students. Pedagogically, didactic activities may be especially crucial for supporting skill acquisition in that it provides the theoretical structure to organize and assimilate newly acquired knowledge [48].

The reason the other teaching methods were not significant on their own may reflect the concept of *intentional alignment* from pedagogical research [49]. This is the notion that the teaching goal (i.e., desired outcome of the intervention) should align with the criteria of success (i.e., outcome measure) and with the learning activities (i.e., teaching methods). That is, didactic approaches may be particularly effective for targeting the aspect of empathy that is assessed on standardized measures used in research.

Moreover, it is possible that some teaching methods may be effective in some teaching contexts but not in others. For example, previous meta-analyses show considerable variability of feedback: some types of feedback can be powerful, whereas feedback that lacks specificity, timing alignment, and clarity is ineffective [50]. In fact, a seminal meta-analysis found approximately one-third of feedback interventions *decreased* performance [51]. From a practice perspective, this further highlights the importance of detailed reporting in studies. By providing in-depth description of the training itself, studies can be grouped more effectively; thus, with heterogeneity is reduced, meta-analytic conclusions become more robust.

### What intervention characteristics are important for empathy training?

In terms of number of teaching approaches, the PMA revealed that interventions using two teaching methods were more effective in increasing empathy than those with three. Although, it should be noted that there were only a small number of studies representing these combinations as (see relative line widths in Fig. 1; and Supplemental Appendix 12 for frequency of intervention combinations), and wide confidence intervals (Fig. 3), therefore replication is needed to confirm this finding. These results are aligned with other meta-analyses on empathy interventions for mental health professionals and parents [32, 52], which shows that focused interventions can be very impactful for teaching empathy skills. However, these results are somewhat surprising in light of previous literature for medical training, which shows the effectiveness of simulation-based education of using at least three teaching methods [53] (i.e., didactic, rehearsal, and feedback). Instead, as we describe in the NMA section below, it seems that certain *combinations* of teaching methods can be especially effective when paired together.

The PMA also showed which intervention characteristics may be more or less important for teaching empathy: format was statistically significant but not the number of sessions or facilitator type. In-person group formats were more effective than online and/or independent formats, which are in line with research showing that peer collaboration may be particularly beneficial for acquiring an interpersonal skill such as empathy [54] as group settings may be uniquely conducive for acquiring social and self knowledge [55]. The non-finding of session number is in line with previous studies which did not find a significant effect of duration as a moderator of intervention effect [18, 24, 25]. Again, underreporting intervention details makes it hard to study the impact of specific intervention characteristics. For example, while the duration of the empathy interventions overall was not associated with effectiveness in our study, duration of specific teaching methods could be, though this is rarely reported by studies.

The type of facilitator did not impact intervention effectiveness, suggesting that empathy skills need not be taught only by professionals, as those with comparatively fewer credentials can be as effective educators for empathy skills [56]. Additionally, learning outcomes may be boosted with a focus on *learner experience*, such as motivation, engagement, and satisfaction [57, 58]. In pedagogical research, there is strong meta-analytic evidence showing that positive teacher behaviours (e.g., warmth, non-judgement, and genuineness) are significantly associated with improved learning outcomes [59]. In medical education, recent systematic reviews also highlight how clinical supervisors are a key influence of student empathy development, where exposure to emotionally distant or “efficiency driven” role models may contribute to the overall decline of empathy during medical school [60, 61]. Student self-report (although not a blinded outcome in trial design) may serve an important function for understanding educational outcomes [49].

### Which study characteristics impact the effectiveness of empathy training?

Study characteristics which were associated with intervention effectiveness included measurement type and control group type. In line with previous meta-analyses [25], larger effects were seen on objective measures as compared to patient-report and self-reports of empathy. It may be that self-reports have less reliable psychometric properties and more prone to bias, which could negatively impact its measure of empathy [62]. Moreover, it is possible that interventions demonstrated stronger effects on objective measures of empathy because these assessments closely mirror the structure and content of the training itself. This may reflect a form of “teaching to the test” [63], where participants are prepared to perform well on behaviors they know will be evaluated. As such, objective measures may potentially overestimate the real-world impact of the intervention compared to patient-reported outcomes, which are more sensitive to the nuanced and relational aspects of empathy [64, 65].

We also found a greater effect size when comparing studies which used a pure control group over those which used education-as-usual. While this was not found in previous meta-analyses on empathy interventions [18, 25], this inconsistency may be in part due to a lack of precision when it comes to defining control groups; this is especially the case when it comes to behavioural interventions [66]. Overall, effect sizes for moderator analyses were small in magnitude, a finding that is common in PMAs and indicative of high heterogeneity between studies. This may reflect the diversity of behavioural interventions that exist to target a skill as complex as empathy; it will be important for future studies to further explore this heterogeneity.

### What combinations of teaching methods are effective?

NMA rankings revealed the most effective empathy intervention was comprised of didactic and rehearsal methods, followed by didactic and reflection. The third, fourth and fifth ranks were 3) didactic, rehearsal, reflection and observation; 4) didactic, rehearsal, observation, and feedback; and 5) all five teaching methods together. The most common teaching methods in these combinations included didactic (5/5) followed by rehearsal (4/5), reflection and observation (3/5). These findings align with results from previous meta-analyses that suggest a mix of teaching methods is effective for teaching empathy, the importance of didactic teaching, and a trend towards the benefit of rehearsal methods [18, 23].

These results also show that certain combinations of teaching methods are more effective than combining all methods together (i.e., DHROF). Consistent with the PMA, the two top combinations of the NMA involve only two teaching methods. Notably, while the highest performing interventions comprised only two teaching methods, the effects of interventions with more methods were more robust (i.e., yielded smaller confidence intervals). By examining the SUCRA rankings, it is possible to see that the combinations of teaching methods that show statistically significant effect sizes (see Fig. 3) include at least one “surface learning” component (mastery of content) and at least one “deep learning” component (connections between, and extension of, the content) [21], which generally achieved through a component that is experiential (see Table 1). Specifically, the combination of didactic and “experiential” (rehearsal and reflection) teaching methods were the most effective intervention combinations.

Didactic teaching may be necessary to give physicians the cognitive framework upon which they can build the concepts as they learn; however, this teaching method may not be the most effective alone. Instead, didactic may work especially well when combined with rehearsal or reflection activities; this provides for the opportunity for application and consolidation of empathy skills, which is key to learning [21, 67]. Acquiring a high level of competency of procedural skills in medicine requires a similar pedagogical approach, which first requires acquiring cognitive knowledge of a skill, following adequate exposure and practice, before being able to expertly performing the skill [68].

Furthermore, these NMA results also highlight practice gaps between what is commonly done (i.e., frequency of intervention combinations) compared to what is most impactful (i.e., using effective intervention combinations). For example, feedback is often identified as an active ingredient in medical education literature [53, 69], so educators may feel that it is an essential component for training *all* types of patient physician communication behaviors; that may explain why four out of five of the most frequent interventions contain feedback. What our study suggests, however, is that it may not be as “essential” as presumed; in fact, three of the five significant intervention combinations do not include feedback (i.e., DH, DR, DHRO). It is possible that what is effective may vary for the type of communication behavior being taught. Notably, a teaching method like feedback is an expensive component for teaching empathy, and if it is possible to achieve the same empathy outcome without it, this may be more cost-effective. Therefore, our study shows that NMA can be an insightful tool for identifying effective interventions, thus providing some direction of what key ingredients program developers and educators should focus on, rather than solely relying on what is common practice.

### Limitations & future directions

Results should be interpreted with the following limitations in mind. First, publication bias was detected, which is not uncommon in PMA and NMA [70], and could inflate the magnitude of the observed findings [71]. Nonetheless, this speaks to the need for future work to emphasize pre-registration and publication of null findings. There is especially need for additional research on how best to address publication bias, especially in large NMAs [72]. Second, heterogeneity is a known problem in NMAs involving behavioral and educational interventions [43, 73, 74]. This could be addressed by standardizing interventions, methods and reporting [75, 76]. Following the CONSORT-SPI tool [77] and providing better documentation in original studies (including details of sampling, intervention content and delivery method) are crucial to improving aggregations of evidence.

Third, when studies reported multiple types of empathy measures, we prioritized objective measures over patient-reported or self-report outcomes because objective measures allowed for greater cross-study comparability and are less susceptible to expectancy effects [78]. We recognize, however, patient-perceived empathy is a critical dimension, given its association to patient outcomes [79]. Moreover, excluding patient-reported measures may limit insights into how these interventions impact patients’ actual experiences. For example, one study has demonstrated that the impact of physician empathy on patients’ emotional quality of life depends upon the type of consultation and the patient’s emotional skills [65]. Patient-perceived empathy can provide deeper understanding of the impact of physician empathy. Therefore, it will be crucial next step to assess the ecological validity of empathy interventions with real patients as well.

Fourth, a great majority of the studies reviewed were from either two regions, Europe and Central Asia or North America (80%, 87/109), which may not capture the diverse ways in which empathy training is approached. It would be ideal for future work to investigate how different cultural and linguistic contexts may change the teaching and measurement of empathy [80]. Fifth, the literature search for this review was conducted approximately three years prior to manuscript submission (October 2022) and therefore does not include the most recent studies on empathy interventions. Updating the search to incorporate newer evidence would be a valuable direction. Lastly, while the current study did not find an impact of intervention duration, most studies did not examine long term follow-up to see how long intervention effects last. Future studies should focus on booster sessions to determine whether this could mitigate empathy decline [68, 81].

## Conclusion

We add evidence to support data-driven decision-making in the design of medical education programs. This study is the first to systematically examine a large sample of randomized controlled trials of empathy interventions for physicians across all training levels, increasing the generalizability of our results from medical students to those in practice. Our novel contributions also include the value of didactic teaching and in-person group formats. A methodological strength includes the use of NMA, which allowed for the identification of the effective combinations of teaching methods, such as the combination of didactic with rehearsal teaching methods. Practicing effective medicine includes practicing empathy, making the optimization of empathy interventions an ongoing scientific and public health endeavor. 

## Supplementary Information

Additional File 1.

Additional File 2.

Supplementary Appendices.

## Data Availability

The datasets used and/or analysed during the current study are available from the corresponding author on reasonable request. Analytic code is available in Supplemental Appendix 2.
