# Supplementary material for: A systematic review identifying effective teaching methods and their combinations for increasing empathy in physicians: pairwise and network meta-analysis
Source: BMC Med Educ. 2025 Oct 23;25:1483. doi: 10.1186/s12909-025-07917-x (PMC12548198; doi:10.1186/s12909-025-07917-x)
Supplement: Supplementary file 3 — Supplementary Appendices. [file 12909_2025_7917_MOESM3_ESM.docx]

# Supplemental Appendices

# Supplemental Appendix 1: Search Strategy

# Supplemental Appendix 2: Network Meta-Analysis Code (STATA SE)

# Supplemental Appendix 3: Study References

# Supplemental Appendix 4: Definitions of Study Variables

# Supplemental Appendix 5: Percentages of Study and Participant Characteristics

# Supplemental Appendix 6: Individual Study Information Tables

# Supplemental Appendix 7: Publication Bias Testing Funnel Plot

# Supplemental Appendix 8: Network Meta-Analysis Figures

# Supplemental Appendix 9: Network Meta-Analysis Sensitivity Analyses

# Supplemental Appendix 10: Table 3 references

# Supplemental Appendix 11: Certainty of Evidence Assessment

# Supplemental Appendix 12: Frequency of Intervention Combinations

Supplemental Appendix 13: Risk of Bias Coding Criteria

# Supplemental Appendix 1

# Search Strategy

**OVID: PSYCINFO**

1. empathy/

2. empath*.mp.

3. mentalization/

4. mentaliz*.mp.

5. mentalis*.mp.

6. role taking/

7. (tak* adj3 perspective).mp.

8. attune*.mp.

9. reflective function*.mp.

10. active listening/

11. listening (interpersonal)/

12. active listen*.mp.

13. narrative competence.mp.

14. other orient*.mp.

15. mind?read*.mp.

16. mindsight.mp.

17. mind minded*.mp.

18. theory of mind.mp.

19. Emotional intelligence/

20. (emotion* adj (intelligen* or competen*)).mp.

21. (social* adj (intelligen* or competen* or perceptiv*)).mp.

22. (interpersonal* adj (intelligen* or competen* or skill* or communicat*)).mp.

23. Interpersonal communication/

24. communication skills.mp.

25. ((responsiv* or sensitiv* or synchron*) adj3 (parent* or caregiv* or mother* or father* or

matern* or patern* or couple* or partner* or wife* or husband* or marital* or teach* or

educat* or physician* or doctor* or surgeon* or dentist* or GP* or resident* or nurse* or

practitioner* or clinician* or therapist* or social worker* or psychologist* or psychiatrist* or

coach* or manager* or employee* or professional*)).mp.

26. (understand* adj2 (other* or another*)).mp.

27. or/1-26

28. randomi*.mp.

29. (random* adj3 (trial* or stud* or assign* or allocat* or design)).mp.

30. rct.mp.

31. (trial adj3 (control* or clinical)).mp.

32. wait?list*.mp.

33. wait* list*.mp

34. placebo*.mp.

35. ((singl* or doubl* or trebl* or tripl*) adj3 (blind* or mask*)).mp.

36. or/28-35

37. 27 and 36

38. Limit 37 to (human and English language)

**OVID: MED-LINE**

1. empathy/

2. empath*.mp.

3. mentaliz*.mp.

4. mentalis*.mp.

5. (tak* adj3 perspective).mp.

6. attune*.mp.

7. reflective function*.mp.

8. active listen*.mp.

9. narrative competence.mp.

10. other orient*.mp.

11. mind?read*.mp.

12. mindsight.mp.

13. mind minded*.mp.

14. theory of mind.mp.

15. emotional intelligence/

16. (emotion* adj2 (intelligence or competence)).mp.

17. (social adj2 (intelligence or competence or perceptiveness)).mp.

18. (interpersonal adj2 (intelligence or competence or skills or communication)).mp.

19. communication skills.mp.

20. ((responsiv* or sensitiv* or synchron*) adj3 (parent* or caregiv* or mother* or father*

or matern* or patern* or couple* or partner* or wife* or husband* or marital* or

teach* or educat* or physician* or doctor* or surgeon* or dentist* or GP* or resident*

or nurse* or practitioner* or clinician* or therapist* or social worker* or psychologist*

or psychiatrist* or coach* or manager* or employee* or professional*)).mp.

21. (understand* adj2 (other* or another*)).mp.

22. or/1-21

23. randomized controlled trial/

24. randomi*.mp.

25. (random* adj3 (trial* or stud* or assign* or allocat* or design)).mp.

26. rct.mp.

27. (trial adj3 (control* or clinical)).mp.

28. wait?list*.mp.

29. wait* list*.mp

30. placebo*.mp.

31. ((singl* or doubl* or trebl* or tripl*) adj3 (blind* or mask*)).mp.

32. or/23-31

33. 22 and 32

34. Limit 33 to (human and English language)

**OVID: SOCIAL WORK ABSTRACTS**

1. empath*.mp.

2. mentaliz*.mp.

3. mentalis*.mp.

4. (tak* adj3 perspective).mp.

5. attune*.mp.

6. reflective function*.mp.

7. active listen*.mp.

8. narrative competence.mp.

9. other orient*.mp.

10. mind?read*.mp.

11. mindsight.mp.

12. mind minded*.mp.

13. theory of mind.mp.

14. (emotion* adj2 (intelligence or competence)).mp.

15. (social adj2 (intelligence or competence or perceptiveness)).mp.

16. (interpersonal adj2 (intelligence or competence or skills or communication)).mp.

17. communication skills.mp.

18. ((responsiv* or sensitiv* or synchron*) adj3 (parent* or caregiv* or mother* or father*

or matern* or patern* or couple* or partner* or wife* or husband* or marital* or

teach* or educat* or physician* or doctor* or surgeon* or dentist* or GP* or resident*

or nurse* or practitioner* or clinician* or therapist* or social worker* or psychologist*

or psychiatrist* or coach* or manager* or employee* or professional*)).mp.

19. (understand* adj2 (other* or another*)).mp.

20. or/1-19

21. randomi*.mp.

22. (random* adj3 (trial* or stud* or assign* or allocat* or design)).mp.

23. rct.mp.

24. (trial adj3 (control* or clinical)).mp.

25. wait?list*.mp.

26. wait* list*.mp

27. placebo*.mp.

28. ((singl* or doubl* or trebl* or tripl*) adj3 (blind* or mask*)).mp.

29. or/21-18

30. 20 and 29

**EBSCO: CINAHL**

S1. empath*

S2. mentaliz*

S3. mentalis*

S4. tak* N3 perspective

S5. attune*

S6. “reflective function*”

S7. “active listen*”

S8. “narrative competence”

S9. “other orient*”

S10. mindread* or “mind read*”

S11. mindsight

S12. “mind minded*”

S13. “theory of mind”

S14. emotion* W0 (intelligen* or competen*)

S15. social* W0 (intelligen* or competen* or perceptiv*)

S16. interpersonal* W0 (intelligen* or competen* or skill* or communicat*)

S17. “communication skills”

S18. (responsiv* or sensitiv* or synchron*) N3 (parent* or caregiv* or mother* or father*

or matern* or patern* or couple* or partner* or wife* or husband* or marital* or teach*

or educat* or physician* or doctor* or surgeon* or dentist* or GP* or resident* or nurse*

or practitioner* or clinician* or therapist* or “social worker*” or psychologist* or

psychiatrist* or coach* or manager* or employee* or professional*)

S19. understand* N2 (other* or another*)

S20. S1 OR S2 OR S3 OR S4 OR S5 OR S6 OR S7 OR S8 OR S9 OR S10 OR S11 OR S12 OR S13

OR S14 OR S15 OR S16 OR S17 OR S18 OR S19

S21. randomi*

S22. random* N3 (trial* or stud* or assign* or allocat* or design)

S23. rct

S24. trial N3 (control* or clinical)

S25. waitlist* or “wait* list*”

S27. placebo*

S28. (singl* or doubl* or trebl* or tripl*) N3 (blind* or mask*)

S29. S21 OR S22 OR S23 S25 OR S26 OR S27 OR S28

S30. S20 and S29

Narrow by language: English

Search: Title, Abstract, MW (Word in Subject Heading)

**PROQUEST: ERIC**

noft(((responsiv* OR sensitiv* OR synchron*) NEAR/3 (parent* OR caregiv* OR mother* OR

father* OR matern* OR patern* OR couple* OR partner* OR wife* OR husband* OR marital*

OR teach* OR educat* OR physician* OR doctor* OR surgeon* OR dentist* OR GP* OR

resident* OR nurse* OR practitioner* OR clinician* OR therapist* OR “social worker*” OR

psychologist* OR psychiatrist* OR coach* OR manager* OR employee* OR professional*)) OR

empath* OR mentaliz* OR mentalis* OR (tak* NEAR/3 perspective) OR attune* OR "reflective

function*" OR "active listen*" OR "narrative competence" OR "other orient*" OR mindread* OR

“mind read*” OR mindsight OR “mind minded*” OR “theory of mind” OR (emotion* PRE/0

(intelligen* OR competen*)) OR (social* PRE/0 (intelligen* OR competen* OR perceptiv*)) OR

(interpersonal* PRE/0 (intelligen* OR competen* OR skill* OR communicat*)) OR

“communication skills” OR (understand* NEAR/2 (other* or another*))) AND noft(randomi* OR

(random* NEAR/3 (trial* OR stud* OR assign* OR allocat* OR design)) OR rct OR (trial N3

(control* OR clinical)) OR waitlist* OR “wait* list*” OR placebo* OR ((singl* OR doubl* OR

trebl* OR tripl*) NEAR/3 (blind* OR mask*))) AND la.exact(“ENG”)

**PROQUEST: ABI/INFORM**

noft(((responsiv* OR sensitiv* OR synchron*) NEAR/3 (parent* OR caregiv* OR mother* OR

father* OR matern* OR patern* OR couple* OR partner* OR wife* OR husband* OR marital*

OR teach* OR educat* OR physician* OR doctor* OR surgeon* OR dentist* OR GP* OR

resident* OR nurse* OR practitioner* OR clinician* OR therapist* OR “social worker*” OR

psychologist* OR psychiatrist* OR coach* OR manager* OR employee* OR professional*)) OR

empath* OR mentaliz* OR mentalis* OR (tak* NEAR/3 perspective) OR attune* OR "reflective

function*" OR "active listen*" OR "narrative competence" OR "other orient*" OR mindread* OR

“mind read*” OR mindsight OR “mind minded*” OR “theory of mind” OR (emotion* NEAR/2

(intelligence OR competence)) OR (social NEAR/2 (intelligence OR competence OR

perceptiveness)) OR (interpersonal NEAR/2 (intelligence OR competence OR skills OR

communication)) OR “communication skills” OR (understand* NEAR/2 other*)) AND

noft(randomi* OR (random* NEAR/3 (trial* OR stud* OR assign* OR allocat* OR design)) OR rct

OR (trial N3 (control* OR clinical)) OR waitlist* OR “wait* list*” OR placebo* OR ((singl* OR

doubl* OR trebl* OR tripl*) NEAR/3 (blind* OR mask*))) AND la.exact(“ENG”)

**COCHRANE: CENTRAL**

#1 MeSH descriptor: [Empathy] explode all trees

#2 empath*:ti,ab,kw

#3 mentaliz*:ti,ab,kw

#4 mentalis*:ti,ab,kw

#5 (tak* near/3 perspective):ti,ab,kw

#6 attune*:ti,ab,kw

#7 “reflective function*”:ti,ab,kw

#8 “active listen*”:ti,ab,kw

#9 “narrative competence”:ti,ab,kw

#10 “other orient*”:ti,ab,kw

#11 (mindread or “mind read*”):ti,ab,kw

#12 mindsight:ti,ab,kw

#13 “mind minded*”:ti,ab,kw

#14 “theory of mind”:ti,ab,kw

#15 MeSH descriptor: [Emotional intelligence] this term only

#16 (emotion* next (intelligen* or competen*)):ti,ab,kw

#17 (social* next (intelligen* or competen* or perceptiv*)):ti,ab,kw

#18 (interpersonal* next (intelligen* or competen* or skill* or communicat*)):ti,ab,kw

#19 “communication skills”:ti,ab,kw

#20 ((responsiv* or sensitiv* or synchron*) near/3 (parent* or caregiv* or mother* or father*

or matern* or patern* or couple* or partner* or wife* or husband* or marital* or teach* or

educat* or physician* or doctor* or surgeon* or dentist* or GP* or resident* or nurse* or

practitioner* or clinician* or therapist* or social worker* or psychologist* or psychiatrist* or

coach* or manager* or employee* or professional*)):ti,ab,kw

#21 (understand* near/2 (other* or another*)):ti,ab,kw

#22 (#1 or #2 or #3 or #4 or #5 or #6 or #7 or #8 or #9 or #10 or #11 or #12 or #13 or #14 or #15

or #16 or #17 or #18 or #19 or #20 or #21)

#23 #22 in Trials

# Supplemental Appendix 2

# Network Meta-Analysis Code (STATA SE)

foreach var of varlist t1 t2 {

replace `var'="A" if `var'=="AC"

replace `var'="B" if `var'=="SC"

replace `var'="C" if `var'=="C"

replace `var'="D" if `var'=="D"

replace `var'="E" if `var'=="DF"

replace `var'="F" if `var'=="F"

replace `var'="G" if `var'=="DH"

replace `var'="H" if `var'=="H"

replace `var'="I" if `var'=="DHF"

replace `var'="J" if `var'=="DHO"

replace `var'="K" if `var'=="DHOF"

replace `var'="L" if `var'=="DHR"

replace `var'="M" if `var'=="DHRF"

replace `var'="N" if `var'=="DHRO"

replace `var'="O" if `var'=="DHROF"

replace `var'="P" if `var'=="DO"

replace `var'="Q" if `var'=="DOF"

replace `var'="R" if `var'=="DR"

replace `var'="S" if `var'=="DRF"

replace `var'="T" if `var'=="DRO"

replace `var'="U" if `var'=="HF"

replace `var'="V" if `var'=="HOF"

replace `var'="W" if `var'=="HR"

replace `var'="X" if `var'=="HRO"

}

*************help & packages************

help network graphs

*network_graphs

net from http://www.clinicalepidemio.fr/Stata

net from http://www.clinicalepidemio.fr/stat

*mvmeta package

net from http://www.homepages.ucl.ac.uk/~rmjwiww/stata

*****************************************

network import, studyvar(studyid) treat (t2 t1) effect(ef) stderr(se)

*network geometry

networkplot t2 t1, labels("Active Control" "Standard Control" "Control" "D" "DF" "F" "DH" "H" "DHF" "DHO" "DHOF" "DHR" "DHRF" "DHRO" "DHROF" "DO" "DOF" "DR" "DRF" "DRO" "HF" "HOF" "HR" "HRO")nodecolor(cranberry)

network convert augmented, ref("C")

*network consistency model

network meta c

*tests inconsistency; want no sig p values

network meta i

*tests local inconsistency

network sidesplit all, tau

intervalplot, pred ref("Control") labels("Active Control" "Standard Control" "Control" "D" "DF" "F" "DH" "H" "DHF" "DHO" "DHOF" "DHR" "DHRF" "DHRO" "DHROF" "DO" "DOF" "DR" "DRF" "DRO" "HF" "HOF" "HR" "HRO")

*all effect sizes

network forest, group(design)xline(0, lc(gs14)) columns (xtile) colors(dknavy cranberry ltblue) msize(*.08)diamond

*SUCRA ranking

help network rank

network rank max, zero all gen(prob) predict reps(1000)

search sencode

*SUCRA graphs

sucra prob*, labels("Active Control" "Standard Control" "Control" "D" "DF" "F" "DH" "H" "DHF" "DHO" "DHOF" "DHR" "DHRF" "DHRO" "DHROF" "DO" "DOF" "DR" "DRF" "DRO" "HF" "HOF" "HR" "HRO")

# Supplemental Appendix 3

# Study References

1. Abbasi S, Rakhshani T, Rezaie M, ebrahimi M R, Taravatmanesh S. A study of emotional intelligence and the effect of educational intervention in emergency medicine residents. Archives of Psychiatry and Psychotherapy. 2018;20(1):45-52. doi:10.12740/APP/82317.
2. Alder J, Christen R, Zemp E, Bitzer J. Communication skills training in obstetrics and gynaecology: whom should we train? A randomized controlled trial. *Arch Gynecol Obstet*. 2007;276(6):605-612. doi:10.1007/s00404-007-0399-0
3. Annadurai V, Smith CB, Bickell N, et al. Impact of a Novel Goals-of-Care Communication Skills Coaching Intervention for Practicing Oncologists. *J Palliat Med*. 2021;24(6):838-845. doi:10.1089/jpm.2020.0207
4. Bashour HN, Kanaan M, Kharouf MH, Abdulsalam AA, Tabbaa MA, Cheikha SA. The effect of training doctors in communication skills on women's satisfaction with doctor-woman relationship during labour and delivery: a stepped wedge cluster randomised trial in Damascus. *BMJ Open*. 2013;3(8):e002674. Published 2013 Aug 14. doi:10.1136/bmjopen-2013-002674
5. Beach MC, Roter DL, Saha S, et al. Impact of a brief patient and provider intervention to improve the quality of communication about medication adherence among HIV patients. *Patient Educ Couns*. 2015;98(9):1078-1083. doi:10.1016/j.pec.2015.05.011
6. Bearman M, Cesnik B, Liddell M. Random comparison of 'virtual patient' models in the context of teaching clinical communication skills. *Med Educ*. 2001;35(9):824-832. doi:10.1046/j.1365-2923.2001.00999.x
7. Bibl K, Wagner M, Steinbauer P, et al. NeoAct: A Randomized Prospective Pilot Study on Communication Skill Training of Neonatologists. *Front Pediatr*. 2021;9:675742. Published 2021 May 13. doi:10.3389/fped.2021.675742
8. Bickell NA, Back AL, Adelson K, et al. Effects of a Communication Intervention Randomized Controlled Trial to Enable Goals-of-Care Discussions. *JCO Oncol Pract*. 2020;16(9):e1015-e1028. doi:10.1200/OP.20.00040
9. **Blatt B, LeLacheur SF, Galinsky AD, Simmens SJ, Greenberg L. Does Perspective-Taking Increase Patient Satisfaction in Medical Encounters? *Academic Medicine*. 2010;85(9):1445. doi:[10.1097/ACM.0b013e3181eae5ec](https://doi.org/10.1097/ACM.0b013e3181eae5ec)
10. Blödt S, Mittring N, Schützler L, et al. A consultation training program for physicians for communication about complementary medicine with breast cancer patients: a prospective, multi-center, cluster-randomized, mixed-method pilot study. *BMC Cancer*. 2016;16(1):843. Published 2016 Nov 4. doi:10.1186/s12885-016-2884-y
11. *Bonvicini KA, Perlin MJ, Bylund CL, Carroll G, Rouse RA, Goldstein MG. Impact of communication training on physician expression of empathy in patient encounters. *Patient Educ Couns*. 2009;75(1):3-10. doi:10.1016/j.pec.2008.09.007
12. Bosse HM, Schultz JH, Nickel M, et al. The effect of using standardized patients or peer role play on ratings of undergraduate communication training: a randomized controlled trial. *Patient Educ Couns*. 2012;87(3):300-306. doi:10.1016/j.pec.2011.10.007
13. Bowyer MW, Hanson JL, Pimentel EA, et al. Teaching breaking bad news using mixed reality simulation. *J Surg Res*. 2010;159(1):462-467. doi:10.1016/j.jss.2009.04.032
14. Brinkman WB, Geraghty SR, Lanphear BP, et al. Effect of multisource feedback on resident communication skills and professionalism: a randomized controlled trial. *Arch Pediatr Adolesc Med*. 2007;161(1):44-49. doi:10.1001/archpedi.161.1.44
15. Brown CE, Back AL, Ford DW, et al. Self-Assessment Scores Improve After Simulation-Based Palliative Care Communication Skill Workshops. *Am J Hosp Palliat Care*. 2018;35(1):45-51. doi:10.1177/1049909116681972
16. Brown JB, Boles M, Mullooly JP, Levinson W. Effect of clinician communication skills training on patient satisfaction. A randomized, controlled trial. *Ann Intern Med*. 1999;131(11):822-829. doi:10.7326/0003-4819-131-11-199912070-00004
17. Buffel du Vaure C, Lemogne C, Bunge L, et al. Promoting empathy among medical students: A two-site randomized controlled study. *Journal of Psychosomatic Research*. 2017;103:102-107. doi:[10.1016/j.jpsychores.2017.10.008](https://doi.org/10.1016/j.jpsychores.2017.10.008)
18. Butow P, Brown R, Aldridge J, et al. Can consultation skills training change doctors' behaviour to increase involvement of patients in making decisions about standard treatment and clinical trials: a randomized controlled trial. *Health Expect*. 2015;18(6):2570-2583. doi:10.1111/hex.12229
19. Butow P, Cockburn J, Girgis A, et al. Increasing oncologists' skills in eliciting and responding to emotional cues: evaluation of a communication skills training program. *Psychooncology*. 2008;17(3):209-218. doi:10.1002/pon.1217
20. Cave J, Washer P, Sampson P, Griffin M, Noble L. Explicitly linking teaching and assessment of communication skills. *Med Teach*. 2007;29(4):317-322. doi:10.1080/01421590701509654
21. Cooper LA, Roter DL, Carson KA, et al. A Randomized Trial to Improve Patient-Centered Care and Hypertension Control in Underserved Primary Care Patients. *J Gen Intern Med*. 2011;26(11):1297-1304. doi:[10.1007/s11606-011-1794-6](https://doi.org/10.1007/s11606-011-1794-6)
22. Coret A, Boyd K, Hobbs K, Zazulak J, McConnell M. Patient Narratives as a Teaching Tool: A Pilot Study of First-Year Medical Students and Patient Educators Affected by Intellectual/Developmental Disabilities. *Teach Learn Med*. 2018;30(3):317-327. doi:10.1080/10401334.2017.1398653
23. D'souza PC, Rasquinha SL, D'souza TL, Jain A, Kulkarni V, Pai K. Effect of a Single-Session Communication Skills Training on Empathy in Medical Students. *Acad Psychiatry*. 2020;44(3):289-294. doi:10.1007/s40596-019-01158-z
24. Daeppen JB, Fortini C, Bertholet N, et al. Training medical students to conduct motivational interviewing: a randomized controlled trial. *Patient Educ Couns*. 2012;87(3):313-318. doi:10.1016/j.pec.2011.12.005
25. Daetwyler C, Cohen D, Gracely E, Novack D. eLearning to Enhance physician patient communication: A pilot test of “doc.com” and “WebEncounter” in teaching bad news delivery. *Medical teacher*. 2010;32:e381-90. doi:[10.3109/0142159X.2010.495759](https://doi.org/10.3109/0142159X.2010.495759)
26. DeBlasio D, Real FJ, Ollberding NJ, Klein MD. Provision of Parent Feedback via the Communication Assessment Tool: Does It Improve Resident Communication Skills?. *Acad Pediatr*. 2019;19(2):152-156. doi:10.1016/j.acap.2018.06.013
27. Downar J, McNaughton N, Abdelhalim T, et al. Standardized patient simulation versus didactic teaching alone for improving residents' communication skills when discussing goals of care and resuscitation: A randomized controlled trial. *Palliat Med*. 2017;31(2):130-139. doi:10.1177/0269216316652278
28. Dubosh NM, Hall MM, Novack V, Shafat T, Shapiro NI, Ullman EA. A Multimodal Curriculum With Patient Feedback to Improve Medical Student Communication: Pilot Study. *West J Emerg Med*. 2019;21(1):115-121. Published 2019 Dec 9. doi:10.5811/westjem.2018.11.44318
29. Duran A, Donelan C, Bowman Peterson J, Gladding SP, Weissmann P, Roth CS. Communicating value to patients-a high-value care communication skills curriculum. *Postgrad Med*. 2021;133(2):231-236. doi:10.1080/00325481.2020.1807728
30. Dwyer CP, MacNeela P, Durand H, et al. Effects of Biopsychosocial Education on the Clinical Judgments of Medical Students and GP Trainees Regarding Future Risk of Disability in Chronic Lower Back Pain: A Randomized Control Trial. *Pain Med*. 2020;21(5):939-950. doi:10.1093/pm/pnz284
31. Epinat-Duclos J, Foncelle A, Quesque F, et al. Does nonviolent communication education improve empathy in French medical students?. *Int J Med Educ*. 2021;12:205-218. doi:10.5116/ijme.615e.c507
32. Farrell MH, Christopher SA, La Pean Kirschner A, et al. Improving the quality of physician communication with rapid-throughput analysis and report cards. *Patient Educ Couns*. 2014;97(2):248-255. doi:10.1016/j.pec.2014.07.028
33. Flocke SA, Step MM, Antognoli E, et al. A randomized trial to evaluate primary care clinician training to use the Teachable Moment Communication Process for smoking cessation counseling. *Prev Med*. 2014;69:267-273. doi:10.1016/j.ypmed.2014.10.020
34. Foster A, Chaudhary N, Kim T, et al. Using Virtual Patients to Teach Empathy: A Randomized Controlled Study to Enhance Medical Students’ Empathic Communication. *Simulation in Healthcare*. 2016;11(3):181. doi:[10.1097/SIH.0000000000000142](https://doi.org/10.1097/SIH.0000000000000142)
35. Foster A, Chaudhary N, Murphy J, Lok B, Waller J, Buckley PF. The Use of Simulation to Teach Suicide Risk Assessment to Health Profession Trainees-Rationale, Methodology, and a Proof of Concept Demonstration with a Virtual Patient. *Acad Psychiatry*. 2015;39(6):620-629. doi:10.1007/s40596-014-0185-9
36. Fujimori M, Shirai Y, Asai M, Kubota K, Katsumata N, Uchitomi Y. Effect of communication skills training program for oncologists based on patient preferences for communication when receiving bad news: a randomized controlled trial. *J Clin Oncol*. 2014;32(20):2166-2172. doi:10.1200/JCO.2013.51.2756
37. Geoffroy PA, Delyon J, Strullu M, et al. Standardized Patients or Conventional Lecture for Teaching Communication Skills to Undergraduate Medical Students: A Randomized Controlled Study. *Psychiatry Investig*. 2020;17(4):299-305. doi:10.30773/pi.2019.0258
38. Giordani PJ. *Individual and organizational level moderators of physician communication skills training.* Order No. 3061572 ed. University of California, Riverside; 2002.<http://myaccess.library.utoronto.ca/login?qurl=https%3A%2F%2Fwww.proquest.com%2Fdissertations-theses%2Findividual-organizational-level-moderators%2Fdocview%2F304802857%2Fse-2%3Faccountid%3D14771>
39. Goelz T, Wuensch A, Stubenrauch S, et al. Specific training program improves oncologists' palliative care communication skills in a randomized controlled trial. *J Clin Oncol*. 2011;29(25):3402-3407. doi:10.1200/JCO.2010.31.6372
40. Green MJ, Levi BH. Teaching advance care planning to medical students with a computer-based decision aid. *J Cancer Educ*. 2011;26(1):82-91. doi:10.1007/s13187-010-0146-2
41. Grossman CE, Lemay M, Kang L, et al. Improv to improve medical student communication. *Clin Teach*. 2021; 18: 301–306. <https://doi-org.myaccess.library.utoronto.ca/10.1111/tct.13336>
42. *Haskard, K. B., Williams, S. L., DiMatteo, M. R., Rosenthal, R., White, M. K., & Goldstein, M. G. (2008). Physician and patient communication training in primary care: effects on participation and satisfaction. *Health psychology : official journal of the Division of Health Psychology, American Psychological Association*, *27*(5), 513–522. https://doi.org/10.1037/0278-6133.27.5.513
43. Helitzer DL, Lanoue M, Wilson B, de Hernandez BU, Warner T, Roter D. A randomized controlled trial of communication training with primary care providers to improve patient-centeredness and health risk communication. *Patient Educ Couns*. 2011;82(1):21-29. doi:10.1016/j.pec.2010.01.021
44. Henry S, Fenton J, Campbell C, et al.. Development and Testing of a Communication Intervention to Improve Chronic Pain Management in Primary Care. *The Clinical Journal of Pain.* 2022; 38 (10): 620-631. doi: 10.1097/AJP.0000000000001064.
45. Henselmans I, van Laarhoven HWM, de Haes HCJM, et al. Training for Medical Oncologists on Shared Decision-Making About Palliative Chemotherapy: A Randomized Controlled Trial. *Oncologist*. 2019;24(2):259-265. doi:10.1634/theoncologist.2018-0090
46. Henselmans I, van Laarhoven HWM, van Maarschalkerweerd P, et al. Effect of a Skills Training for Oncologists and a Patient Communication Aid on Shared Decision Making About Palliative Systemic Treatment: A Randomized Clinical Trial. *Oncologist*. 2020;25(3):e578-e588. doi:10.1634/theoncologist.2019-0453
47. Herrmann-Werner A, Weber H, Loda T, et al. "But Dr Google said…" - Training medical students how to communicate with E-patients. *Med Teach*. 2019;41(12):1434-1440. doi:10.1080/0142159X.2018.1555639
48. Higgins HM. *Empathy Training and Stress : Their Role in Medical Students’ Responses to Emotional Patients*. University of British Columbia; 1990. doi:[10.14288/1.0076869](https://doi.org/10.14288/1.0076869)
49. Hilgenberg SL, Bogetz AL, Leibold C, Gaba D, Blankenburg RL. De-escalating Angry Caregivers: A Randomized Controlled Trial of a Novel Communication Curriculum for Pediatric Residents. *Acad Pediatr*. 2019;19(3):283-290. doi:10.1016/j.acap.2018.10.005
50. Ho MJ, Yao G, Lee KL, Beach MC, Green AR. Cross-cultural medical education: can patient-centered cultural competency training be effective in non-Western countries?. *Med Teach*. 2008;30(7):719-721. doi:10.1080/01421590802232842
51. Hobgood CD, Tamayo-Sarver JH, Hollar DW Jr, Sawning S. Griev_Ing: death notification skills and applications for fourth-year medical students. *Teach Learn Med*. 2009;21(3):207-219. doi:10.1080/10401330903018450
52. Hobma S, Ram P, Muijtjens A, van der Vleuten C, Grol R. Effective improvement of doctor-patient communication: a randomised controlled trial. *Br J Gen Pract*. 2006;56(529):580-586.
53. Hojat M, Axelrod D, Spandorfer J, Mangione S. Enhancing and sustaining empathy in medical students. *Med Teach*. 2013;35(12):996-1001. doi:10.3109/0142159X.2013.802300
54. Intrieri RC, Kelly JA, Brown MM, Castilla C. Improving medical students' attitudes toward and skills with the elderly. *Gerontologist*. 1993;33(3):373-378. doi:10.1093/geront/33.3.373
55. Jenkins V, Fallowfield L. Can communication skills training alter physicians' beliefs and behavior in clinics?. *J Clin Oncol*. 2002;20(3):765-769. doi:10.1200/JCO.2002.20.3.765
56. Kaltman S, Talisman N, Pennestri S, Syverson E, Arthur P, Vovides Y. Using Technology to Enhance Teaching of Patient-Centered Interviewing for Early Medical Students. *Simul Healthc*. 2018;13(3):188-194. doi:10.1097/SIH.0000000000000304
57. Karkowsky CE, Landsberger EJ, Bernstein PS, et al. Breaking Bad News in obstetrics: a randomized trial of simulation followed by debriefing or lecture. *J Matern Fetal Neonatal Med*. 2016;29(22):3717-3723. doi:10.3109/14767058.2016.1141888
58. Kron FW, Fetters MD, Scerbo MW, et al. Using a computer simulation for teaching communication skills: A blinded multisite mixed methods randomized controlled trial. *Patient Educ Couns*. 2017;100(4):748-759. doi:10.1016/j.pec.2016.10.024
59. Lai MMY, Roberts N, Mohebbi M, Martin J. A randomised controlled trial of feedback to improve patient satisfaction and consultation skills in medical students. *BMC Med Educ*. 2020;20(1):277. Published 2020 Aug 20. doi:10.1186/s12909-020-02171-9
60. Langewitz W, Nübling M, Weber H. A theory-based approach to analysing conversation sequences. *Epidemiol Psichiatr Soc*. 2003;12(2):103-108. doi:10.1017/s1121189x00006163
61. Langewitz WA, Eich P, Kiss A, Wossmer B. Improving Communication Skills-A Randomized Controlled Behaviorally Oriented Intervention Study for Residents in Internal Medicine. *Psychosomatic Medicine*. 1998;60(3):268.
62. Legg C, Young L, Bryer A. Training sixth-year medical students in obtaining case-history information from adults with aphasia. *Aphasiology*. 2005;19(6):559-575. doi:[10.1080/02687030544000029](https://doi.org/10.1080/02687030544000029)
63. Libert Y, Peternelj L, Bragard I, et al. A randomized controlled trial assessing behavioral, cognitive, emotional and physiological changes resulting from a communication skills training in physicians caring for cancer patients. *Patient Educ Couns*. 2022;105(9):2888-2898. doi:10.1016/j.pec.2022.04.012
64. Liénard A, Merckaert I, Libert Y, et al. Is it possible to improve residents breaking bad news skills? A randomised study assessing the efficacy of a communication skills training program. *Br J Cancer*. 2010;103(2):171-177. doi:10.1038/sj.bjc.6605749
65. Liu C, Lim RL, McCabe KL, Taylor S, Calvo RA. A Web-Based Telehealth Training Platform Incorporating Automated Nonverbal Behavior Feedback for Teaching Communication Skills to Medical Students: A Randomized Crossover Study. *J Med Internet Res*. 2016;18(9):e246. Published 2016 Sep 12. doi:10.2196/jmir.6299
66. LoSasso AA, Lamberton CE, Sammon M, et al. Enhancing Student Empathetic Engagement, History-Taking, and Communication Skills During Electronic Medical Record Use in Patient Care. *Acad Med*. 2017;92(7):1022-1027. doi:10.1097/ACM.0000000000001476
67. Lozano P, McPhillips HA, Hartzler B, et al. Randomized trial of teaching brief motivational interviewing to pediatric trainees to promote healthy behaviors in families. *Arch Pediatr Adolesc Med*. 2010;164(6):561-566. doi:10.1001/archpediatrics.2010.86\
68. Maatouk-Bürmann B, Ringel N, Spang J, et al. Improving patient-centered communication: Results of a randomized controlled trial. *Patient Education and Counseling*. 2016;99(1):117-124. doi:[10.1016/j.pec.2015.08.012](https://doi.org/10.1016/j.pec.2015.08.012)
69. Maguire P, Fairbairn S, Fletcher C. Consultation Skills Of Young Doctors: I: Benefits Of Feedback Training In Interviewing As Students Persist. *British Medical Journal (Clinical Research Edition)*. 1986;292(6535):1573-1576.
70. Malhotra C, Rajasekaran T, Kanesvaran R, et al. Pilot Trial of a Combined Oncologist-Patient-Caregiver Communication Intervention in Singapore. *JCO Oncol Pract*. 2020;16(2):e190-e200. doi:10.1200/JOP.19.00412
71. Marsh M, Lauden SM, Mahan JD, et al. Family-centered communication: A pilot educational intervention using deliberate practice and patient feedback. *Patient Education and Counseling*. 2021;104(5):1200-1205. doi:[10.1016/j.pec.2020.09.033](https://doi.org/10.1016/j.pec.2020.09.033)
72. Marteau TM, Humphrey C, Matoon G, Kidd J, Lloyd M, Horder J. Factors influencing the communication skills of first-year clinical medical students. *Med Educ*. 1991;25(2):127-134. doi:10.1111/j.1365-2923.1991.tb00038.x
73. Matharu K, Shapiro JF, Hammer RR, Kravitz RL, Wilson MD, Fitzgerald FT. Reducing obesity prejudice in medical education. *Educ Health (Abingdon)*. 2014;27(3):231-237. doi:10.4103/1357-6283.152176
74. *Merckaert I, Libert Y, Delvaux N, et al. Factors influencing physicians' detection of cancer patients' and relatives' distress: can a communication skills training program improve physicians' detection?. *Psychooncology*. 2008;17(3):260-269. doi:10.1002/pon.1233
75. Moral RR, Alamo MM, Jurado MA, de Torres LP. Effectiveness of a learner-centred training programme for primary care physicians in using a patient-centred consultation style. *Fam Pract*. 2001;18(1):60-63. doi:10.1093/fampra/18.1.60
76. Moreland JR. *Video Programmed Instruction in Elementary Psychotherapeutic and Related Clinical Skills.* University of Massachusetts Amherst; 1971. doi:[10.7275/1QE0-TH33](https://doi.org/10.7275/1QE0-TH33)
77. Moulton CA, Tabak D, Kneebone R, Nestel D, MacRae H, LeBlanc VR. Teaching communication skills using the integrated procedural performance instrument (IPPI): a randomized controlled trial. *Am J Surg*. 2009;197(1):113-118. doi:10.1016/j.amjsurg.2008.09.006
78. Nayiga S, DiLiberto D, Taaka L, et al. Strengthening patient-centred communication in rural Ugandan health centres: A theory-driven evaluation within a cluster randomized trial. *Evaluation (Lond)*. 2014;20(4):471-491. doi:10.1177/1356389014551484
79. Nikendei C, Bosse HM, Hoffmann K, et al. Outcome of parent-physician communication skills training for pediatric residents. *Patient Educ Couns*. 2011;82(1):94-99. doi:10.1016/j.pec.2009.12.013
80. Patel MR, Song PXK, Bruzzese JM, et al. Does cross-cultural communication training for physicians improve pediatric asthma outcomes? A randomized trial. *Journal of Asthma*. 2019;56(3):273-284. doi:[10.1080/02770903.2018.1455856](https://doi.org/10.1080/02770903.2018.1455856)
81. Perera J, Mohamadou G, Kaur S. The use of objective structured self-assessment and peer-feedback (OSSP) for learning communication skills: evaluation using a controlled trial. *Adv Health Sci Educ Theory Pract*. 2010;15(2):185-193. doi:10.1007/s10459-009-9191-1
82. Pirdehghan A, Vakili M, Mirzaei M, Karimi M. The effect of training in communication skills on medical students: A randomized controlled trial. *International Journal of Pharmaceutical Research*. 2018;10:339-343.
83. Pollak KI, Coffman CJ, Tulsky JA, et al. Teaching Physicians Motivational Interviewing for Discussing Weight With Overweight Adolescents. *J Adolesc Health*. 2016;59(1):96-103. doi:10.1016/j.jadohealth.2016.03.026
84. Potash JS, Chen JY, Lam CL, Chau VT. Art-making in a family medicine clerkship: how does it affect medical student empathy? *BMC Medical Education*. 2014;14(1):247. doi:[10.1186/s12909-014-0247-4](https://doi.org/10.1186/s12909-014-0247-4)
85. Price EG, Windish DM, Magaziner J, Cooper LA. Assessing validity of standardized patient ratings of medical students' communication behavior using the Roter interaction analysis system. *Patient Educ Couns*. 2008;70(1):3-9. doi:10.1016/j.pec.2007.10.002
86. Qureshi AA, Zehra T. Simulated patient’s feedback to improve communication skills of clerkship students. *BMC Medical Education*. 2020;20(1):15. doi:[10.1186/s12909-019-1914-2](https://doi.org/10.1186/s12909-019-1914-2)
87. Rassbach CE, Bogetz AL, Orlov N, et al. The Effect of Faculty Coaching on Resident Attitudes, Confidence, and Patient-Rated Communication: A Multi-Institutional Randomized Controlled Trial. *Acad Pediatr*. 2019;19(2):186-194. doi:10.1016/j.acap.2018.10.004
88. *Razavi D, Merckaert I, Marchal S, et al. How to optimize physicians' communication skills in cancer care: results of a randomized study assessing the usefulness of posttraining consolidation workshops. *J Clin Oncol*. 2003;21(16):3141-3149. doi:10.1200/JCO.2003.08.031
89. Reed DL. Effects of a Combined Didactic and Experiential Death Education/Empathy Training Program on Death Anxiety and Empathic Ability of Medical Students. *Drake University;* 1996. Accessed January 31, 2024.<http://hdl.handle.net/2092/425>
90. **Robins LS, Wolf FM. The effect of training on medical students' responses to geriatric patient concerns: results of a linguistic analysis. *Gerontologist*. 1989;29(3):341-344. doi:10.1093/geront/29.3.341
91. Roche AM, Stubbs JM, Sanson-Fisher RW, Saunders JB. A controlled trial of educational strategies to teach medical students brief intervention skills for alcohol problems. *Prev Med*. 1997;26(1):78-85. doi:10.1006/pmed.1996.9990
92. Roter DL, Cole KA, Kern DE, Barker LR, Grayson M. An evaluation of residency training in interviewing skills and the psychosocial domain of medical practice. *J Gen Intern Med*. 1990;5(4):347-354. doi:10.1007/BF02600404
93. Sanson-Fisher RW, Poole AD. Training medical students to empathize: an experimental study. *Med J Aust*. 1978;1(9):473-476.
94. Schouten BC, Meeuwesen L, Harmsen HA. The impact of an intervention in intercultural communication on doctor-patient interaction in The Netherlands. *Patient Educ Couns*. 2005;58(3):288-295. doi:10.1016/j.pec.2005.06.005
95. Sepucha K, Han PKJ, Chang Y, et al. Promoting Informed Decisions About Colorectal Cancer Screening in Older Adults (PRIMED Study): a Physician Cluster Randomized Trial. *J Gen Intern Med*. 2023;38(2):406-413. doi:10.1007/s11606-022-07738-4
96. Servotte JC, Bragard I, Szyld D, et al. Efficacy of a Short Role-Play Training on Breaking Bad News in the Emergency Department. *West J Emerg Med*. 2019;20(6):893-902. Published 2019 Oct 14. doi:10.5811/westjem.2019.8.43441
97. Shapiro SM, Lancee WJ, Richards-Bentley CM. Evaluation of a communication skills program for first-year medical students at the University of Toronto. *BMC Medical Education*. 2009;9(1):11. doi:[10.1186/1472-6920-9-11](https://doi.org/10.1186/1472-6920-9-11)
98. Smith RC, Lyles JS, Mettler JA, et al. A strategy for improving patient satisfaction by the intensive training of residents in psychosocial medicine: a controlled, randomized study. *Acad Med*. 1995;70(8):729-732. doi:10.1097/00001888-199508000-00019
99. Stewart M, Brown JB, Hammerton J, et al. Improving communication between doctors and breast cancer patients. *Ann Fam Med*. 2007;5(5):387-394. doi:10.1370/afm.721
100. Strohbehn GW, Hoffman SJK, Tokaz M, et al. Visual arts in the clinical clerkship: a pilot cluster-randomized, controlled trial. *BMC Medical Education*. 2020;20(1):481. doi:[10.1186/s12909-020-02386-w](https://doi.org/10.1186/s12909-020-02386-w)
101. Sullivan MD, Gaster B, Russo J, et al. Randomized trial of web-based training about opioid therapy for chronic pain. *Clin J Pain*. 2010;26(6):512-517. doi:10.1097/AJP.0b013e3181dc7adc
102. Szmuilowicz E, el-Jawahri A, Chiappetta L, Kamdar M, Block S. Improving residents' end-of-life communication skills with a short retreat: a randomized controlled trial. *J Palliat Med*. 2010;13(4):439-452. doi:10.1089/jpm.2009.0262
103. Szmuilowicz E, Neely KJ, Sharma RK, Cohen ER, McGaghie WC, Wayne DB. Improving residents' code status discussion skills: a randomized trial. *J Palliat Med*. 2012;15(7):768-774. doi:10.1089/jpm.2011.0446
104. Tavakoly Sany SB, Behzhad F, Ferns G, Peyman N. Communication skills training for physicians improves health literacy and medical outcomes among patients with hypertension: a randomized controlled trial. *BMC Health Serv Res*. 2020;20(1):60. Published 2020 Jan 23. doi:10.1186/s12913-020-4901-8
105. Tulsky JA, Arnold RM, Alexander SC, et al. Enhancing communication between oncologists and patients with a computer-based training program: a randomized trial. *Ann Intern Med*. 2011;155(9):593-601. doi:10.7326/0003-4819-155-9-201111010-00007
106. Vincent A, Urben T, Becker C, et al. Breaking bad news: A randomized controlled trial to test a novel interactive course for medical students using blended learning. *Patient Educ Couns*. 2022;105(1):105-113. doi:10.1016/j.pec.2021.05.002
107. Wolf FM, Woolliscroft JO, Calhoun JG, Boxer GJ. A controlled experiment in teaching students to respond to patients' emotional concerns. *J Med Educ*. 1987;62(1):25-34. doi:10.1097/00001888-198701000-00004
108. Wong SY, Cheung AK, Lee A, et al. Improving general practitioners' interviewing skills in managing patients with depression and anxiety: a randomized controlled clinical trial. *Med Teach*. 2007;29(6):e175-e183. doi:10.1080/01421590601050585
109. Wuensch A, Goelz T, Ihorst G, et al. Effect of individualized communication skills training on physicians' discussion of clinical trials in oncology: results from a randomized controlled trial. *BMC Cancer*. 2017;17(1):264. Published 2017 Apr 13. doi:10.1186/s12885-017-3238-0
110. Wündrich M, Schwartz C, Feige B, Lemper D, Nissen C, Voderholzer U. Empathy training in medical students - a randomized controlled trial. *Med Teach*. 2017;39(10):1096-1098. doi:10.1080/0142159X.2017.1355451
111. Yu MX, Jiang XY, Li YJ, Shen ZY, Zhuang SQ, Gu YF. Evaluation of medical record quality and communication skills among pediatric interns after standardized parent training history-taking in China. *Med Teach*. 2018;40(2):188-192. doi:10.1080/0142159X.2017.1395833

Note: All these studies were included in the Network-Meta Analysis. Three studies were not included in the Pairwise Meta-Analysis because they did not have a control comparator: Hilgenberg et al., 2018; Hobgood et al., 2009 and Lai et al., 2020.

*These studies shared the same sample and therefore treated as one study: Merckaert et al., 2008 and Razavi et al., 2003; and Bonvinci, 2007 and Haskard et al., 2008

**There studies reported mulitple independent samples of data: Blatt et al., 2010 reported three independent samples. Robins & Wolf, 1989 reported 2 independent samples.

**Supplemental Appendix 4**

# Definitions of Study Variables

| Variable | Categories | Definition/Coding Criteria |
| --- | --- | --- |
| Didactic | Yes/No | Participants were given oral or written information about empathetic interactions (e.g.,  presentation, pamphlet or workbook with relevant information). |
| Rehearsal | Yes/No | Participants had the opportunity to practice  empathy-skills (e.g., roleplay  exercises, drama, directly with clients) |
| Reflection | Yes/No | Participants had the opportunity to reflect on and discuss ideas of empathy and their own  experiences interacting with clients, either with other participants or facilitators (e.g., brainstorming, problem-solving, talking about case studies). |
| Observation | Yes/No | Participants watch or listen to others interacting and/or performing the skill they are trying to learn (e.g., video vignettes, live demonstration) |
| Feedback | Yes/No | Participants received feedback (from other  participants and/or facilitators) about their  performance exhibiting responsivity in interactions |
| Format | Group | Program was delivered by one or a few facilitators in person with trainees at a time |
|  | Individual | Program was delivered by one facilitator in person to one trainee in one-to-one setting |
|  | Group + Individual | Program was delivered in person using some group sessions and some individual sessions. |
|  | Online/Independent | Program was delivered primarily online format and/or involved independent study (e.g., self-learning, no facilitator) |
| Facilitator Type | Professional | Program was delivered by a trained professional (e.g., psychologist, social worker). |
|  | Para-professional | Program was delivered by trained community  members, peer, or student with training |
|  | Researcher | Program was delivered by one of the study authors |
|  | Other | Program was not led by a facilitator (i.e., self-lead, independent, and/or online) |
| Number of Sessions | Categorical | The number of separate “training sessions” participants were supposed to attend, i.e., the number of different times participants met/engaged with the content. A session was counted as a distinct occasion on which  the participant engaged with the facilitator, or program content E.g., a session of 30 minutes and a session of 1 hour both equal 1 session. |
| Measurement Type | Objective | Participant completes a test of their ability (e.g., answering how they would respond to a case study) or an observation measure that where participant interacts with someone (e.g., real client, peer, actor). An independent researcher rates their performance. |
|  |  |  |
|  | Other-report | The person interacting with the participant (e.g., real patient, peer, actor) answers questions about the participant’s ability level. |
|  | Self-Report | Participant answers questions about their own ability level. |
|  | Mixed | The study utilizes a more than one of the above measurement types |
| Number of teaching methods | Categorical | Number of teaching methods used in an intervention (up to five). |
|  |  |  |
|  |  |  |
| Risk of Bias | High Risk | Four or more of the seven Cochrane categories for risk of bias assessment were determined to have a high or unclear level of risk. |
|  | Low Risk | Four or more of the seven Cochrane categories for risk of bias assessment were determined to be low risk. |
| Education Level | Professional | Participants are in professional practice |
|  | Resident | Participants are training at the residency level |
|  | Postgraduate | Participants are in medical school at the postgraduate level |
|  | Undergraduate | Participants are in medical school at the undergraduate level |
|  | Mixed | Participants are at various levels of education |
| Type of  Control Group | Control | Control group received absolutely no treatment at all (e.g., waitlist). |
|  | Active Control | Control group received similar a format of  programming as the intervention group, but topic is unrelated to empathy skills (e.g., stress management). |
|  | Education As Usual | Control group received the usual curriculum in medical communication skills (e.g., lecture-based training). |
|  |  |  |

#

# Supplemental Appendix 5

**Table 5.1.** Counts and Percentages of Study and Participant Characteristics, *k* = 109

|  | Count | Percentage |
| --- | --- | --- |
| **Study Characteristics** |  |  |
| Year of Publication |  |  |
| Before 1980 | 2 | 1.8% |
| 1980-1989 | 3 | 2.8% |
| 1990-1999 | 9 | 8.3% |
| 2000-2009 | 21 | 19.3% |
| 2010-2019 | 57 | 52.3% |
| After 2020 | 17 | 15.6% |
| Country Region |  |  |
| North America | 56 | 51.4% |
| Europe & Central Asia | 31 | 28.4% |
| East Asia and Pacific | 13 | 11.9% |
| Middle East & North Africa | 4 | 3.7% |
| South Asia | 2 | 1.8% |
| Sub-Saharan Africa | 2 | 1.8% |
| Mixed | 1 | 0.9% |
| Source |  |  |
| Dissertation | 3 | 0.3% |
| Journal | 106 | 97.2% |
| Sample Size |  |  |
| <25 | 10 | 9.2% |
| 25-49 | 34 | 31.2% |
| 50-99 | 32 | 29.4% |
| 100-149 | 13 | 11.9% |
| 150-199 | 8 | 7.3% |
| 200-249 | 3 | 2.8% |
| 250-299 | 2 | 1.8% |
| 300+ | 7 | 6.4% |
| **Participant Characteristics** |  |  |
| Education Level |  |  |
| Undergraduate | 14 | 12.8% |
| Postgraduate | 31 | 28.4% |
| Resident | 24 | 22% |
| Professional | 30 | 27.5% |
| Mixed | 10 | 9.2% |
| Sex (% Female) |  |  |
| <50% | 34 | 31.2% |
| 50-74% | 45 | 41.3% |
| 75-100% | 5 | 4.6% |
| Not Reported | 25 | 22.9% |
| Ethnicity |  |  |
| European American > 50% | 16 | 14.7% |
| Other ethnicity > 50% | 9 | 8.3% |
| Not Reported | 84 | 77.1% |

# Supplemental Appendix 6

# Individual Study Information

# Table 6.1*.* Study and Sample Characteristics by Study

| Study | N (Randomized) | Sex  (% Female) | Mean Age | Ethnicity Percentages | Education Level |
| --- | --- | --- | --- | --- | --- |
| Abbasi et al., 2018 | 60 | 40.2 | 34.07 | NR | Resident |
| Alder et al., 2007 | 32 | NR | NR | NR | Mixed |
| Annadurai et al., 2021 | 22 | 32 | 43.95 | 73% White, 36% Asian, 4.5% Black, 9% Hispanic | Professional |
| Bashour et al., 2013 | 137 | NR | NR | NR | Resident |
| Beach et al., 2015 | 26 | 48 | 46 | 71% White | Professional |
| Bearman et al., 2001 | 284 | NR | NR | NR | Post-grad |
| Bibl et al., 2021 | 17 | 65 | NR | NR | Mixed |
| Bickell et al., 2020 | 4 | 31.5 | 43.9 | White 73% | Professional |
| Blatt et al., 2010 ^a^ | 608 | 60.69 | NR | 31.91% African American, 46.22% White, 21.88% other race | Post-grad |
| Blödt et al., 2016 | 42 | 80 | NR | NR | Professional |
| Bonvicini, 2007 | 156 | 37 | 37.3 | NR | Professional |
| Bosse et al., 2012 | 103 | 38.78 | 24.55 | NR | Undergrad |
| Bowyer et al., 2010 | 553 | NR | NR | NR | Post-grad |
| Brinkman et al., 2007 | 36 | 66.67 | 28.3 | White non-Hispanic: 52.78%, Black non-Hispanic: 5.56%, Hispanic: 11.11%, Asian: 19.44%, Other: 8.33% | Resident |
| Brown et al., 1999 | 69 | 36.07 | NR | NR | Professional |
| Brown et al., 2018 | 472 | 59.27 | 33.83 | 21% of the participants from the intervention group and 24% of the participants from the control group belonged to an ethnic minority. | Mixed |
| Butow et al., 2008 | 30 | 50 | NR | NR | Professional |
| Butow et al., 2015 | 62 | 58 | NR | NR | Professional |
| Cave et al., 2007 | 359 | NR | NR | NR | Post-grad |
| Cooper et al., 2011 | 41 | 53.66 | 42.96 | 29.27% African American, 24.39% Asian, 43.9% White, 2.44% Hispanic/Latino | Professional |
| Coret et al., 2018 | 85.19 | 23.1 | 3 | NR | Post-grad |
| D’souza et al,. 2019 | 71 | 49.3 | 19.48 | NR | Undergrad |
| Daeppen et al., 2012 | 131 | 59.3 | 24.7 | NR | Post-grad |
| Daetwyler et al., 2010 | 54 | 27.27 | 29 | NR | Resident |
| Dominick et al., 2018 | 68 | 69.12 | NR | Hispanic = 1.47%, Asian = 13.2%, African American = 2.94%, Caucasian = 70.59%, Other = 11.76 | Undergrad |
| Downar et al., 2017 | 94 | NR | NR | NR | Resident |
| du Vaure et al., 2017 | 352 | 59.53 | NR | NR | Undergrad |
| Dubosh et al., 2019 | 64 | 41 | NR | NR | Undergrad |
| Duran et al., 2020 | 30 | NR | NR | NR | Resident |
| Dwyer et al., 2019 | 71 | 63.38 | NR | NR | Mixed |
| Epinat-Duclos et al., 2021 | 158 | 76 | NR | NR | Undergrad |
| Farrell et al., 2014 | 49 | 36.74 | 29.49 | NR | Professional |
| Flocke et al., 2014 | 31 | 48 | 47 | 87% white | Professional |
| Foster et al., 2015 | 67 | 63.5 | 24.1 | 36.5% Asian, 9.5% Black, 54% White | Post-grad |
| Foster et al., 2016 | 70 | 47.14 | 23.43 | 1.43% Indian, 34.29% Asian, 2.86% Black, 61.43% White | Post-grad |
| Fujimori et al., 2014 | 30 | 13.3 | NR | NR | Professional |
| Geoffroy et al., 2020 | 388 | NR | NR | NR | Undergrad |
| Giordani, 2002 | 150 | 38.05 | 36.7 | Hispanic/Latino:4.02 %, African American: 0.66%, Caucasian: 43.01%, Asian: 6.68%, Indian Subcontinent: 38.96%, Other: 8.08% | Mixed |
| Goelz et al., 2011 | 41 | 56.1 | 30.95 | NR | Professional |
| Green & Levi, 2011 | 121 | 50 | 25 | NR | Post-grad |
| Grossman et al., 2021 | 188 | NR | NR | NR | Post-grad |
| Helitzer et al., 2011 | 27 | 50 | NR | NR | Professional |
| Henry et al., 2022 | 47 | 64 | 29.7 | 47% Asian/Pacific Islander, and 27% non-Hispanic White | Resident |
| Henselmans et al., 2019 | 31 | 74.2 | 40.6 | NR | Post-grad |
| Henselmans et al., 2020 | 31 | 73.1 | 41.5 | NR | Mixed |
| Herrmann-Werner et al., 2019 | 46 | 74 | 25.4 | NR | Post-grad |
| Higgins et al., 1990 | 18 | 53.85 | 25.5 | NR | Post-grad |
| Hilgenberg et al., 2018 | 84 | 77.38 | NR | (Non-Hispanic) white = 60.71%, Hispanic or Latino = 7.14, Asian = 22.62, Black or African American = 4.76, Other = 2.38, Prefer not to answer = 7.14 | Resident |
| Ho et al., 2008 | 57 | 20 | 25 | 87% Taiwanese | Undergrad |
| Hobgood et al., 2009 | 138 | 53.62 | 28.2 | 12.31% African American, 76.81% White, 10.88% Other | Post-grad |
| Hobma et al., 2006 | 100 | NR | NR | NR | Professional |
| Hojat et al., 2013 | 248 | 51 | NR | NR | Post-grad |
| Intrieri et al., 1993 | 96 | 25 | 26.12 | NR | Post-grad |
| Jenkins & Fallowfield, 2002 | 93 | 30.1 | NR | NR | Professional |
| Kaltman et al., 2018 | 99 | 54.5 | 24.46 | White = 78%, Asian = 17% | Post-grad |
| Karkowsky et al., 2016 | 16 | 94.12 | 36.89 | NR | Resident |
| Kron et al., 2017 | 435 | 45.52 | 25.45 | 52.64% Caucasian-American, 19.54% Asian-American, 8.97% African-American, 0.69% Native-American/Indigenous people, and 14.94% other/no response | Post-grad |
| Lai et al., 2020 | 71 | 52 | 23.1 | NR | Resident |
| Langewitz et al., 1998 | 43 | 19.05 | 32.87 | NR | Resident |
| Langewitz et al., 2003 | 43 | NR | NR | NR | Resident |
| Legg et al., 2005 | 24 | 63.5 | 25.25 | 36% white, 41% black, 13% Indian, 10% other | Post-grad |
| Libert et al., 2022 | 90 | 68 | 45 | NR | Professional |
| Liénard et al., 2010 | 98 | 72.68‬ | 28 | NR | Resident |
| Liu et al., 2016 | 268 | 57.46 | NR | NR | Undergrad |
| LoSasso et al., 2017 | 70 | NR | NR | NR | Post-grad |
| Lozano, et al., 2010 | 18 | 75 | NR | NR | Resident |
| Maatouk-Bürmann et al., 2016 | 42 | 50 | 33.67 | NR | Mixed |
| Maguire et al., 1986 | 36 | NR | NR | NR | Professional |
| Malhotra et al., 2019 | 10 | 50 | 32.9 | Chinese = 80%, Malay/Indian/other = 20% | Professional |
| Marsh et al., 2021 | 38 | 72 | NR | NR | Resident |
| Marteau et al., 1991 | 88 | 43.84 | NR | NR | Post-grad |
| Matharu et al., 2014 | 136 | 70 | 25.15 | NR | Post-grad |
| Merckaert et al., 2008; Razavi et al., 2003 ^b^ | 72 | 43 | 43 | NR | Professional |
| Moral et al., 2001 | 20 | 60 | 39.85 | NR | Professional |
| Moreland, 1971 | 24 | 0 | NR | NR | Post-grad |
| Moulton et al., 2009 | 32 | NR | NR | NR | Mixed |
| Nayiga et al., 2014 | 57 | NR | NR | NR | Professional |
| Nikendei et al., 2011 | 28 | 64.29 | 31.6 | NR | Resident |
| Patel et al., 2019 | 112 | 57 | NR | White = 79.46, Other = 18.75% | Professional |
| Perera et al., 2010 | 202 | NR | NR | NR | Undergrad |
| Pirdehghan et al., 2018 | 60 | 58.3 | 25 | NR | Post-grad |
| Pollak et al., 2016 | 46 | 65 | 40.8 | 91% White | Professional |
| Potash et al., 2014 | 152 | 40.56 | NR | 99% Chinese; 1% South East Asian | Undergrad |
| Price et al., 2008 | 120 | 52.5 | 24.25 | 56% Caucasian | Post-grad |
| Qureshi & Zehra, 2020 | 80 | 51.25 | NR | NR | Post-grad |
| Rassbach et al., 2018 | 114 | 70.18 | NR | American Indian/Alaska Native = 0.88%, Asian = 20.78%, Black or African American = 7.02%, Hispanic or Latino = 6.14%, Native Hawaiian/Pacific Islander = 0.88%, White = 57.89, Other = 4.39, Data missing = 4.39 | Resident |
| Reed, 1996 | 27 | 48.15 | 28 | NR | Post-grad |
| Robins & Wolf, 1989 ^a^ | 193 | NR | NR | NR | Post-grad |
| Roche et al., 1997 | 123 | NR | NR | NR | Undergrad |
| Roter et al., 1990 | 48 | NR | NR | NR | Resident |
| Sanson‐Fisher & Poole, 1978 | 135 | 33.03% | NR | NR | Post-grad |
| Schouten et al., 2005 | 38 | 21.1 | NR | NR | Professional |
| Sepucha et al., 2022 | 67 | 51 | 52.75 | NR | Professional |
| Servotte et al., 2019 | 73 | NR | NR | NR | Mixed |
| Shapiro et al., 2009 | 95 | 61 | 23.1 | NR | Post-grad |
| Smith et al., 1005 | 26 | 42.31 | NR | NR | Resident |
| Stewart et al., 2007 | 51 | 33.33 | NR | NR | Professional |
| Strohbehn et al., 2020 | 35 | 53 | NR | 68% White, 21% Asian, 12% Other | Resident |
| Sullivan et al., 2010 | 213 | 45.1 | NR | NR | Resident |
| Szmuilowicz et al., 2010 | 38 | 39.47 | 26.75 | NR | Resident |
| Szmuilowicz et al., 2012 | 88 | 8.7 | 40.3 | 82.61% White, 17.39% Minority | Resident |
| Tavakoly Sany et al., 2020 | 35 | 64 | 37.08 | NR | Resident |
| Tulsky et al., 2011 | 48 | 19 | 49.25 | 79.5% White, 14.5% Asian or Pacific Islander, 8.5% Hispanic, 8.5% other | Professional |
| Vincent et al., 2022 | 160 | 55 | 22.8 | NR | Undergrad |
| Wolf et al., 1987 | 134 | NR | NR | NR | Post-grad |
| Wong et al., 2007 | 40 | 40.625 | NR | NR | Professional |
| Wuensch et al., 2017 | 40 | 55 | 32.5 | NR | Professional |
| Wündrich et al., 2017 | 158 | NR | NR | NR | Mixed |
| Yu et al., 2018 | 60 | NR | NR | NR | Undergrad |

Abbreviation*:*  NR = not reported, Post-grad = postgraduate studies.

^a^This article reported on independent samples within, so were treated as separate studies in the analysis.

^b^These two articles shared the same sample.

**Table 6.2**. Intervention and Outcome Characteristics by Study

| Study | Arm 1 | Arm 2 | Arm 3 | Format | Facilitator Type | Number of Sessions | Assessment Type |
| --- | --- | --- | --- | --- | --- | --- | --- |
| Abbasi et al., 2018 | D | C |  | in-person group | Professional | <5 | self-report |
| Alder et al., 2007 | DHROF | C |  | in-person group + indiv. | Researcher | <5 | objective |
| Annadurai et al., 2021 | DHOF | EAU |  | in-person group + indiv. | Para-professional | 5 to 10 | objective |
| Bashour et al., 2013 | DHR | C |  | in-person group | Professional | <5 | other-report |
| Beach et al., 2015 | DO | C |  | in-person group | Researcher | <5 | objective |
| Bearman et al., 2001 | H^c^ | C |  | in-person group | Professional | 5 to 10 | objective |
| Bibl et al., 2021 | DHOF | C |  | in-person group + indiv. | Para-professional | <5 | objective |
| Bickell et al., 2020 | DHROF | EAU |  | in-person group + indiv. | Researcher | 5 to 10 | other-report |
| Blatt et al., 2010 ^a^ | DHR | AC |  | in-person group | Professional | <5 | other-report |
| Blödt et al., 2016 | DHROF | C |  | in-person group | Professional | 10+ | objective |
| Bonvicini, 2007 | DHF | C |  | in-person group | Professional | <5 | objective |
| Bosse et al., 2012 | DHRF^c^ | AC |  | in-person group | Professional | 5 to 10 | objective |
| Bowyer et al., 2010 | DRO^c^ | O | C | in-person group + indiv. | Professional | <5 | other-report |
| Brinkman et al., 2007 | DHRF | AC |  | in-person ind. | Professional | <5 | other-report |
| Brown et al., 1999 | DH | C |  | in-person group + indiv. | Professional | <5 | other-report |
| Brown et al., 2018 | DHRO | EAU |  | in-person group | Professional | 5 to 10 | self-report |
| Butow et al., 2008 | DHROF | C |  | in-person group | Professional | 5 to 10 | objective |
| Butow et al., 2015 | DHROF | C |  | in-person group + indiv. | Researcher | <5 | objective |
| Cave et al., 2007 | DRF | HF | EAU | in-person group | Professional | <5 | objective |
| Cooper et al., 2011 | DHF | EAU |  | in-person ind. | Para-Professional | NR | objective |
| Coret et al., 2018 | DHRO | EAU |  | in-person group + indiv. | Researcher | 5 to 10 | objective |
| D’souza et al,. 2019 | DHRO | C |  | in-person group | Researcher | <5 | self-report |
| Daeppen et al., 2012 | DHRO | EAU |  | in-person group | Professional | <5 | objective |
| Daetwyler et al., 2010 | DHOF | DO | C | online/ind. | Other | <5 | other-report |
| Dominick et al., 2018 | HO | EAU |  | in-person group | Professional | <5 | self-report |
| Downar et al., 2017 | DHROF | AC |  | in-person group + indiv. | Professional | <5 | objective |
| du Vaure et al., 2017 | H | EAU |  | in-person group | Para-Professional | 5 to 10 | other-report |
| Dubosh et al., 2019 | DF | EAU |  | online/ind. | Professional | <5 | other-report |
| Duran et al., 2020 | DHROF | EAU |  | in-person group + indiv. | Researcher | <5 | other-report |
| Dwyer et al., 2019 | DRO | C |  | online/ind. | Other | <5 | self-report |
| Epinat-Duclos et al., 2021 | DHR | AC |  | in-person group + indiv. | Para-professional | 5 to 10 | mixed |
| Farrell et al., 2014 | DHF | EAU |  | online/ind. | Other | <5 | objective |
| Flocke et al., 2014 | DHOF | AC |  | in-person group | Researcher | <5 | objective |
| Foster et al., 2015 | H | AC |  | online/ind. | Other | <5 | other-report |
| Foster et al., 2016 | DHF | DO | AC | online/ind. | Researcher | <5 | objective |
| Fujimori et al., 2014 | DHROF | C |  | in-person group | Professional | <5 | objective |
| Geoffroy et al., 2020 | HOF | EAU |  | in-person group | Professional | <5 | objective |
| Giordani, 2002 | DOF | C |  | in-person group + indiv. | Professional | <5 | objective |
| Goelz et al., 2011 | DHF | C |  | in-person group + indiv. | Professional | <5 | objective |
| Green & Levi, 2011 | H | EAU |  | online/ind. | Other | NR | other-report |
| Grossman et al., 2021 | HROF | EAU |  | in-person group | Professional | <5 | mixed |
| Helitzer et al., 2011 | DHROF | C |  | in-person group + indiv. | Researcher | <5 | objective |
| Henry et al., 2022 | DHF | EAU |  | in-person ind. | Para-professional | <5 | objective |
| Henselmans et al., 2019 | DHOF | EAU |  | in-person group | Professional | <5 | objective |
| Henselmans et al., 2020 | DHOF | EAU |  | in-person group + indiv. | Professional | <5 | objective |
| Herrmann-Werner et al., 2019 | DHROF | EAU |  | in-person group + indiv. | Professional | <5 | other-report |
| Higgins et al., 1990 | DHROF | C |  | in-person group | Professional | <5 | objective |
| Hilgenberg et al., 2018 | DHRO | DR |  | in-person group | Researcher | 5 to 10 | other-report |
| Ho et al., 2008 | DH | D | C | in-person group | Professional | <5 | objective |
| Hobgood et al., 2009 | DHRF^c^ | DHR |  | in-person group | Professional | <5 | other-report |
| Hobma et al., 2006 | DF | AC |  | in-person group + indiv. | Professional | <5 | objective |
| Hojat et al., 2013 | DR | R | AC | in-person group | Researcher | <5 | self-report |
| Intrieri et al., 1993 | DHR | C |  | in-person group | Professional | <5 | objective |
| Jenkins & Fallowfield, 2002 | DHROF | C |  | in-person group | Para-Professional | <5 | objective |
| Kaltman et al., 2018 | H | EAU |  | online/ind. | Other | <5 | objective |
| Karkowsky et al., 2016 | DRF | EAU |  | in-person ind. | Professional | <5 | other-report |
| Kron et al., 2017 | HR | AC |  | online/ind. | Other | <5 | objective |
| Lai et al., 2020 | DRF | F |  | online/ind. | Para-professional | 5 to 10 | objective |
| Langewitz et al., 1998 | DHOF | EAU |  | in-person group | Professional | 10+ | objective |
| Langewitz et al., 2003 | DHRF | EAU |  | in-person group + indiv. | Professional | 10+ | objective |
| Legg et al., 2005 | DHRO | EAU |  | in-person group | Professional | <5 | objective |
| Libert et al., 2022 | DHROF | C |  | in-person group | Researcher | 5 to 10 | objective |
| Liénard et al., 2010 | DHRF | C |  | in-person group | Professional | 5 to 10 | objective |
| Liu et al., 2016 | HRF | C |  | online/ind. | Other | <5 | objective |
| LoSasso et al., 2017 | HRO | AC |  | in-person group | Professional | <5 | other-report |
| Lozano, et al., 2010 | DHOF | C |  | in-person group | Researcher | <5 | objective |
| Maatouk-Bürmann et al., 2016 | DHF | C |  | in-person group | Professional | 10+ | objective |
| Maguire et al., 1986 | DHROF | C |  | in-person group | Professional | <5 | objective |
| Malhotra et al., 2019 | DROF | C |  | online/ind. | Other | <5 | objective |
| Marsh et al., 2021 | DHRF | EAU |  | in-person group + indiv.. | Professional | 5 to 10 | other-report |
| Marteau et al., 1991 | HOF | C |  | in-person group | Professional | 5 to 10 | objective |
| Matharu et al., 2014 | HRO | EAU |  | in-person group | Researcher | <5 | self-report |
| Merckaert et al., 2008; Razavi et al., 2003 ^b^ | DHRF | EAU |  | in-person group | Professional | 5 to 10 | objective |
| Moral et al., 2001 | HOF | C |  | in-person group | Professional | NR | objective |
| Moreland, 1971 | DHROF | AC |  | in-person group | Professional | 5 to 10 | objective |
| Moulton et al., 2009 | F | EAU |  | in-person ind. | Para-Professional | <5 | objective |
| Nayiga et al., 2014 | DRF | AC |  | in-person group | Professional | 5 to 10 | objective |
| Nikendei et al., 2011 | DHF | C |  | in-person group | Professional | <5 | objective |
| Patel et al., 2019 | DRO | EAU |  | in-person group | Professional | <5 | other-report |
| Perera et al., 2010 | DHRF | EAU |  | in-person group | Professional | <5 | objective |
| Pirdehghan et al., 2018 | DH | C |  | in-person group | Professional | <5 | objective |
| Pollak et al., 2016 | DO | C |  | online/ind. | Other | <5 | objective |
| Potash et al., 2014 | HRO | AC |  | in-person group | Professional | <5 | self-report |
| Price et al., 2008 | DHROF | C |  | in-person group | Professional | 5 to 10 | mixed |
| Qureshi & Zehra, 2020 | F | C |  | in-person ind. | Para-professional | <5 | objective |
| Rassbach et al., 2018 | ROF | EAU |  | in-person ind. | Professional | <5 | other-report |
| Reed, 1996 | DHRF | AC |  | in-person group | Professional | 5 to 10 | objective |
| Robins & Wolf, 1989 ^a^ | DHO | C |  | in-person ind. | Professional | NR | objective |
| Roche et al., 1997 | DRF | EAU |  | in-person group | Professional | <5 | objective |
| Roter et al., 1990 | DHROF | C |  | in-person ind. | Professional | NR | other-report |
| Sanson‐Fisher & Poole, 1978 | DR | C |  | in-person group | Para-Professional | 5 to 10 | objective |
| Schouten et al., 2005 | DHRF | C |  | in-person group | Professional | <5 | objective |
| Sepucha et al., 2022 | DHF | EAU |  | online/ind. | Other | <5 | other-report |
| Servotte et al., 2019 | DHO | EAU |  | in-person group | Professional | <5 | objective |
| Shapiro et al., 2009 | HF | C |  | in-person ind. | Professional | 10+ | objective |
| Smith et al., 1005 | DHROF | C |  | in-person group | Professional | 10+ | other-report |
| Stewart et al., 2007 | DHROF | EAU |  | in-person group | Professional | <5 | objective |
| Strohbehn et al., 2020 | HR^c^ | EAU |  | in-person group | Professional | <5 | self-report |
| Sullivan et al., 2010 | DO | EAU |  | online/ind. | Other | NR | self-report |
| Szmuilowicz et al., 2010 | DHROF | C |  | in-person group | Professional | <5 | objective |
| Szmuilowicz et al., 2012 | DHRO | AC |  | in-person group | Professional | <5 | objective |
| Tavakoly Sany et al., 2020 | DHR | EAU |  | in-person group | Professional | 5 to 10 | other-report |
| Tulsky et al., 2011 | DOF | AC |  | in-person group + indiv. | Researcher | <5 | mixed |
| Vincent et al., 2022 | DHO | EAU |  | online/ind. | Other | <5 | objective |
| Wolf et al., 1987 | DHROF | EAU |  | in-person group | Professional | <5 | objective |
| Wong et al., 2007 | DHROF | C |  | in-person group | Professional | 10+ | objective |
| Wuensch et al., 2017 | DHRF | C |  | in-person group + indiv. | Professional | <5 | objective |
| Wündrich et al., 2017 | DHROF | AC |  | in-person group | Professional | <5 | objective |
| Yu et al., 2018 | DHRF | AC |  | in-person group | Professional | NR | other-report |

Abbreviation*:*  C = Control, AC = Active Control, EAU = Education As Usual, D =Didactic, H =Rehearsal, O = Observation, R =Reflection, F=Feedback. Ind. = independent, Indiv. = individual, NR = not reported.

^a^This article reported on independent samples within, so were treated as separate studies in the analysis.

^b^These two articles shared the same sample.

^c^This study had two arms which were functionally equivalent and therefore combined as one arm.

## **Table 6.3.** Risk of Bias by Study

| Study | Random Sequence Generation | Allocation Concealment | Blinding of Participants or Personnel | Blinding of Outcome Assessment | Incomplete Outcome Data | Selective Reporting | Other | Risk of Bias^c^ |
| --- | --- | --- | --- | --- | --- | --- | --- | --- |
| Abbasi et al., 2018 | unclear | unclear | unclear | unclear | low | unclear | low | HIGH RISK |
| Alder et al., 2007 | unclear | unclear | unclear | low | unclear | unclear | high | HIGH RISK |
| Annadurai et al., 2021 | unclear | unclear | unclear | low | low | unclear | low | HIGH RISK |
| Bashour et al., 2013 | low | unclear | low | low | unclear | low | low | LOW RISK |
| Beach et al., 2015 | low | unclear | unclear | unclear | low | unclear | low | HIGH RISK |
| Bearman et al., 2001 | high | unclear | high | low | low | unclear | low | HIGH RISK |
| Bibl et al., 2021 | low | low | unclear | low | low | unclear | low | LOW RISK |
| Bickell et al., 2020 | unclear | unclear | high | low | low | unclear | low | HIGH RISK |
| Blatt et al., 2010 ^a^ | low | unclear | high | low | low | unclear | low | LOW RISK |
| Blödt et al., 2016 | low | low | low | low | low | low | low | LOW RISK |
| Bonvicini, 2007 | low | unclear | unclear | unclear | low | unclear | low | HIGH RISK |
| Bosse et al., 2012 | unclear | high | unclear | unclear | low | unclear | low | HIGH RISK |
| Bowyer et al., 2010 | unclear | unclear | low | low | low | unclear | low | LOW RISK |
| Brinkman et al., 2007 | low | unclear | high | low | low | unclear | low | LOW RISK |
| Brown et al., 1999 | low | unclear | unclear | unclear | unclear | unclear | low | HIGH RISK |
| Brown et al., 2018 | unclear | unclear | unclear | low | high | unclear | low | HIGH RISK |
| Butow et al., 2008 | low | unclear | unclear | unclear | low | unclear | low | HIGH RISK |
| Butow et al., 2015 | unclear | unclear | high | unclear | unclear | unclear | low | HIGH RISK |
| Cave et al., 2007 | unclear | high | unclear | unclear | low | unclear | low | HIGH RISK |
| Cooper et al., 2011 | unclear | low | unclear | unclear | low | low | low | LOW RISK |
| Coret et al., 2018 | unclear | low | low | low | low | unclear | low | LOW RISK |
| D’souza et al,. 2019 | low | low | high | high | low | unclear | high | HIGH RISK |
| Daeppen et al., 2012 | low | unclear | low | Low | low | unclear | Low | LOW RISK |
| Daetwyler et al., 2010 | high | low | high | unclear | unclear | unclear | low | HIGH RISK |
| Dominick et al., 2018 | unclear | unclear | unclear | unclear | low | unclear | low | HIGH RISK |
| Downar et al., 2017 | unclear | unclear | low | high | high | low | low | HIGH RISK |
| du Vaure et al., 2017 | low | unclear | high | low | unclear | low | low | LOW RISK |
| Dubosh et al., 2019 | high | unclear | high | low | low | unclear | low | HIGH RISK |
| Duran et al., 2020 | unclear | unclear | high | unclear | low | unclear | low | HIGH RISK |
| Dwyer et al., 2019 | low | low | unclear | low | low | low | low | LOW RISK |
| Epinat-Duclos et al., 2021 | unclear | unclear | high | low | high | unclear | low | HIGH RISK |
| Farrell et al., 2014 | low | unclear | low | low | low | unclear | low | LOW RISK |
| Flocke et al., 2014 | unclear | low | unclear | low | low | unclear | unclear | HIGH RISK |
| Foster et al., 2015 | unclear | unclear | low | low | Low | unclear | low | LOW RISK |
| Foster et al., 2016 | unclear | unclear | unclear | low | low | unclear | Low | HIGH RISK |
| Fujimori et al., 2014 | unclear | unclear | low | low | low | unclear | low | LOW RISK |
| Geoffroy et al., 2020 | unclear | unclear | low | low | low | unclear | low | LOW RISK |
| Giordani, 2002 | unclear | unclear | low | low | low | unclear | low | LOW RISK |
| Goelz et al., 2011 | low | unclear | low | low | low | unclear | low | LOW RISK |
| Green & Levi, 2011 | low | unclear | high | high | low | unclear | low | HIGH RISK |
| Grossman et al., 2021 | unclear | low | low | low | low | unclear | low | LOW RISK |
| Helitzer et al., 2011 | unclear | unclear | high | unclear | low | unclear | low | HIGH RISK |
| Henry et al., 2022 | unclear | low | low | low | low | unclear | low | LOW RISK |
| Henselmans et al., 2019 | low | low | unclear | low | low | unclear | low | LOW RISK |
| Henselmans et al., 2020 | low | low | high | unclear | low | unclear | low | LOW RISK |
| Herrmann-Werner et al., 2019 | unclear | unclear | unclear | low | low | unclear | low | HIGH RISK |
| Higgins et al., 1990 | unclear | unclear | low | low | unclear | unclear | low | HIGH RISK |
| Hilgenberg et al., 2018 | low | unclear | low | low | low | unclear | low | LOW RISK |
| Ho et al., 2008 | unclear | unclear | unclear | low | low | unclear | low | HIGH RISK |
| Hobgood et al., 2009 | low | low | high | unclear | unclear | unclear | low | HIGH RISK |
| Hobma et al., 2006 | low | unclear | low | high | low | unclear | low | LOW RISK |
| Hojat et al., 2013 | high | unclear | unclear | low | unclear | unclear | low | HIGH RISK |
| Intrieri et al., 1993 | unclear | unclear | low | low | high | unclear | low | HIGH RISK |
| Jenkins & Fallowfield, 2002 | unclear | unclear | high | low | low | unclear | low | HIGH RISK |
| Kaltman et al., 2018 | unclear | unclear | unclear | low | low | unclear | low | HIGH RISK |
| Karkowsky et al., 2016 | unclear | low | unclear | low | high | unclear | high | HIGH RISK |
| Kron et al., 2017 | unclear | unclear | unclear | low | low | unclear | Low | HIGH RISK |
| Lai et al., 2020 | low | low | low | unclear | low | low | low | LOW RISK |
| Langewitz et al., 1998 | unclear | unclear | unclear | low | low | unclear | low | HIGH RISK |
| Langewitz et al., 2003 | unclear | unclear | high | low | low | high | low | HIGH RISK |
| Legg et al., 2005 | unclear | unclear | unclear | low | low | unclear | low | HIGH RISK |
| Libert et al., 2022 | unclear | unclear | low | unclear | low | unclear | low | HIGH RISK |
| Liénard et al., 2010 | low | unclear | unclear | low | low | unclear | low | LOW RISK |
| Liu et al., 2016 | low | low | low | low | high | unclear | low | LOW RISK |
| LoSasso et al., 2017 | unclear | unclear | unclear | low | low | unclear | low | HIGH RISK |
| Lozano, et al., 2010 | low | unclear | low | low | low | unclear | low | LOW RISK |
| Maatouk-Bürmann et al., 2016 | unclear | unclear | low | low | low | unclear | low | LOW RISK |
| Maguire et al., 1986 | low | unclear | unclear | low | high | unclear | low | HIGH RISK |
| Malhotra et al., 2019 | unclear | unclear | unclear | low | low | unclear | low | HIGH RISK |
| Marsh et al., 2021 | low | unclear | unclear | unclear | low | unclear | low | HIGH RISK |
| Marteau et al., 1991 | unclear | unclear | high | low | low | unclear | low | HIGH RISK |
| Matharu et al., 2014 | low | unclear | unclear | low | low | unclear | low | LOW RISK |
| Merckaert et al., 2008; Razavi et al., 2003 ^b^ | unclear | unclear | unclear | low | low | unclear | low | HIGH RISK |
| Moral et al., 2001 | unclear | unclear | low | low | low | unclear | low | LOW RISK |
| Moreland, 1971 | unclear | unclear | high | low | low | low | low | LOW RISK |
| Moulton et al., 2009 | unclear | unclear | low | low | unclear | unclear | low | HIGH RISK |
| Nayiga et al., 2014 | unclear | unclear | high | high | low | unclear | low | HIGH RISK |
| Nikendei et al., 2011 | unclear | unclear | unclear | low | low | unclear | low | HIGH RISK |
| Patel et al., 2019 | low | unclear | low | low | high | low | low | LOW RISK |
| Perera et al., 2010 | unclear | unclear | low | low | unclear | unclear | low | HIGH RISK |
| Pirdehghan et al., 2018 | low | unclear | unclear | low | low | unclear | low | LOW RISK |
| Pollak et al., 2016 | unclear | unclear | high | low | low | low | high | HIGH RISK |
| Potash et al., 2014 | unclear | unclear | high | low | high | unclear | low | HIGH RISK |
| Price et al., 2008 | unclear | unclear | unclear | low | high | unclear | low | HIGH RISK |
| Qureshi & Zehra, 2020 | low | low | unclear | high | low | unclear | low | LOW RISK |
| Rassbach et al., 2018 | unclear | unclear | unclear | low | low | unclear | high | HIGH RISK |
| Reed, 1996 | low | unclear | low | low | low | unclear | low | LOW RISK |
| Robins & Wolf, 1989 ^a^ | unclear | unclear | low | low | low | unclear | low | LOW RISK |
| Roche et al., 1997 | high | unclear | high | low | low | unclear | low | HIGH RISK |
| Roter et al., 1990 | high | unclear | low | unclear | low | high | high | HIGH RISK |
| Sanson‐Fisher & Poole, 1978 | unclear | unclear | unclear | unclear | low | unclear | Low | HIGH RISK |
| Schouten et al., 2005 | unclear | unclear | high | low | low | unclear | low | HIGH RISK |
| Sepucha et al., 2022 | low | low | low | low | low | unclear | low | LOW RISK |
| Servotte et al., 2019 | unclear | unclear | unclear | low | low | unclear | low | HIGH RISK |
| Shapiro et al., 2009 | unclear | unclear | high | low | low | unclear | low | HIGH RISK |
| Smith et al., 1005 | unclear | unclear | high | unclear | unclear | high | high | HIGH RISK |
| Stewart et al., 2007 | low | unclear | high | low | high | unclear | low | HIGH RISK |
| Strohbehn et al., 2020 | low | High | high | low | low | unclear | low | LOW RISK |
| Sullivan et al., 2010 | unclear | unclear | low | low | high | unclear | low | HIGH RISK |
| Szmuilowicz et al., 2010 | low | unclear | low | low | low | unclear | low | LOW RISK |
| Szmuilowicz et al., 2012 | unclear | unclear | unclear | low | low | unclear | low | HIGH RISK |
| Tavakoly Sany et al., 2020 | low | low | high | low | low | unclear | low | LOW RISK |
| Tulsky et al., 2011 | low | unclear | unclear | low | low | unclear | low | LOW RISK |
| Vincent et al., 2022 | low | low | low | low | low | unclear | low | LOW RISK |
| Wolf et al., 1987 | unclear | unclear | high | low | high | unclear | low | HIGH RISK |
| Wong et al., 2007 | unclear | unclear | low | low | low | unclear | low | LOW RISK |
| Wuensch et al., 2017 | low | unclear | low | low | low | unclear | low | LOW RISK |
| Wündrich et al., 2017 | unclear | unclear | unclear | low | low | unclear | low | HIGH RISK |
| Yu et al., 2018 | unclear | unclear | low | low | low | unclear | low | LOW RISK |

^a^ This article reported on independent samples within, so were treated as separate studies in the analysis.

^b^ These two articles shared the same sample.

^c^ Studies were deemed “low risk” if four or more of the seven Cochrane categories for risk of bias assessment were determined to be low risk and “high risk” if four or more of the seven Cochrane categories for risk of bias assessment were determined to be high or unclear risk.

## **Table 6.****4.** Count and Percentage of Studies with Low, Unclear, and High Risk of Bias

|  | Random Sequence Generation | Allocation Concealment | Blinding of Participants or Personnel | Blinding of Outcome Assessment | Incomplete Outcome Data | Selective Reporting | Other |
| --- | --- | --- | --- | --- | --- | --- | --- |
| High Risk | 6 (5.5%) | 3 (2.8%) | 30 (27.5%) | 6 (5.5%) | 13 (11.9%) | 3 (2.8%) | 7 (6.4%) |
| Unclear Risk | 63 (57.8%) | 86 (78.9%) | 42 (38.5%) | 21 (19.3%) | 12(11.0%) | 96 (88.1%) | 1 (0.9%) |
| Low Risk | 40 (36.7%) | 20 (18.3%) | 37 (33.9%) | 82 (75.2%) | 84 (77.1%) | 10 (9.2%) | 101 (92.7%) |

## **Table 6.5.** Study Effect Sizes by Comparison Arm

| Study | Arm 1 | *n* | Arm 2 | *n* | Effect size (*d*) | Standard Error |
| --- | --- | --- | --- | --- | --- | --- |
| Abbasi et al., 2018 | D | 33 | C | 35 | 1.04 | 0.26 |
| Alder et al., 2007 | DHROF | 16 | C | 16 | 0.26 | 0.36 |
| Annadurai et al., 2021 | DHOF | 11 | EAU | 11 | 0.66 | 0.44 |
| Bashour et al., 2013 | DHR | 137 | C | 137 | 0.01 | 0.14 |
| Beach et al., 2015 | DO | 13 | C | 13 | 1.47 | 0.44 |
| Bearman et al., 2001^e^ | H | 38-41 | C | 55 | 0.08 | 0.21 |
| Bibl et al., 2021 | DHOF | 9 | C | 8 | -0.38 | 0.49 |
| Bickell et al., 2020 | DHROF | 11 | EAU | 11 | 0.85 | 0.70 |
| Blatt et al., 2010a^a^ | DHR | 122 | AC | 123 | 0.16 | 0.13 |
| Blatt et al., 2010b^a^ | DHR | 52 | AC | 53 | 0.31 | 0.20 |
| Blatt et al., 2010c^a^ | DHR | 135 | AC | 123 | 0.13 | 0.12 |
| Blödt et al., 2016 | DHROF | 8 | C | 9 | 0.10 | 0.49 |
| Bonvicini, 2007; Haskard et al., 2008^b^ | DHF | 58 | C | 58 | 1.16 | 0.21 |
| Bosse et al., 2012 | DHRF | 35 | AC | 34 | 1.06 | 0.22 |
| Bowyer et al., 2010^c^ | DRO | 227 | C | 163 | 0.43 | 0.10 |
|  | DRO | 227 | O | 163 | 0.10 | 0.10 |
|  | O | 163 | C | 163 | 0.35 | 0.11 |
| Brinkman et al., 2007 | DHRF | 17 | AC | 18 | 0.23 | 0.42 |
| Brown et al., 1999 | DH | 32 | C | 29 | -0.09 | 0.26 |
| Brown et al., 2018 | DHRO | 133 | EAU | 115 | 0.16 | 0.13 |
| Butow et al., 2008 | DHROF | 16 | C | 14 | 0.12 | 0.37 |
| Butow et al., 2015^e^ | DHROF | 10-21 | C | 11-20 | 0.17 | 0.38 |
| Cave et al., 2007^c^ | HF | 107 | EAU | 124 | 0.07 | 0.13 |
|  | DRF | 128 | HF | 107 | 0.09 | 0.13 |
|  | DRF | 128 | EAU | 124 | 0.16 | 0.13 |
| Cooper et al., 2011 | DHF | 22 | EAU | 19 | -0.76 | 0.32 |
| Coret et al., 2018 | DHRO | 15 | EAU | 12 | 0.35 | 0.39 |
| D’souza et al,. 2019 | DHRO | 33 | C | 38 | 0.30 | 0.24 |
| Daeppen et al., 2012 | DHRO | 42 | EAU | 49 | 0.91 | 0.22 |
| Daetwyler et al., 2010^c^ | DHOF | 16 | C | 19 | 0.61 | 0.35 |
|  | DHOF | 16 | DO | 17 | 0.28 | 0.35 |
|  | DO | 17 | C | 19 | 0.42 | 0.34 |
| Dominick et al., 2018 | HO | 38 | EAU | 30 | 0.11 | 0.30 |
| Downar et al., 2017 | DHROF | 52 | AC | 42 | -0.06 | 0.21 |
| du Vaure et al., 2017 | H | 155 | EAU | 144 | 0.08 | 0.12 |
| Dubosh et al., 2019 | DF | 27 | EAU | 37 | 0.19 | 0.25 |
| Duran et al., 2020 | DHROF | 14 | EAU | 12 | -0.14 | 0.39 |
| Dwyer et al., 2019 | DRO | 26 | C | 22 | -0.22 | 0.29 |
| Epinat-Duclos et al., 2021 | DHR | 66 | AC | 87 | 0.11 | 0.16 |
| Farrell et al., 2014 | DHF | 46 | EAU | 46 | 0.33 | 0.21 |
| Flocke et al., 2014 | DHOF | 16 | AC | 15 | 0.59 | 0.37 |
| Foster et al., 2015^c^ | DHF | 35 | AC | 17 | 0.89 | 0.31 |
|  | DHF | 35 | H | 18 | 1.39 | 0.32 |
|  | H | 18 | AC | 17 | 0.27 | 0.25 |
| Foster et al., 2016 | DO | 18 | AC | 17 | -0.60 | 0.35 |
| Fujimori et al., 2014 | DHROF | 15 | C | 15 | 1.30 | 0.40 |
| Geoffroy et al., 2020 | HOF | 64 | EAU | 72 | 0.48 | 0.17 |
| Giordani, 2002 | DOF | 75 | C | 75 | 0.15 | 0.16 |
| Goelz et al., 2011 | DHF | 22 | C | 19 | 0.95 | 0.33 |
| Green & Levi, 2011 | H | 60 | EAU | 61 | 0.14 | 0.18 |
| Grossman et al., 2021 | HROF | 72 | EAU | 116 | 0.55 | 0.15 |
| Helitzer et al., 2011^e^ | DHROF | 10-12 | C | 13-14 | 1.13 | 0.44 |
| Henry et al., 2022 | DHF | 21 | EAU | 16 | 0.27 | 0.33 |
| Henselmans et al., 2019 | DHOF | 15 | EAU | 16 | 1.75 | 0.43 |
| Henselmans et al., 2020 | DHOF | 13 | EAU | 13 | 1.41 | 0.44 |
| Herrmann-Werner et al., 2019 | DHROF | 26 | EAU | 20 | 0.70 | 0.31 |
| Higgins et al., 1990^d^ | DHROF | 7 | C | 6 | 2.88 | 0.79 |
| Hilgenberg et al., 2018 | DHRO | 43 | DR | 41 | 0.48 | 0.22 |
| Ho et al., 2008 ^c^ | DH | 15 | C | 27 | 1.34 | 0.36 |
|  | DH | 15 | D | 15 | 1.31 | 0.41 |
|  | D | 15 | C | 27 | 0.02 | 0.32 |
| Hobgood et al., 2009 | DHRF | 35 | DHR | 103 | 0.35 | 0.20 |
| Hobma et al., 2006 | DF | 38 | AC | 38 | 0.26 | 0.23 |
| Hojat et al., 2013 ^c^ | DR | 55 | AC | 69 | 0.16 | 0.18 |
|  | DR | 55 | R | 38 | -0.02 | 0.21 |
|  | R | 38 | AC | 69 | 0.18 | 0.20 |
| Intrieri et al., 1993 | DHR | 34 | C | 50 | 0.43 | 0.22 |
| Jenkins & Fallowfield, 2002 | DHROF | 48 | C | 45 | 0.41 | 0.23 |
| Kaltman et al., 2018 | H | 60 | EAU | 39 | 0.13 | 0.21 |
| Karkowsky et al., 2016 | DRF | 17 | EAU | 18 | 0.34 | 0.34 |
| Kron et al., 2017 | HR | 210 | AC | 211 | 0.27 | 0.10 |
| Lai et al., 2020 | DRF | 35 | F | 35 | 0.74 | 0.25 |
| Langewitz et al., 1998 | DHOF | 19 | EAU | 23 | 1.12 | 0.33 |
| Langewitz et al., 2003 | DHRF | 19 | EAU | 23 | 0.30 | 0.35 |
| Legg et al., 2005 | DHRO | 11 | EAU | 10 | 1.09 | 0.47 |
| Libert et al., 2022 | DHROF | 42 | C | 45 | 1.48 | 0.25 |
| Liénard et al., 2010 | DHRF | 50 | C | 48 | 0.13 | 0.20 |
| Liu et al., 2016 | HRF | 59 | C | 107 | 0.18 | 0.16 |
| LoSasso et al., 2017 | HRO | 38 | AC | 32 | 0.36 | 0.24 |
| Lozano, et al., 2010 | DHOF | 8 | C | 8 | 0.38 | 0.59 |
| Maatouk-Bürmann et al., 2016 | DHF | 18 | C | 21 | -0.01 | 0.32 |
| Maguire et al., 1986 | DHROF | 16 | C | 16 | 0.87 | 0.37 |
| Malhotra et al., 2019 | DROF | 5 | C | 5 | 0.33 | 0.64 |
| Marsh et al., 2021 | DHRF | 19 | EAU | 17 | 0.04 | 0.51 |
| Marteau et al., 1991 | HOF | 28 | C | 45 | -0.03 | 0.24 |
| Matharu et al., 2014 | HRO | 63 | EAU | 66 | 0.11 | 0.18 |
| Merckaert et al., 2008; Razavi et al., 2003^b,e^ | DHRF | 27-29 | EAU | 29-33 | 0.35 | 0.26 |
| Moral et al., 2001 | HOF | 10 | C | 10 | 1.32 | 0.49 |
| Moreland, 1971^d^ | DHROF | 12 | AC | 12 | 0.92 | 0.43 |
| Moulton et al., 2009 | F | 16 | EAU | 16 | 0.87 | 0.37 |
| Nayiga et al., 2014 | DRF | 14 | AC | 10 | -0.09 | 0.41 |
| Nikendei et al., 2011 | DHF | 14 | C | 14 | 0.56 | 0.39 |
| Patel et al., 2019 | DRO | 44 | EAU | 32 | 0.23 | 0.23 |
| Perera et al., 2010 | DHRF | 97 | EAU | 93 | 0.58 | 0.15 |
| Pirdehghan et al., 2018 | DH | 30 | C | 30 | 9.63 | 0.92 |
| Pollak et al., 2016 | DO | 22 | C | 24 | 0.71 | 0.30 |
| Potash et al., 2014 | HRO | 48 | AC | 58 | -0.30 | 0.20 |
| Price et al., 2008^e^ | DHROF | 51-59 | C | 46-61 | 0.31 | 0.20 |
| Qureshi & Zehra, 2020 | F | 80 | C | 80 | 0.77 | 0.17 |
| Rassbach et al., 2018 | ROF | 57 | EAU | 57 | -0.07 | 0.25 |
| Reed, 1996^d^ | DHRF | 12 | AC | 11 | 2.02 | 0.51 |
| Robins & Wolf, 1989a^a^ | DHO | 48 | C | 46 | 0.59 | 0.56 |
| Robins & Wolf, 1989b^a^ | DHO | 50 | C | 49 | 0.84 | 0.31 |
| Roche et al., 1997 | DRF | 56 | EAU | 55 | 0.14 | 0.19 |
| Roter et al., 1990 | DHROF | 24 | C | 24 | 0.76 | 0.30 |
| Sanson‐Fisher & Poole, 1978 | DR | 112 | C | 23 | 4.36 | 0.35 |
| Schouten et al., 2005^e^ | DHRF | 15-19 | C | 13-18 | 0.05 | 1.35 |
| Sepucha et al., 2022 | DHF | 28 | EAU | 31 | 0.33 | 0.26 |
| Servotte et al., 2019 | DHO | 37 | EAU | 31 | 0.62 | 0.25 |
| Shapiro et al., 2009 | HF | 38 | C | 41 | 0.33 | 0.23 |
| Smith et al., 1005 | DHROF | 14 | C | 12 | 0.18 | 0.39 |
| Stewart et al., 2007 | DHROF | 8 | EAU | 9 | 1.03 | 0.52 |
| Strohbehn et al., 2020 | HR | 21 | EAU | 13 | 0.25 | 0.35 |
| Sullivan et al., 2010 | DO | 88 | EAU | 85 | -0.20 | 0.15 |
| Szmuilowicz et al., 2010 | DHROF | 19 | C | 23 | 1.06 | 0.33 |
| Szmuilowicz et al., 2012 | DHRO | 19 | AC | 19 | 0.54 | 0.39 |
| Tavakoly Sany et al., 2020 | DHR | 17 | EAU | 18 | 1.29 | 0.37 |
| Tulsky et al., 2011 | DOF | 24 | AC | 24 | 0.64 | 0.30 |
| Vincent et al., 2022 | DHO | 80 | EAU | 80 | 0.09 | 0.16 |
| Wolf et al., 1987^e^ | DHROF | 56-64 | EAU | 53-69 | 0.77 | 0.19 |
| Wong et al., 2007 | DHROF | 16 | C | 16 | 0.56 | 0.36 |
| Wuensch et al., 2017 | DHRF | 20 | C | 20 | 0.51 | 0.32 |
| Wündrich et al., 2017 | DHROF | 79 | AC | 79 | 0.81 | 0.17 |
| Yu et al., 2018 | DHRF | 30 | AC | 30 | 0.33 | 0.26 |

Note. Unless otherwise specified, all data came from a primary reference. All results reported have been computed or estimated from other information. Abbreviation*: n* = sample size, *d* = Cohen’s *d* or standard mean difference, C = Control, AC = Active Control, EAU= Education As Usual, D =Didactic, H =Rehearsal, O = Observation, R =Reflection, F=Feedback

^a^This article reported on independent samples within, so were treated as separate studies in the analysis (*k* = 2)

^b^These two articles shared the same sample (*k* = 2).

^c^This article had three study arms (*k* = 6).

^d^This data came from a source of grey literature (*k* = 3).

^e^Ranges reflect sample size differences across outcome measures; CMA weighted each effect by its sample-specific variance (*k* = 7)

**Table 6.7.** Extracted Measurement Outcomes by Study

| Study | Measure Name | Assessment Type | Level |
| --- | --- | --- | --- |
| Abbasi et al., 2018 | Sheering Emotional Intelligence Inventory | self-report | subscale |
|  | Sheering Emotional Intelligence Inventory | self report | subscale |
| Alder et al., 2007 | Maastricht History-taking and Advice Checklist-Revised (MHAC-R) | objective | item |
|  | Maastricht History-taking and Advice Checklist-Revised (MHAC-R) | objective | item |
|  | Maastricht History-taking and Advice Checklist-Revised (MHAC-R) | objective | item |
| Annadurai et al., 2021 | Goal of Care (GoC) Meeting Assessment | objective | scale |
| Bashour et al., 2013 | Modified Interview Satisfaction scale | other-report | item |
|  | Modified Interview Satisfaction scale | other-report | item |
|  | Modified Interview Satisfaction scale | other-report | item |
|  | Modified Interview Satisfaction scale | other-report | item |
| Beach et al., 2015 | Roter Interaction Analysis System (RIAS) | objective | item |
| Bearman et al., 2001 | Communication Skills List | objective | item |
|  | Communication Skills List | objective | item |
|  | Communication Skills List | objective | item |
|  | Communication Skills List | objective | item |
|  | Communication Skills List | objective | item |
|  | Communication Skills List | objective | item |
| Bibl et al., 2021 | Objective Structured Clinical Examination (OSCE) | objective | scale |
| Blatt et al., 2010 | Patient Satisfaction Checklist | other-report | scale |
|  | Interpersonal Skills questionnaire | other-report | scale |
|  | Patient Satisfaction scale | other-report | scale |
| Blödt et al., 2016 | Assessment of Empathic Communication in Medical Interviews (REM) | objective | subscale |
| Bonvicini, 2007 | Empathic Communication Coding System (ECCS) | objective | scale |
| Bosse et al., 2012 | Calgary-Cambridge Observation Guide (CCOG) Checklist | objective | scale |
| Bowyer et al., 2010 | Standardized Patient Checklist | other-report | item |
|  | Standardized Patient Checklist | other-report | item |
|  | Standardized Patient Checklist | other-report | item |
|  | Standardized Patient Checklist | other-report | item |
|  | Standardized Patient Checklist | other-report | item |
|  | Standardized Patient Checklist | other-report | item |
|  | Standardized Patient Checklist | other-report | item |
| Brinkman et al., 2007 | Nurse Ratings of Residents | other-report | item |
|  | Nurse Ratings of Residents | other-report | item |
|  | Patient Ratings of Residents | other-report | item |
|  | Patient Ratings of Residents | other-report | item |
|  | Patient Ratings of Residents | other-report | item |
|  | Patient Ratings of Residents | other-report | item |
|  | Nurse Ratings of Residents | other-report | item |
|  | Nurse Ratings of Residents | other-report | item |
|  | Patient Ratings of Residents | other-report | item |
|  | Patient Ratings of Residents | other-report | item |
| Brown et al., 1999 | Art of Medicine Survey | other-report | scale |
| Brown et al., 2018 | Palliative Care (PC) Communication Questionnaire | self-report | item |
| Butow et al., 2008 | Key Doctor Behaviours | objective | subscale |
|  | Key Doctor Behaviours | objective | subscale |
|  | Key Doctor Behaviours | objective | subscale |
|  | Key Doctor Behaviours | objective | subscale |
|  | Key Doctor Behaviours | objective | subscale |
| Butow et al., 2015 | Decision Analysis Outcome System for Oncology | objective | subscale |
|  | Decision Analysis Outcome System for Oncology | objective | subscale |
| Cave et al., 2007 | Objective Structured Clinical Examination (OSCE) | objective | scale |
| Cooper et al., 2011 | Roter Interaction Analysis System (RIAS) | objective | scale |
| Coret et al., 2018 | Communication Skills Assessment - Objective Evaluator Form | objective | scale |
| D'souza et al., 2019 | Jefferson scale of Empathy - Student (JSE-S) | self-report | scale |
| Daeppen et al., 2012 | Motivational Interviewing Treatment Integrity scoring tool | objective | subscale |
| Daetwyler et al., 2010 | Breaking Bad News (BBN) Skills Checklist | other-report | scale |
| Dominick DeBlasio et al., 2018 | Communication Assessment Tool (CAT) | other-report | scale |
| Downar et al., 2017 | Consultation And Relational Empathy (CARE) Measure | objective | scale |
| du Vaure et al., 2017 | Consultation And Relational Empathy (CARE) Measure | other-report | scale |
| Dubosh et al., 2019 | Communication Assessment Tool (CAT) | other-report | scale |
| Duran et al., 2020 | Consultation and Relational Empathy (CARE) Measure | other-report | scale |
| Dwyer et al., 2019 | Interpersonal Reactivity Index | self-report | scale |
| Epinat-Duclos et al., 2021 | Jefferson Scale of Physician Empathy | self-report | scale |
|  | Privileged Knowledge | objective | subscale |
|  | Privileged Knowledge | objective | subscale |
|  | Empathy for Pain Evaluation | objective | item |
|  | Empathy for Pain Evaluation | objective | item |
| Farrell et al., 2014 | Communication Quality Indicator (Precautionary Empathy) | objective | subscale |
|  | Communication Quality Indicator (Precautionary Empathy) | objective | subscale |
|  | Communication Quality Indicator (Precautionary Empathy) | objective | subscale |
|  | Communication Quality Indicator (Precautionary Empathy) | objective | subscale |
|  | Communication Quality Indicator (Precautionary Empathy) | objective | subscale |
| Flocke et al., 2014 | Responding in Alignment | objective | scale |
| Foster et al., 2015 | Interview skills - Objective Structured Clinical Examination (OSCE) | objective | scale |
|  | Medical Student Interviewing Performance Questionnaire | other-report | subscale |
| Foster et al., 2016 | Empathic Communication Coding System (ECCS) | objective | subscale |
| Fujimori et al., 2014 | Objective Performance of Communication Skills | objective | subscale |
|  | Objective Performance of Communication Skills | objective | subscale |
| Geoffroy et al., 2020 | Modified Calgary Guide Interview | objective | scale |
| Giordani, 2002 | Interviewing Skills | objective | scale |
|  | Medical Interview Satisfaction scale | objective | scale |
|  | Global Interview Rating scale | objective | scale |
|  | Mutual Decision-Making | objective | scale |
| Goelz et al., 2011 | Communication in Oncology (COM-ON) Checklist | objective | scale |
| Green et al., 2011 | Patients’ Evaluation of the Student’s Performance | other-report | item |
|  | Patients’ Evaluation of the Student’s Performance | other-report | item |
|  | Patients’ Evaluation of the Student’s Performance | other-report | item |
|  | Patients’ Evaluation of the Student’s Performance | other-report | item |
|  | Patients’ Evaluation of the Student’s Performance | other-report | item |
| Grossman et al., 2021 | Empathetic Communication Coding System (ECCS) | objective | scale |
|  | Standardised Patient—Patient Satisfaction Ratings | other-report | subscale |
| Helitzer et al., 2011 | Roter Interaction Analysis System (RIAS) | objective | scale |
| Henry et al., 2022 | No. Target Clinician Communication Behaviors Observed | objective | scale |
| Henselmans et al., 2019 | Observing Patient Involvement scale | objective | scale |
|  | 4-stage Shared Decision Making (SDM) Model | objective | scale |
|  | Communication Skills | objective | item |
|  | Communication Skills | objective | item |
| Henselmans et al., 2020 | Observing Patient Involvement scale | objective | scale |
|  | Shared Decision Making (SDM) Questionnaire | objective | scale |
| Herrmann-Werner et al., 2019 | Jefferson scale of Patient Perception of Physician Empathy (JSPPPE) | other-report | scale |
| Higgins, 1990 | Carkhuff Empathy Rating scale | objective | scale |
| Hilgenberg et al., 2018 | Standardized Patient Assessment Tool | other-report | subscale |
|  | Fundamental Communication Skills | other-report | item |
|  | Fundamental Communication Skills | other-report | item |
|  | Fundamental Communication Skills | other-report | item |
|  | Fundamental Communication Skills | other-report | item |
|  | Fundamental Communication Skills | other-report | item |
| Ho et al., 2008 | Objective Structured Clinical Examination (OSCE) | objective | subscale |
|  | Objective Structured Clinical Examination (OSCE) | objective | subscale |
| Hobgood et al., 2009 | Communication Interpersonal Skills/Interpersonal Communication Skills | other-report | scale |
| Hobma et al., 2006 | Maastricht History-taking and Advice Scoring (MHAC) | objective | subscale |
|  | Maastricht History-taking and Advice Scoring (MHAC) | objective | subscale |
|  | Maastricht History-taking and Advice Scoring (MHAC) | objective | subscale |
|  | Maastricht History-taking and Advice Scoring (MHAC) | objective | subscale |
|  | Maastricht History-taking and Advice Scoring (MHAC) | objective | subscale |
|  | Maastricht History-taking and Advice Scoring (MHAC) | objective | subscale |
| Hojat et al., 2013 | Jefferson scale of Empathy (JSE) | self-report | scale |
| Intrieri et al., 1993 | Interview Rating | objective | subscale |
|  | Interview Rating | objective | subscale |
| Jenkins & Fallowfield, 2002 | Medical Interaction Process System | objective | item |
|  | Medical Interaction Process System | objective | item |
| Kaltman et al., 2018 | Objective Structured Clinical Examination (OSCE) Communication Behaviors - CASE 1 | objective | subscale |
|  | Objective Structured Clinical Examination (OSCE) Communication Behaviors - CASE 2 | objective | subscale |
|  | Objective Structured Clinical Examination (OSCE) Communication Behaviors - CASE 1 | objective | subscale |
|  | Objective Structured Clinical Examination (OSCE) Communication Behaviors - CASE 2 | objective | subscale |
| Karkowsky et al., 2016 | Objective Structured Clinical Exams (OSCE) Evaluation Form - Faculty | objective | scale |
| Kron et al., 2017 | Objective Structured Clinical Examination (OSCE) | Objective | scale |
| Lai et al., 2020 | Tutor-rated Clinical Skills | objective | subscale |
| Langewitz et al., 1998 | Maastricht History-taking and Advice Checklist-Revised (MHAC-R) | objective | subscale |
|  | Maastricht History-taking and Advice Checklist-Revised (MHAC-R) | objective | subscale |
| Langewitz et al., 2003 | Roter Interaction Analysis System (RIAS) | objective | subscale |
| Legg et al., 2005 | Modified Supported Conversation Analysis | objective | scale |
|  | Modified Supported Conversation Analysis | objective | scale |
|  | Modified Calgary Cambridge Observation Guide (CCOG-Modified) | objective | subscale |
|  | Modified Calgary Cambridge Observation Guide (CCOG-Modified) | objective | subscale |
|  | Modified Calgary Cambridge Observation Guide (CCOG-Modified) | objective | subscale |
| Libert et al., 2022 | Behavioral changes - LaCom content analysis | objective | item |
|  | Behavioral changes - LaCom content analysis | objective | item |
|  | Behavioral changes - LaCom content analysis | objective | item |
|  | Behavioral changes - LaCom content analysis | objective | item |
|  | Behavioral changes - LaCom content analysis | objective | item |
| Liénard et al., 2010 | LaComm Communication Content analysis | objective | subscale |
|  | LaComm Communication Content analysis | objective | subscale |
| Liu et al., 2016 | Student-Patient Observed Communication Assessment | objective | subscale |
|  | Student-Patient Observed Communication Assessment | objective | subscale |
| LoSasso et al., 2017 | Communication Skills Rating scale - Faculty | other-report | scale |
|  | Communication Skills Rating scale - Standardized Patient | other-report | scale |
|  | Jefferson scale of Patient Perceptions of Physician Empathy (JSPPPE) - Faculty | other-report | scale |
|  | Jefferson scale of Patient Perceptions of Physician Empathy (JSPPPE) - Standardized Patient | other-report | scale |
| Lozano et al., 2010 | Motivational Interviewing Treatment Integrity scale - "Not Ready" Station | objective | scale |
|  | Motivational Interviewing Treatment Integrity scale - "Unsure" Station | objective | scale |
|  | Motivational Interviewing Treatment Integrity scale - "Ready" Station | objective | scale |
| Maatouk-Bürmann et al., 2016 | Naming, Understanding, Respecting, Supporting, Exploring | objective | scale |
|  | Roter Interaction Analysis System (RIAS) | objective | subscale |
|  | Waiting, Echoing, Mirroring, and Summarizing | objective | scale |
| Maguire et al., 1986 | Main interview Skills | objective | item |
|  | Main interview Skills | objective | item |
|  | Main interview Skills | objective | item |
|  | Main interview Skills | objective | item |
|  | Main interview Skills | objective | item |
|  | Main interview Skills | objective | item |
| Malhotra et al., 2019 | Suchman's Empathic Communication Model | objective | subscale |
| Marsh et al., 2021 | Communication Assessment Tool (CAT) | other-report | scale |
| Marteau et al., 1991 | Interview Rating scale | objective | item |
|  | Interview Rating scale | objective | item |
|  | Interview Rating scale | objective | item |
|  | Interview Rating scale | objective | item |
| Matharu et al., 2014 | Jefferson scale of Physician Empathy (JSPE) | self-report | scale |
| Merckaert et al., 2008; Razavi et al., 2003^b^ | Physician's detection of patient's distress | objective | scale |
|  | Cancer Research Campaign Workshop Evaluation Manual - Simulated Patient | objective | item |
|  | Cancer Research Campaign Workshop Evaluation Manual - Simulated Patient | objective | item |
| Moral et al., 2001 | GATHARES-CP Questionnaire | objective | scale |
| Moreland, 1971 | Respect scale | objective | scale |
|  | Attending Behavior | objective | scale |
|  | Empathy scale | objective | scale |
|  | Relevant Concreteness scale | objective | scale |
|  | Genuineness scale | objective | scale |
|  | Confrontation scale | objective | scale |
| Moulton et al., 2009 | Therapist Verbalization Classification Sheet | objective | scale |
| Nayiga et al., 2014 | Respect scale | objective | scale |
| Nikendei et al., 2011 | Calgary–Cambridge Observation Guide (CCOG) Checklist | objective | subscale |
|  | Calgary–Cambridge Observation Guide (CCOG) Checklist | objective | subscale |
|  | Calgary–Cambridge Observation Guide (CCOG) Checklist | objective | subscale |
| Nina et al., 2020 | Empathy scale | objective | item |
|  | Relevant Concreteness scale | objective | item |
| Patel et al., 2019 | Genuineness scale | objective | scale |
| Perera et al., 2010 | Confrontation scale | objective | scale |
| PIrdenhghan et al., 2018 | Communication Skills Assessment Tool | objective | scale |
| Pollak et al., 2016 | Motivational Interviewing Treatment Integrity | objective | subscale |
| Potash et al., 2014 | Jefferson scale of Empathy - Student (JSE-S) | self-report | item |
| Price et al., 2008 | Roter Interaction Analysis System (RIAS) | objective | item |
|  | Roter Interaction Analysis System (RIAS) | objective | item |
|  | Standardized Patient Checklist | other-report | subscale |
| Qureshi et al., 2020 | Liverpool Communication skills Assessment scale - Station 1 counseling | objective | scale |
|  | Liverpool Communication skills Assessment scale - Station 2 informed consent | objective | scale |
|  | Liverpool Communication skills Assessment scale - Station 3 BBN | objective | scale |
|  | Liverpool Communication skills Assessment scale - Station 4 history taking | objective | scale |
| Rassbach et al., 2018 | Communication Assessment Tool (CAT) | other-report | scale |
| Reed, 1996  Robins & Wolf, 1989a | Truax and Carkhuff Accurate Empathy scale | objective | scale |
|  | Responses to Topics of Concern - Conversation Analysis (vignette 1) | objective | subscale |
| Reed, 1996 | Responses to Topics of Concern - Conversation Analysis (vignette 1) | objective | subscale |
| Robins & Wolf, 1989a  Robins & Wolf, 198b | Responses to Topics of Concern - Conversation Analysis (vignette 1) | objective | subscale |
|  | Responses to Topics of Concern - Conversation Analysis (vignette 1) | objective | subscale |
|  | Responses to Topics of Concern - Conversation Analysis (vignette 2) | objective | subscale |
|  | Responses to Topics of Concern - Conversation Analysis (vignette 2) | objective | subscale |
| Roche et al., 1997  Roter et al., 1990 | Rating Schedule | objective | subscale |
|  | Interaction Rating Form | other-report | scale |
| Sanson‐Fisher & Poole, 1978 | Traux and Carkhuff's Accurate Empathy scale | objective | scale |
| Schouten et al., 2005 | Objective Patient-Centred Communication Measure | objective | scale |
|  | Objective Patient-Centred Communication Measure | objective | scale |
|  | Objective Patient-Centred Communication Measure | objective | scale |
| Sepucha et al., 2022 | Shared Decision-Making (SDM) Process scale | other-report | scale |
| Servotte et al., 2019 | Breaking Bad News (BBN) Skills Assessment - Modified | objective | scale |
|  | Setting, Perception, Invitation, Knowledge, Empathy, Strategy (SPIKES) Competence Form | objective | scale |
| Shapiro et al., 2009 | Staff-Patient Interaction Rating scale | objective | scale |
| Smith et al., 1995 | Measure of Patient Satisfaction | other-report | subscale |
| Stewart et al., 2007 | Objective Patient-Centred Communication Measure | objective | scale |
| Strohbehn et al., 2020 | Jefferson scale of Physician Empathy - Students (JSPE-S) | self-report | scale |
| Sullivan et al., 2010 | Physician Patient-centeredness | self-report | scale |
| Szmuilowicz et al., 2010 | Interview Rating | objective | subscale |
| Szmuilowicz et al., 2012 | Code Status Discussion Checklist | objective | scale |
| Tavakoly Sany et al., 2020 | Health Literacy Assessment Questions | other-report | scale |
| Tulsky et al., 2011 | Model of Empathic Communication | objective | subscale |
|  | Model of Empathic Communication | objective | subscale |
|  | Perceived Empathy scale | other-report | scale |
| Vincent et al., 2022 | Breaking Bad News (BBN) | objective | scale |
| Wolf et al., 1987 | Medical Communication Index | objective | scale |
|  | Helping Relationship Inventory | objective | subscale |
| Wong et al., 2007 | Calgary-Cambridge Observation Guide (CCOG) | objective | scale |
| Wuensch et al., 2017 | Objective Performance of Communication Skills | objective | subscale |
|  | Objective Performance of Communication Skills | objective | subscale |
| Wündrich et al., 2017 | Objective Structured Clinical Exams (OSCE) Evaluation Form - Expert | objective | scale |
|  | Objective Structured Clinical Exams (OSCE) Evaluation Form - Standardized Patient | objective | scale |
| Yu et al., 201 | Communication Assessment Tool (CAT) | other-report | scale |

# Supplemental Appendix 7

# Publication Bias Testing

### **Figure 7.1.** Pairwise regression funnel plot


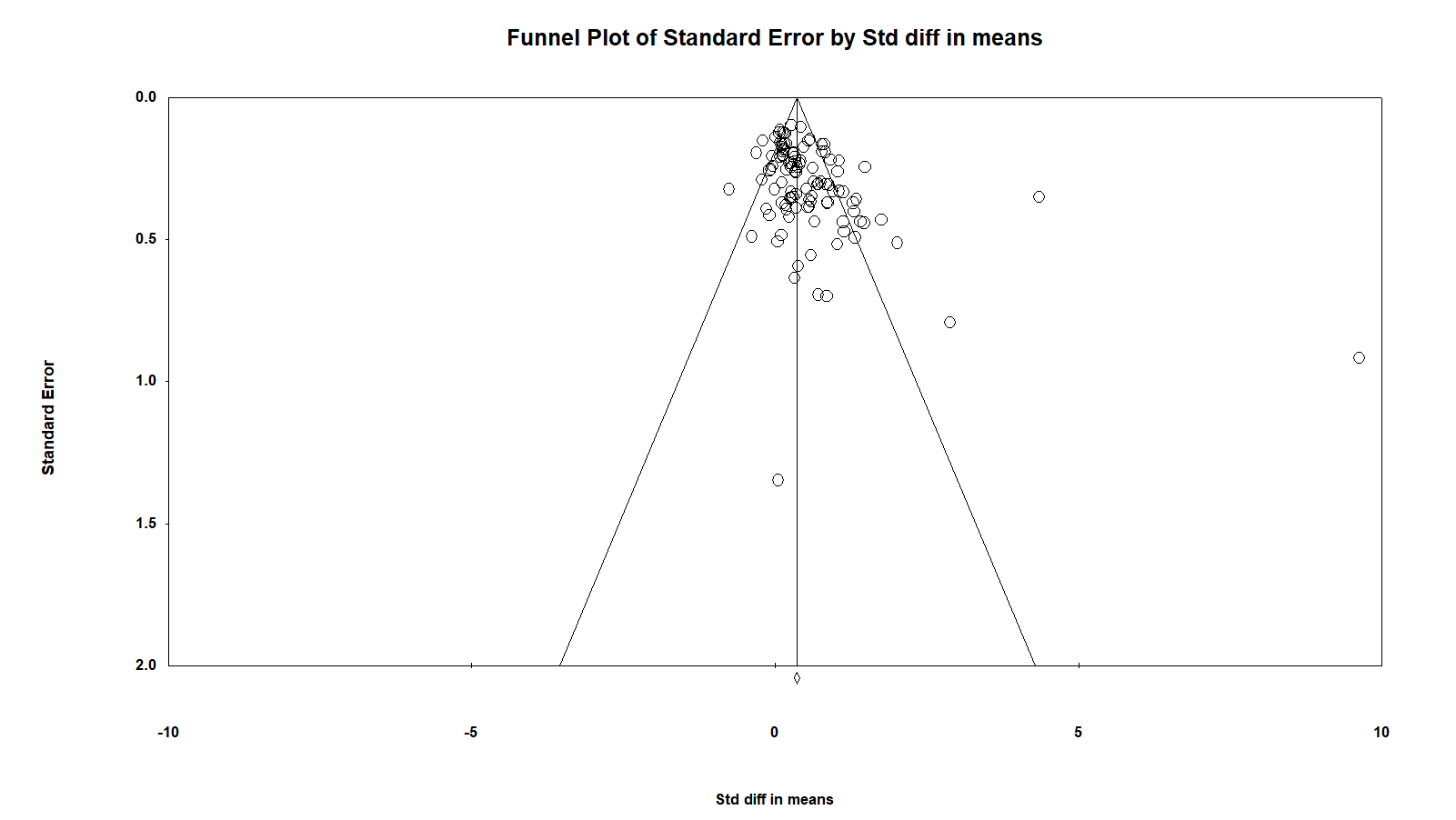


This figure plots a study’s effect size on the X axis with its standard error on the Y axis. A symmetrical pattern indicates an absence of publication bias whereas an asymmetrical pattern indicates presence of publication bias.

# Supplemental Appendix 8

# Network Meta-Analysis Results

### **Figure 8.1****.** SUCRA Curves


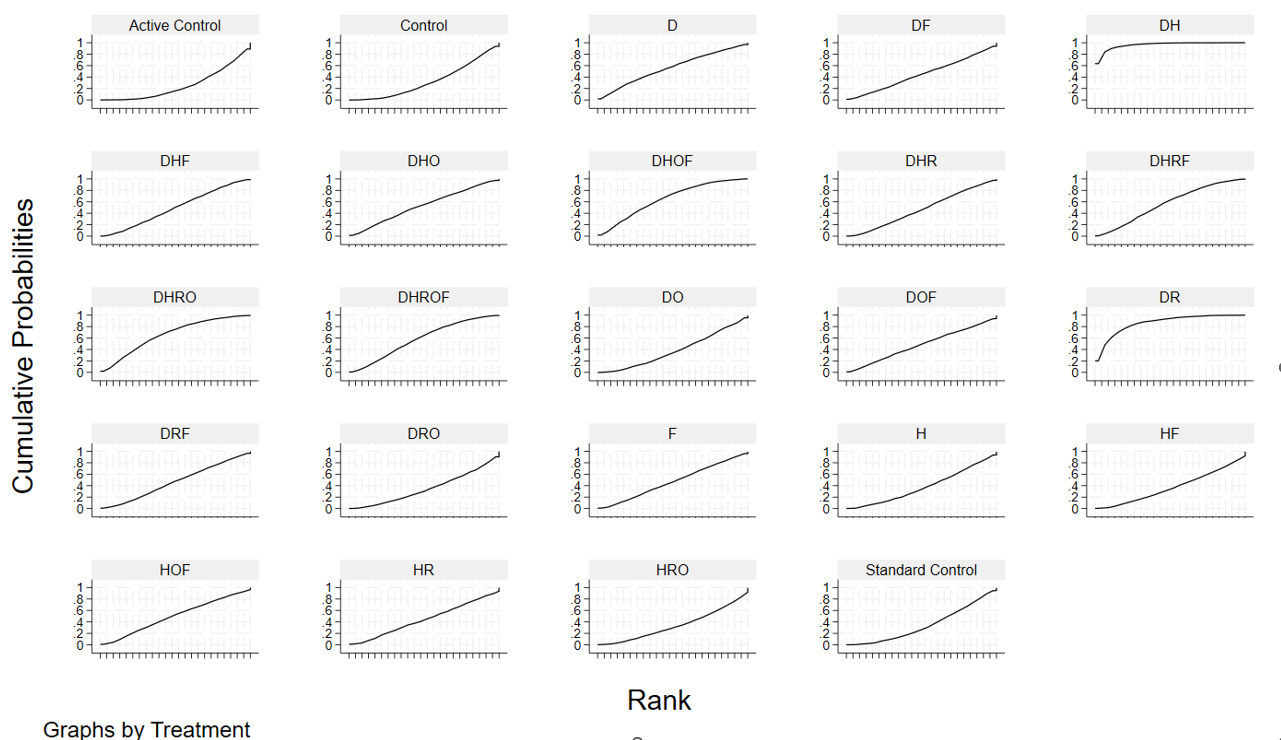


Abbreviations*:* D =Didactic, H =Rehearsal, O = Observation, R =Reflection, F=Feedback, Standard Control = Education As Usual. The X-axis values range from 1 to 24. Curves demonstrate the cumulative probability of each type of program being the most effect (rank 1), second most effective (rank =2), and so forth up to 24. Curves which have an earlier rise indicate greater chances of that program being among the best.

**Figure 8.2.** Network Forest Plot


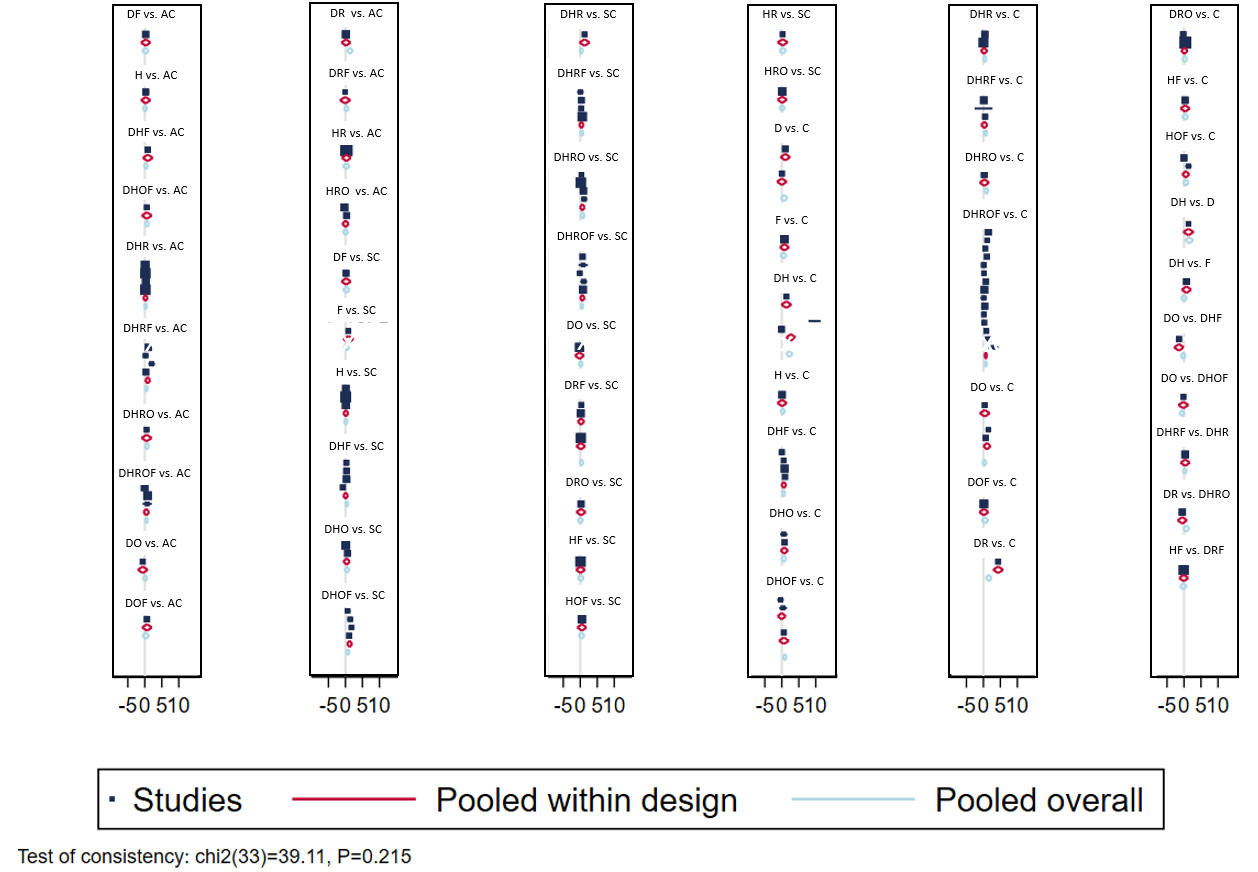


# Abbreviations: C = control, AC = Active Control, SC = Standard Care (“Education As Usual”), vs. = versus, D =Didactic, H =Rehearsal, O = Observation, R =Reflection, F=Feedback

Heterogeneity among individual studies within a treatment can be visually inspected. Moreover, similarity between the pooled effect of each treatment in the comparison set (“pooled within”, in red color) and the size of pooled overall effect (“pooler overall”, in blue colour) can be visually assessed. Similarity between these two effects indicates low heterogeneity and supports the consistency assumption

**Figure 8.3.** Comparison-adjusted funnel plot


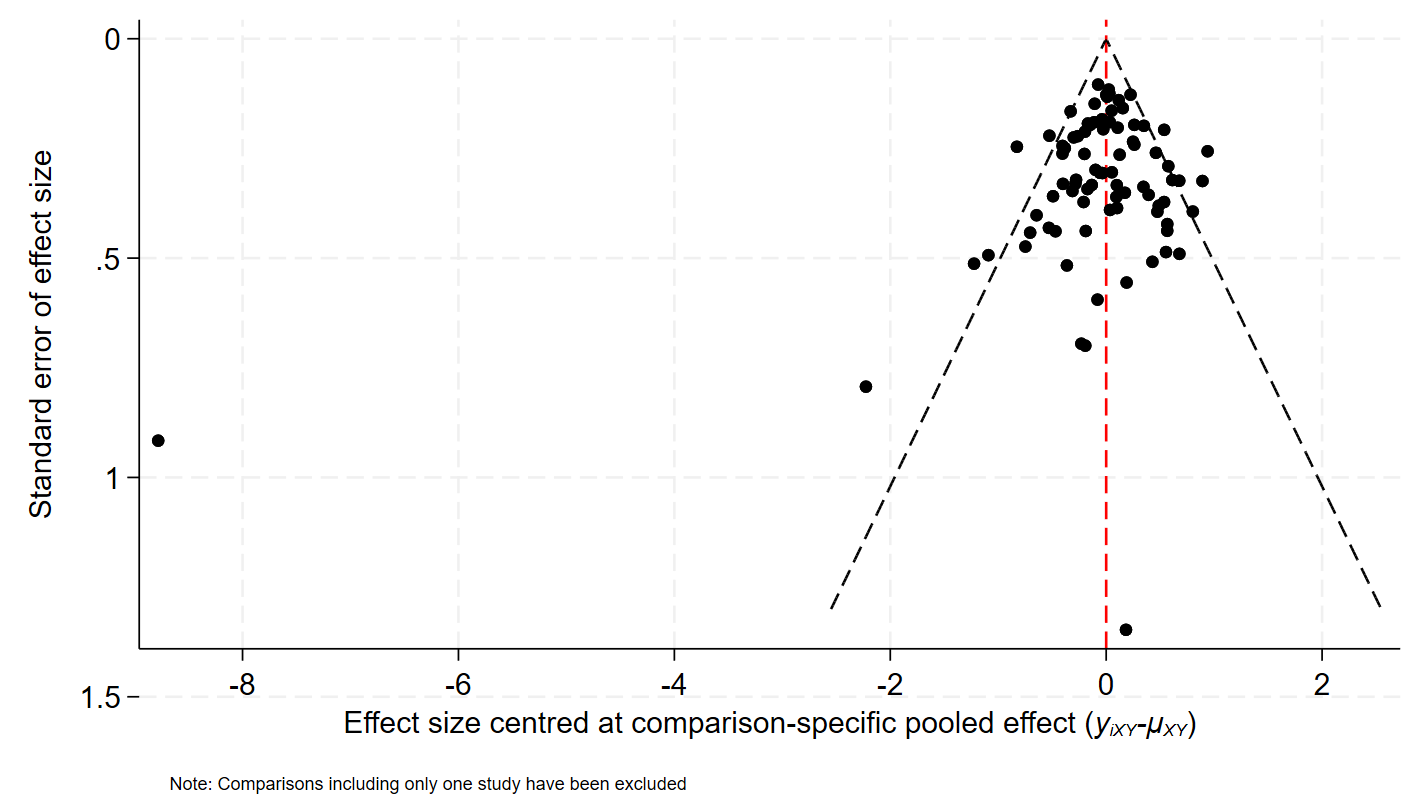


# Supplemental Appendix 9

# Network Meta-Analysis (NMA) Sensitivity Analyses

# For the original NMA, we removed the teaching method combinations which were only represented by a single study. Consequently, seven combinations were dropped from the NMA, resulting in the removal off five studies and seven comparison arms (see table 9.1 below).

# For the first sensitivity NMA, we removed the studies represented in the bottom seven of the SUCRA rankings (*k* = 22, see table 9.2 below) and instead included the teaching method combinations which were only represented by a single study (i.e., those in table 9.1). A comparison of the sensitivity NMA with the original NMA SUCRA results show that four out of the top five teaching method combinations remains consistent (see table 9.3).

# For the second sensitivity NMA, we removed the studies that were considered grey literature (*k* = 3, see table 9.4 below). A comparison of the sensitivity NMA with the original NMA SUCRA results show that all five teaching method combinations remains consistent (see table 9.5).

# For the third sensitivity NMA, we removed the studies that were considered outliers, where the effect size exceeded 3 standard deviations (*k* = 2, see table 9.6 below). A comparison of the sensitivity NMA with the original NMA SUCRA results show that all five teaching method combinations remains consistent (see table 9.7).

# Table 9.1. The teaching method combinations which were removed from the NMA analysis.

| Study Name | Comparison arm(s) removed |  |
| --- | --- | --- |
|  |  |  |
| Bowyer et al., 2010^a^ | O vs. Control  DRO vs. O |  |
|  |  |  |
| Dominick et al., 2018 | HO vs. Control |  |
|  |  |  |
| Grossman et al., 2021 | HROF vs. Control |  |
|  |  |  |
| Hojat et al., 2013^a^ | R vs. Control  DR vs. R |  |
|  |  |  |
| Liu et al., 2016 | HRF vs. Control |  |
|  |  |  |
| Malhotra et al., 2019 | DROF vs. Control |  |
|  |  |  |
| Rassbach et al., 2018 | ROF vs. Control |  |
|  |  |  |

# Abbreviations: vs. = versus; D =Didactic, H =Rehearsal, O = Observation, R =Reflection, F=Feedback

# ^a^ These studies were not removed from the NMA because they were 3 arm studies; therefore only two of the three comparison arms were dropped.

# Table 9.2. Number of studies and their respective teaching method combinations which were removed for the NMA sensitivity analysis, *k* = 22

| Number of Studies | Teaching Method Combination |
| --- | --- |
| 5 | DO |
| 5 | H |
| 3 | HRO |
| 3 | DRO |
| 2 | HR |
| 2 | DF |
| 2 | HF |

# Abbreviations: *k* = number of studies, D =Didactic, H =Rehearsal, O = Observation, R =Reflection, F=Feedback

# Table 9.3. SUCRA rankings of the original analysis compared with the sensitivity analysis.

| Original Teaching Method Combination | SUCRA | Sensitivity Teaching Method Combination | SUCRA |
| --- | --- | --- | --- |
| DH | 96.1 | DH | 94.8 |
| DR | 86.2 | DR | 81.8 |
| DHRO | 65.3 | R | 63.1 |
| DHOF | 64.9 | DHOF | 62.5 |
| DHROF | 58.6 | DHRO | 60.9 |
| DHRF | 56.8 | D | 55.2 |
| D | 55.8 | DHROF | 55.1 |
| DHO | 53.1 | HROF | 53.6 |
| HOF | 51.8 | DHRF | 52.7 |
| DHF | 51.2 | DHO | 49.7 |
| DOF | 47.9 | HOF | 48.8 |
| F | 47.1 | DRF | 47.8 |
| DRF | 46.9 | F | 46.6 |
| DHR | 46.8 | DHR | 45.2 |
| HR | 46 | DOF | 45.2 |
| DF | 44.8 | DHF | 42.8 |
| HF | 39.3 | DROF | 41.6 |
| DO | 38.7 | HO | 38.5 |
| H | 38.5 | HRF | 36.4 |
| HRO | 35.8 | Education As Usual | 35.7 |
| DRO | 35.2 | ROF | 33 |
| Education As Usual | 34.4 | Control | 32.1 |
| Control | 32.8 | Active Control | 26.7 |
| Active Control | 25.7 | DH | 94.8 |

# Abbreviations: D =Didactic, H =Rehearsal, O = Observation, R =Reflection, F=Feedback.

# Table 9.4. The studies that were removed from the NMA analysis.

| Study Name | Comparison arm(s) removed |  |
| --- | --- | --- |
|  |  |  |
| Higgins et al., 1990 | DHROF vs. Control |  |
| Moreland, 1971 | DHROF vs. Active Control |  |
| Reed, 1996 | DHRF vs. Active Control |  |

# Abbreviations: vs. = versus; D =Didactic, H =Rehearsal, O = Observation, R =Reflection, F=Feedback

# Table 9.5. SUCRA rankings of the original analysis compared with the sensitivity analysis where grey literature was removed.

| Original Teaching Method Combination | SUCRA | Sensitivity Teaching Method Combination | SUCRA |
| --- | --- | --- | --- |
| DH | 96.1 | DH | 96.2 |
| DR | 86.2 | DR | 87.7 |
| DHRO | 65.3 | DHRO | 66.2 |
| DHOF | 64.9 | DHOF | 65.9 |
| DHROF | 58.6 | DHROF | 56.7 |
| DHRF | 56.8 | D | 55.5 |
| D | 55.8 | DHO | 52.1 |
| DHO | 53.1 | DHF | 51.8 |
| HOF | 51.8 | HOF | 51.6 |
| DHF | 51.2 | DHRF | 51.2 |
| DOF | 47.9 | HR | 48.4 |
| F | 47.1 | DRF | 48 |
| DRF | 46.9 | DF | 47.3 |
| DHR | 46.8 | F | 47.1 |
| HR | 46 | DHR | 46.9 |
| DF | 44.8 | DOF | 46.1 |
| HF | 39.3 | DO | 40.3 |
| DO | 38.7 | HF | 39.1 |
| H | 38.5 | HRO | 37.9 |
| HRO | 35.8 | Education As Usual | 37.4 |
| DRO | 35.2 | H | 35.9 |
| Education As Usual | 34.4 | DRO | 35.9 |
| Control | 32.8 | Control | 30.1 |
| Active Control | 25.7 | Active Control | 24.8 |

# Abbreviations: D =Didactic, H =Rehearsal, O = Observation, R =Reflection, F=Feedback.

# Table 9.6. The studies that were removed from the NMA analysis.

| Study Name | Comparison arm(s) removed |  |
| --- | --- | --- |
|  |  |  |
| Pirdehghan et al., 2018 | DH vs. Control |  |
| Sanson‐Fisher & Poole, 1978 | DR vs. Control |  |

# Abbreviations: vs. = versus; D =Didactic, H =Rehearsal, R =Reflection

# Table 9.7. SUCRA rankings of the original analysis compared with the sensitivity analysis where outliers were removed.

| Original Teaching Method Combination | SUCRA | Sensitivity Teaching Method Combination | SUCRA |
| --- | --- | --- | --- |
| DH | 96.1 | DHOF | 83.3 |
| DR | 86.2 | DHROF | 73.4 |
| DHRO | 65.3 | DH | 69.4 |
| DHOF | 64.9 | DHRO | 68.5 |
| DHROF | 58.6 | DHRF | 68.3 |
| DHRF | 56.8 | DHO | 62.6 |
| D | 55.8 | DHF | 60.5 |
| DHO | 53.1 | HOF | 58.7 |
| HOF | 51.8 | F | 57 |
| DHF | 51.2 | DRF | 55.3 |
| DOF | 47.9 | DOF | 51.5 |
| F | 47.1 | D | 50.4 |
| DRF | 46.9 | DHR | 47.6 |
| DHR | 46.8 | HR | 47.1 |
| HR | 46 | DF | 46.1 |
| DF | 44.8 | HF | 42.8 |
| HF | 39.3 | DO | 40.2 |
| DO | 38.7 | DRO | 39.1 |
| H | 38.5 | H | 37.5 |
| HRO | 35.8 | DR | 35.7 |
| DRO | 35.2 | HRO | 31.3 |
| Education As Usual | 34.4 | Control | 26.6 |
| Control | 32.8 | Education As Usual | 26 |
| Active Control | 25.7 | Active Control | 21 |

# Abbreviations: D =Didactic, H =Rehearsal, O = Observation, R =Reflection, F=Feedback.

# Supplemental Appendix 10

# Table 1 References

Assing Hvidt, E., Ulsø, A., Thorngreen, C. V., Søndergaard, J., & Andersen, C. M. (2022). Empathy as a learning objective in medical education: using phenomenology of learning theory to explore medical students’ learning processes. *BMC Medical Education*, *22*(1), 628.

Bandura, A., & Walters, R. H. (1977). *Social learning theory* (Vol. 1, pp. 141-154). Englewood Cliffs, NJ: Prentice hall.

Kolb, D. A., Boyatzis, R. E., & Mainemelis, C. (2014). Experiential learning theory: Previous research and new directions. In *Perspectives on thinking, learning, and cognitive styles* (pp. 227-247). Routledge.

Larson, E. B., & Yao, X. (2005). Clinical empathy as emotional labor in the patient-physician relationship. *JAMA*, *293*(9), 1100–1106. https://doi.org/10.1001/jama.293.9.1100

Mezirow, J. (1997). Transformative learning: Theory to practice. New Directions for Adult and Continuing Education, 1997(74), 5–12. <https://doi.org/10.1002/ACE.7401>

Passi, V., & Johnson, N. (2016). The hidden process of positive doctor role modelling. Medical teacher, 38(7), 700-707.

Tennyson, R. D., & Rasch, M. (1988). Linking cognitive learning theory to instructional prescriptions. Instructional Science, 17, 369–385. [https://doi.org/https://doi.org/10.1007/BF00056222](https://doi.org/https:/doi.org/10.1007/BF00056222)

Thurlings, M., Vermeulen, M., Bastiaens, T., & Stijnen, S. (2013). Understanding feedback: A learning theory perspective. Educational Research Review, 9, 1–15. https://doi.org/10.1016/j.edurev.2012.11.004

Wald, H. S., & Reis, S. P. (2010). Beyond the margins: reflective writing and development of reflective capacity in medical education. *Journal of general internal medicine*, *25*(7), 746–749. <https://doi.org/10.1007/s11606-010-1347-4>

Zec, T., & Forrest, D. (2019). Teaching Clinicians About Affect. *Teaching Empathy in Healthcare: Building a New Core Competency*, 85-97.

Supplemental Appendix 11

**Table 11.1.** *Certainty of evidence assessment domains*

| Domain | General Description of Domain | Domain Applied to Pairwise Meta-Analysis | Domain Applied to Network Meta-Analysis |
| --- | --- | --- | --- |
| Risk of Bias | Assesses whether study limitations (e.g., lack of blinding, allocation concealment) may bias the results across the body of evidence. | The risk of bias across included studies | Consider contribution of high/low risk studies to network comparisons |
| Inconsistency | Evaluates the variability of results across studies (e.g., heterogeneity in effect sizes or direction). | *I*², direction of effect | \|  \| \| --- \|  \| Also includes network inconsistency \| \| --- \| |
| Indirectness | Considers whether the evidence directly applies to the review question in terms of population, intervention, comparator, and outcomes. | Relevance of participants/interventions | Includes transitivity assumption (e.g., similarity across treatment comparisons) |
| Imprecision | Assesses whether the confidence intervals are wide or cross thresholds that would change a clinical or policy decision. | Width of CI | \|  \| \| --- \|  \| Considers ranking uncertainty across treatments \| \| --- \| |
| Publication Bias | Examines the likelihood that studies with negative or null results were unpublished or selectively reported, potentially distorting the effect estimate. | Funnel plot, Egger’s test | Uses comparison-adjusted funnel plots and network-wide bias patterns |

**Table 11.2.** *Assessment of certainty of evidence for Pairwise Meta-Analysis (PMA) and Network Meta-Analysis (NMA)*

| Domain | PMA Assessment | PMA Justification | NMA Assessment | NMA Justification |
| --- | --- | --- | --- | --- |
| Risk of Bias: | Low concern | 60/109 studies high risk, but no effect size difference by risk of bias level. | Low concern | See Table 16.3. The majority of comparisons to Control, EAU, and AC included a mix of high- and low-risk studies. Overall, treatment arms were balanced in RoB spread. |
| Inconsistency | High concern | There was substantial heterogeneity, *I*^2^ = 79.19, *p* < .001. Effects varied across studies. | Moderate concern | Global inconsistency test was non-significant, χ^2^ (33) = 39.11, *p* = .21, indicating coherence between direct and indirect estimates. |
| Indirectness | Low concern | Populations and interventions aligned with review objective. Included studies examined similar populations (medical students and physicians), interventions (behavioural empathy training modalities), and outcome measures (empathy-related scales) | Low concern | Network structure well-connected with common comparators (Control, EAU, AC); interventions and outcomes conceptually aligned. |
| Imprecision | Low concern | Overall pooled effect was moderate with narrow 95% CI (*d* = .50 (95% CI = .40, .60) | Moderate concern | Several comparisons had wide CIs; NMA precision was high for more frequent interventions and less certain for less frequent treatment combinations. |
| Publication Bias | Moderate concern | Egger’s test significant (*p* < .001) but funnel plot was fairly symmetrical | Moderate concern | Funnel plot mostly symmetrical a few small-study outliers were identified and addressed in sensitivity analysis. |

**Table 11.3***. Risk of Bias (RoB) spread om control nodes and high-frequency intervention combinations*

| Arm | Number of High Risk Studies | Percentage of High Risk Studies | RoB Spread |
| --- | --- | --- | --- |
| Control | 26 of 47 studies | 55% | Balanced High & Low |
| Education as Usual | 23 of 40 studies | 58% | Balanced High & Low |
| Active Control | 11 of 22 studies | 50% | Balanced High & Low |
| DHROF | 16 of 23 studies | 70% | Mostly High |
| DHRF | 6 of 12 studies | 50% | Balanced High & Low |
| DHF | 3 of 9 studies | 33% | Mostly Low |
| DHOF | 4 of 8 studies | 50% | Balanced High & Low |
| DHR | 2 of 7 studies | 29% | Mostly low |
| DHRO | 4 of 7 studies | 57% | Balanced High & Low |

**Supplemental Appendix 12**

# Figure 12. Frequency of Intervention Combinations by Study

#
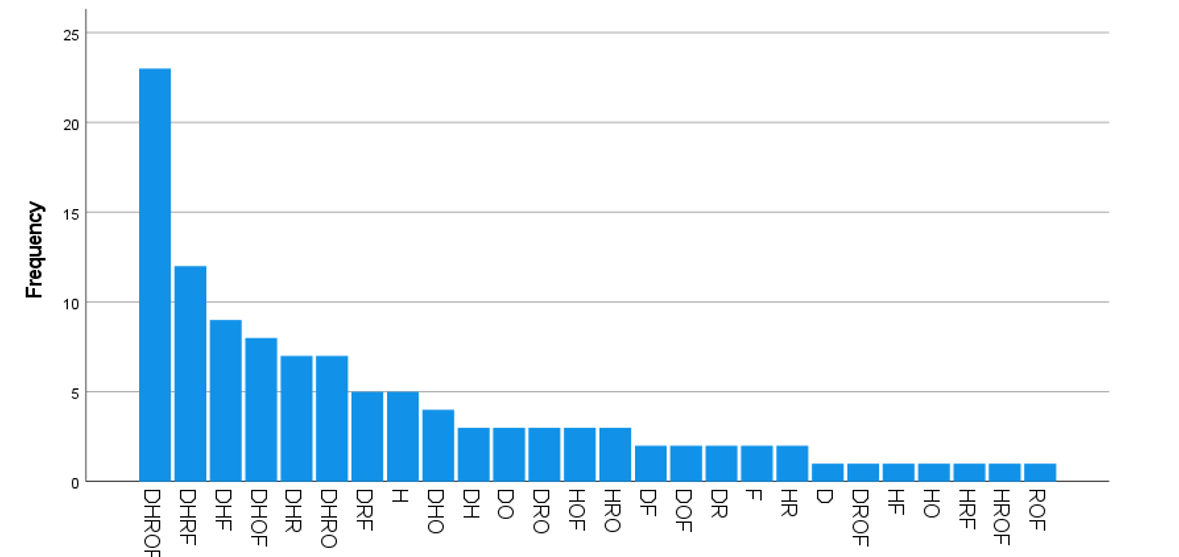


Note: This figure (and corresponding Table 12) summarizes how many studies were included each intervention, irrespective of comparator (pure control, active control, education as usual). These counts do not reflect the statistical contribution of each study to the network meta-analysis estimates. Abbreviations: D =Didactic, H =Rehearsal, O = Observation, R =Reflection, F=Feedback.

**Table 12.** *Frequency and percentages of intervention combinations*

| Intervention  Combination | Frequency of Studies | Percentage (%) |
| --- | --- | --- |
| DHROF | 23 | 20.5 |
| DHRF | 12 | 10.7 |
| DHF | 9 | 8 |
| DHOF | 8 | 7.1 |
| DHR | 7 | 6.3 |
| DHRO | 7 | 6.3 |
| DRF | 5 | 4.5 |
| H | 5 | 4.5 |
| DHO | 4 | 3.6 |
| DH | 3 | 2.7 |
| DO | 3 | 2.7 |
| DRO | 3 | 2.7 |
| HOF | 3 | 2.7 |
| HRO | 3 | 2.7 |
| DF | 2 | 1.8 |
| DOF | 2 | 1.8 |
| DR | 2 | 1.8 |
| F | 2 | 1.8 |
| HR | 2 | 1.8 |
| D | 1 | 0.9 |
| DROF | 1 | 0.9 |
| HF | 1 | 0.9 |
| HO | 1 | 0.9 |
| HRF | 1 | 0.9 |
| HROF | 1 | 0.9 |
| ROF | 1 | 0.9 |

Abbreviations: D =Didactic, H =Rehearsal, O = Observation, R =Reflection, F=Feedback.

**Supplemental Appendix 13**

**Table 13.1. Risk of Bias Coding Criteria^a^**

| Risk of Bias Category | Low Risk | High Risk | Unclear Risk |
| --- | --- | --- | --- |
| Random Sequence Generation (Selection Bias) | The investigators describe a random component in the sequence generation process (e.g., random number table; using a computer random number generator) | The investigators describe a non-random component in the sequence generation process (e.g., sequence generated by date of birth, some rule based on date (or day) of admission) | Insufficient information about the sequence generation process to permit judgement of ‘Low risk’ or ‘High risk’. |
| Allocation Concealment (Selection Bias) | People recruiting/enrolling participants could not foresee assignment because one of the following, or an equivalent method, was used to conceal allocation: central allocation; sequentially numbered, opaque, sealed envelopes. Also, there was no way for participants to know which group they would end up being assigned to when they were signing up. | Participants or investigators enrolling participants could possibly foresee which group they’d be assigned to: using an open random allocation schedule (e.g. a list of random numbers); assignment envelopes were used without appropriate safeguards (e.g. if envelopes were unsealed or non-opaque or not sequentially numbered); alternation or rotation; date of birth; case record number; any other explicitly unconcealed procedure. | Insufficient information to permit judgement of ‘Low risk’ or ‘High risk’. This is usually the case if the method of concealment is not described or not described in sufficient detail to allow a definite judgement – for example if the use of assignment envelopes is described, but it remains unclear whether envelopes were sequentially numbered, opaque and sealed |
| Blinding of Participants and Personnel (Performance Bias) | BOTH (1) Either participants didn’t know there were 2+ different versions of a training being administered and which one they were in because this was not necessarily obvious during recruitment and they are unlikely to talk to people receiving a different version of the intervention (i.e., not from same community) (OR even if participants knew this it is very unlikely this could have influenced the outcome (e.g., their behaviour during outcome measurement) AND (2) Either different people were administering the trainings to the different groups or they didn’t know that the interventions were being compared against each other (i.e., couldn’t have expected better results, or put more effort, into one group relative to another). | EITHER (1) participants knew which version of treatment they were getting, or could easily find out from friends/other participants, and this was likely to be able to affect how they performed on the outcome measurement OR (2) one person administered the different groups and could have trained/treated them differently and introduced bias that would influence the outcome | Insufficient information to permit judgement of ‘Low risk’ or ‘High risk’. |
| Blinding of Outcome Assessment (Detection Bias) | For observational outcomes, the coders/RAs doing the ratings were blinded to participant group status. For self-report outcomes, the participants didn’t know which group they were in or that different groups were being compared. | Coders/research assistants/participants were NOT blind to participant group status, or they were supposed to be blind but blinding was broken, and this could have influenced the outcome. If it was self-report and they know which group they were this is high risk. | Insufficient information to permit judgement of ‘Low risk’ or ‘High risk’. |
| Incomplete Outcome Data (Attrition Bias) | Any one of the following:  • Zero attrition/no missing data;  • Reasons for missing outcome data unlikely to be related to the outcome;  • Missing outcome data balanced in numbers across intervention groups, with similar reasons for missing data across groups;  • The proportion of missing outcomes is negligible compared with plausible effect size;  • Missing data have been imputed using appropriate methods. | Any of the following:  • Either imbalance in numbers or reasons for missing data across groups;  • The proportion of missing outcomes compared with plausible effect size enough to induce clinically relevant bias in observed effect size;  • Analysis compares groups with substantial departure of the intervention received from that assigned at randomization (e.g., put data for people who were supposed to receive intervention but didn’t complete training into the control group);  • Potentially inappropriate application of simple imputation. | Insufficient reporting of attrition/exclusions to permit judgement of ‘Low risk’ or ‘High risk’ (e.g. number randomized not stated, no reasons for missing data provided); |
| Selective Reporting (Reporting Bias) | Either (1) study protocol is available and all pre-specified outcomes that are of interest for the review have been reported in the pre-specified way; or (2) no protocol but obvious that the published reports include all expected outcomes, including those that were pre-specified (uncommon, unless it’s a dissertation with 20 pages of results tables). | Any one of the following:  • any of the study’s pre-specified primary outcomes were not reported;  • one or more primary outcomes is reported using measurements, analysis methods or subsets of the data that were not pre-specified;  • one or more reported primary outcomes were not pre-specified (unless clear justification for their reporting is provided, such as an unexpected adverse effect);  • one or more outcomes of interest in the review are reported incompletely so that they cannot be entered in a meta-analysis;  • the study report fails to include results for a key outcome that would be expected to have been reported for such a study. | Anytime there is no study protocol but nothing that counts as a “high risk” flag. |
| Other Bias | The study appears to be free of other sources of bias (i.e., you have no concerns about any of the potential issues listed below). For example, groups are identical at the start, its funded by a non-profit the author is not affiliated with, and no other risks of confounds/contamination | Study has another risk. For example:  • Study author had a conflict of interest/personal motive to see larger effect sizes (may be related to funding source)  • The randomization was not successful and this was not controlled for in any way  • There was high risk of ‘contamination’ and sharing information across study arms in a way that would influence the outcome (and not captured in performance bias)  • There are major confounding factors that weren’t addressed  • the study has been claimed to have been fraudulent;  • Some other problem. | You suspect another type of bias but don’t have enough information to be able to tell that it would introduce bias OR the study doesn’t give enough information to be able to tell if there are other underlying risks (e.g., short study with not a lot of detail provided). |

Note. Assessments were made on risk of material bias (bias of sufficient magnitude that it would have a notable impact on the results). An “unclear risk” was assigned when the authors did not provide enough information about what happened in the study to make a judgement, or when a risk of bias cannot be assessed even when what happened is known.

^a^ Criteria were based on the Cochrane Risk-of-Bias Tool: Higgins et al. (2011), <https://www.bmj.com/content/343/bmj.d5928>.
